# Multi-phase hybrid metabolomics framework identifies clinically applicable plasma signatures for early detection of gastric cancer

**DOI:** 10.1038/s41467-026-72983-8

**Published:** 2026-05-13

**Authors:** Liyi Bai, Fayong Hu, Weiqin Zhang, Huanqin Peng, Haowen Peng, Huan Li, Xu Zhu, Yibin Xie, Shutian Zhang, Li Min

**Affiliations:** 1 Department of Gastroenterology, Beijing Friendship Hospital, Capital Medical University, Beijing, China; 2 State Key Laboratory of Digestive Health, Beijing, China; 3 National Clinical Research Center for Digestive Diseases, Beijing, China; 4 Beijing Key Laboratory of Early Gastrointestinal Cancer Medicine and Medical Devices, Beijing, China; 5 Department of Gastrointestinal Surgery, Tongji Hospital, Tongji Medical College, Huazhong University of Science and Technology, Wuhan, China; 6 MetWare Biotechnology Co., Ltd., Wuhan, China; 7 Department of Gastrointestinal Surgery, Renmin Hospital of Wuhan University, Wuhan, China; 8 Department of Pancreatic and Gastric Surgery, National Cancer Center, National Clinical Research Center for Cancer, Cancer Hospital, Chinese Academy of Medical Sciences and Peking Union Medical College, Beijing, China; 9 Research Center, Beijing Friendship Hospital, Capital Medical University, Beijing, China

**Keywords:** Tumour biomarkers, Machine learning, Cancer metabolism

## Abstract

Plasma metabolomics offers significant potential for non-invasive biomarker discovery in gastric cancer (GC), yet conventional analytical workflows face challenges in absolute quantification and biological interpretability, hindering clinical translation. Here we present an innovative multi-phase hybrid framework integrating untargeted metabolomics with relative- and absolute-quantitative targeted metabolomics, coupled with a custom interpretability-driven algorithm for de novo biomarker identification. We perform metabolic profiling on 1,706 plasma samples from multicenter cohorts, identifying 84 key metabolites significantly enriched in caffeine metabolism and primary bile acid biosynthesis during the relative quantitation phase. By applying the custom algorithm to absolute quantitation data, we establish a 12-metabolite panel covering multiple functional metabolic modules. Machine learning-based diagnostic models using this signature achieve an area under the curve of 0.951 in validation cohort. Together, our study provides a robust and interpretable framework for translational metabolomics and establishes a GC detection biomarker panel, laying the foundation for future mechanistic research and clinical application.

## Abstract (web summary)

A multi-phase hybrid metabolomics strategy coupled with a custom algorithm has identified a 12-metabolite plasma signature that detects gastric cancer and outperforms conventional tumor markers

## Introduction

Gastric cancer (GC) ranks fifth in global incidence among common malignancies and stands as the third leading cause of cancer-related death worldwide^1^. The five-year survival rate for patients with early gastric cancer (EGC) exceeds 90%^2^, but falls below 10% in those with advanced gastric cancer (AGC)^3^. This striking survival disparity underscores the imperative for early GC detection. While endoscopic examination has been frequently used for early detection of GC due to its diagnostic reliability^4^, the inherent procedural invasiveness and requirement for specialized operator skills constrain its broad clinical implementation. Conventional serum tumor markers (TMs) represent a non-invasive diagnostic approach for GC screening; however, their practical applicability in clinical settings is significantly limited by the intrinsic limitations between sensitivity and specificity^5^. Therefore, the development of noninvasive biomarkers with improved sensitivity and specificity remains critically urgent to achieve precise GC screening.

Metabolomics has emerged as a promising approach for biomarker discovery and mechanistic investigations across diseases. Substantial evidence confirms metabolic perturbations critically drive GC initiation and progression^6,7^, yielding metabolites with high GC specificity. As terminal products of biological processes, metabolites capture physiological changes more sensitively than nucleic acids or proteins, both temporally and spatially^8–10^. Effective metabolomics-driven biomarker discovery fundamentally relies on appropriate detection technologies and analytical frameworks; however, critical design challenges persist in both domains.

Current metabolomic detection generally employs two approaches: untargeted metabolomics enables comprehensive metabolite profiling but with limited quantification confidence^11,12^, while targeted metabolomics provides accurate quantification of predefined metabolites at the cost of restricted analytical breadth^13,14^. These inherent trade-offs necessitate integrated methodologies for rigorous biomarker discovery^15^. In recent years, emerging hybrid platforms (e.g., widely/pseudo-targeted metabolomics) enhance comprehensive metabolite characterization^16^. However, their utility for biomarker discovery and clinical translation remains limited, as both marker selection and model construction rely on relative-quantitative data^17–19^. The absence of absolute quantification not only compromises biomarker validation efficiency and confidence but also necessitates complex cross-platform conversions during model verification, hindering clinical translation. A robust hybrid metabolomics strategy providing both broad metabolite coverage and absolute quantitation, which facilitates reliable GC biomarker discovery, remains unestablished. This persistent gap motivates the development of a hybrid framework designed to bridge these complementary demands.

Regarding analytical frameworks, current metabolomics-based biomarker studies frequently lack biological interpretability. Traditional workflows exhibit overreliance on statistical significance during marker selection, overlooking functional coherence within biological networks^20^. However, metabolites frequently drive disease pathogenesis through coordinated cascade interactions^8,21^. Consequently, neglecting these functional relationships may yield statistically significant biomarkers lacking biological coherence, ultimately compromising their clinical interpretability. Non-negative matrix factorization (NMF), a machine learning technique known for its ability to generate interpretable, additive models through low-rank approximation, has emerged as a powerful tool for advancing precision medicine^22^. Moreover, previous studies have demonstrated its strong potential for elucidating meaningful metabolic pathways from complex datasets^23,24^. Although extensively applied to disease subtyping^25–28^, its utility for metabolite clustering and biomarker discovery remains underdeveloped. Critically, integrating NMF-derived functional modules into biomarker selection pipelines would address limited biological interpretability of traditional workflows, offering a promising strategy for metabolomic biomarker development.

Herein, we proposed a multi-phase hybrid metabolomics strategy that integrated untargeted, relative-quantitative and absolute-quantitative targeted technologies. This approach maintains broad metabolite coverage while progressively refining quantification accuracy, delivering high-quality clinical-grade concentration data for downstream biomarker selection and model construction. Furthermore, we developed a customized interpretability-driven algorithm with NMF, defined as a strategy that prioritizes biological interpretability alongside statistical performance, distinguishing it from traditional statistics-driven methods. We named this algorithm BIO-FIRE (Biomarker Identification and Optimization via Functional and Importance-based Recursive Enhancement) to reflect its integration of functional clustering, statistical importance, and iterative optimization during biomarker selection. Using BIO-FIRE-generated panels, we constructed machine learning (ML)-based diagnostic models for GC across diverse clinical scenarios and rigorously validated their performances in independent cohorts.

## Results

### Patients, data collection and study design

**Fig. 1:**
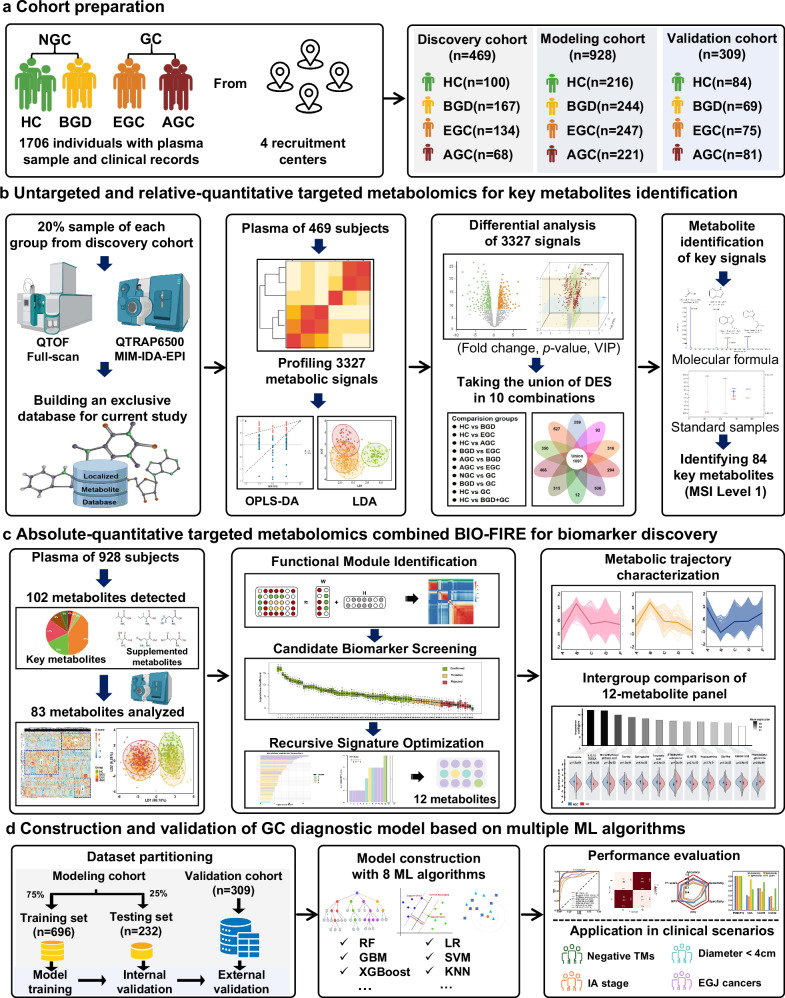
Schematic overview of the study. The illustration was created with a full license on BioRender.com. A total of 1706 participants across three cohorts were included. Untargeted and relative-quantitative targeted metabolomics of the discovery cohort were performed to identify key metabolites. Absolute-quantitative targeted metabolomics (modeling cohort) generated high-quality data, followed by BIO-FIRE analysis to derive metabolic signatures. A 12-metabolite panel (niacinamide, 9,12,13-TODEA, monoethylhexyl phthalic acid, taurine, sphingosine, traumatic acid, 5'-methylthioadenosine, 12-HETE, hypoxanthine, Gly-Phe, azelaic acid, phenylacetylglutamine) was used to establish and optimize GC diagnostic models with eight machine learning algorithms. Model performance was assessed by internal and external validation. The panel’s diagnostic efficacy was evaluated in four challenging clinical scenarios. Abbreviations: GC gastric cancer, NGC non-gastric cancer, HC healthy controls, BGD benign gastric disease, EGC early gastric cancer, AGC advanced gastric cancer, OPLS-DA orthogonal partial least squares discriminant analysis, LDA linear discriminant analysis, DES differentially expressed signals, MSI Metabolomics Standards Initiative, BIO-FIRE Biomarker Identification and Optimization via Functional and Importance-based Recursive Enhancement, ML machine learning, RF random forest, GBM gradient boosting machine, LR logistic regression, SVM support vector machine, KNN k-nearest neighbors, TMs tumor markers, EGJ esophagogastric junction. Source data are provided as a Source Data file.

The overview of this study and the detailed analysis process were schematically shown in Fig. 1. A total of 1706 individuals with plasma samples and clinical records were included from four centers consisting of 826 GC patients and 880 non-GC (NGC) participants. The clinical and pathological characteristics of all patients were summarized in Supplementary Fig. 1. Our hybrid metabolomics strategy comprised three sequential phases (Supplementary Fig. 2): 1) Untargeted phase: preliminary profiling of a discovery cohort subset established a study-specific spectral library through comprehensive metabolic signal detection. 2) Relative-quantitative targeted phase: full discovery cohort analysis characterized plasma metabolic profiles and identified key metabolites meeting Metabolomics Standards Initiative (MSI) Level 1 identification criteria. 3) Absolute-quantitative targeted phase: validation in the modeling cohort focused on prior-phase candidates, ensuring analytical rigor for downstream biomarker selection and modeling.

To enhance biological interpretability and refine biomarker selection, we developed the BIO-FIRE algorithm, which identified a robust 12-metabolite panel. This panel was used to construct GC diagnostic models with eight ML algorithms, and model robustness was validated in an independent cohort. Beyond general diagnosis, we evaluated the panel’s discriminatory power in four clinically challenging subgroups versus NGC controls, including TMs-negative GC, tumors <4 cm in diameter, esophagogastric junction (EGJ) cancers and stage IA GC.

### Global plasma metabolic landscape of GC

**Fig. 2:**
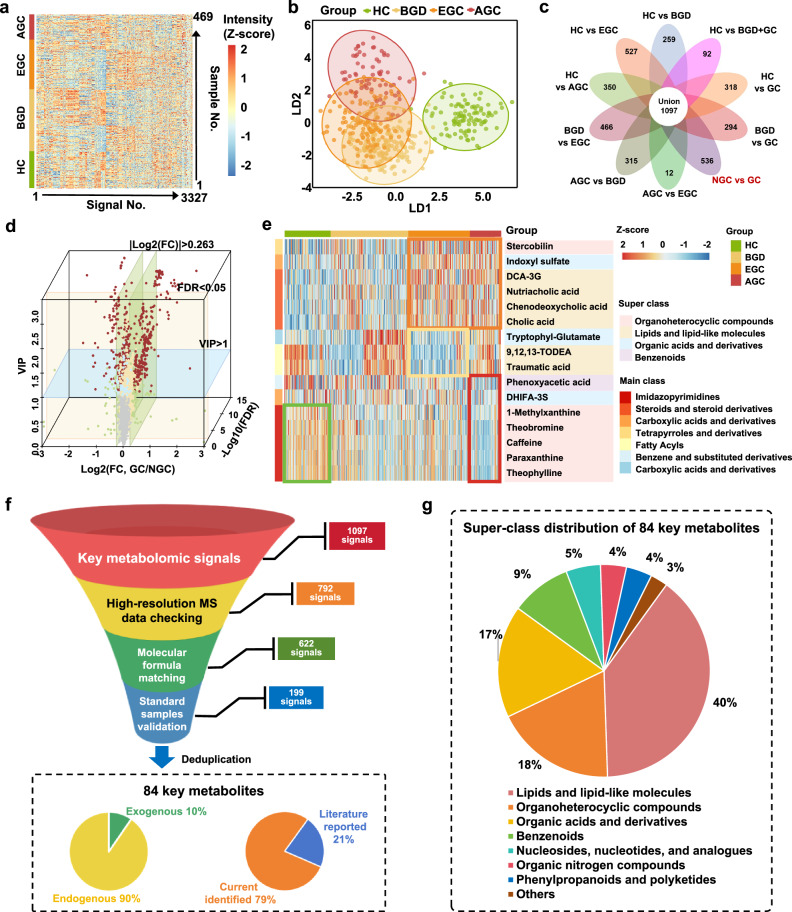
Depiction of GC plasma metabolic landscape for identifying key metabolites. **a** Heatmap of 3327 detected signals across four groups. **b** Linear discriminant analysis (LDA) scatter plot showing distinct group distribution. Ellipses represent the 95% confidence region for each group. **c** Venn diagram for differential metabolic signal identification. **d** Three-dimensional volcano plot identifying differential metabolites between non-gastric cancer (NGC, *n* = 267 biologically independent samples) and gastric cancer (GC, *n* = 202 biologically independent samples) groups in the discovery cohort. Statistical significance was determined using a two-sided Wilcoxon rank-sum test with Benjamini-Hochberg false discovery rate (FDR) correction (screening criteria: fold change (FC) > 1.2 or <1/1.2, FDR-adjusted *P* < 0.05, VIP > 1). **e** Heatmap of top 50 differential signals (by FC) corresponding to 16 metabolites. **f** Funnel plot for metabolomic signal annotation. Pie charts show endogenous/exogenous distribution and prior reporting status of 84 key metabolites. The illustration was created with a full license on BioRender.com. **g** Super-class distribution of 84 key metabolites. Abbreviations: GC gastric cancer, NGC non-gastric cancer, HC healthy controls, BGD benign gastric disease, EGC early gastric cancer, AGC advanced gastric cancer, VIP variable importance in projection FC fold change, FDR false discovery rate, MS mass spectrometry, DCA-3G deoxycholic acid 3-glucuronide, DHIFA-3S dihydroisoferulic acid sulfate. Source data are provided as a Source Data file.

To comprehensively characterize plasma metabolomic profiles of GC patients versus NGC controls, we implemented exploratory untargeted and relative-quantitative targeted metabolomics in the discovery cohort (*n* = 469). Relative-quantitative targeted analysis detected 3327 metabolic signals (Fig. 2a). The derived heatmap revealed distinct metabolic patterns across four groups: healthy controls (HC), benign gastric disease (BGD), EGC and AGC. For effective intergroup discrimination, linear discriminant analysis (LDA) was performed, characterizing overall differences through linear combinations of all compound features (Fig. 2b).

To establish comprehensive intergroup contrasts while ensuring extensive potential biomarker capture, pairwise analyses generated ten comparison sets (Fig. 2c). Differential metabolic signals were identified in each comparison (false discovery rate [FDR] <0.05; fold change [FC] > 1.2 or FC < 1/1.2; variable importance in projection [VIP] > 1), with full results in Supplementary Fig. 3. The union of these comparisons yielded 1097 differentially expressed metabolic signals (defined as key signals), which were then prioritized for structural identification. Given the representation of the overall cohort, the GC vs. NGC comparison was selected to illustrate the screening process with a three-dimensional volcano plot displaying 536 differential signals (Fig. 2d), while the top 50 signals (representing 16 metabolites) ranked by FC were shown in the heatmap (Fig. 2e). Notably, lipids and lipid-like molecules were significantly upregulated in the GC group, whereas organo-heterocyclic compounds were downregulated. Distinct metabolic signatures also distinguished EGC from AGC groups. Overall, these findings were aligned with established reports demonstrating that GC group exhibits distinct metabolic profiles from NGC group. Metabolic heterogeneity between EGC and AGC were also observed, aligning with known metabolic reprogramming events in gastric carcinogenesis.

Figure 2f detailed the identification workflow for all key signals. Adhering to MSI Level 1 criteria^29^, we obtained processed data summarized in source data file. Among 1097 key signals, 792 with high-resolution data advanced to metabolite identification; 622 were confirmed through molecular formula matching and compound verification. Authentic standard validation retained 199 signals, which were deduplicated to yield 84 definitive metabolites designated as GC-associated key metabolites (Supplementary Data 1). Notably, 66 of these 84 metabolites (79%) are identified in this study in addition to previously established GC biomarkers, demonstrating the advantage of our hybrid strategy for de novo metabolic biomarker discovery. Chemical classification revealed predominant lipid, organo-heterocyclic compound, organic acid, and benzenoid categories (Fig. 2g). Pathway enrichment analysis of these key metabolites demonstrated significant involvement in caffeine metabolism and primary bile acid biosynthesis (Supplementary Fig. 4, Supplementary Data 2).

### BIO-FIRE algorithm-driven discovery of metabolic biomarkers

**Fig. 3:**
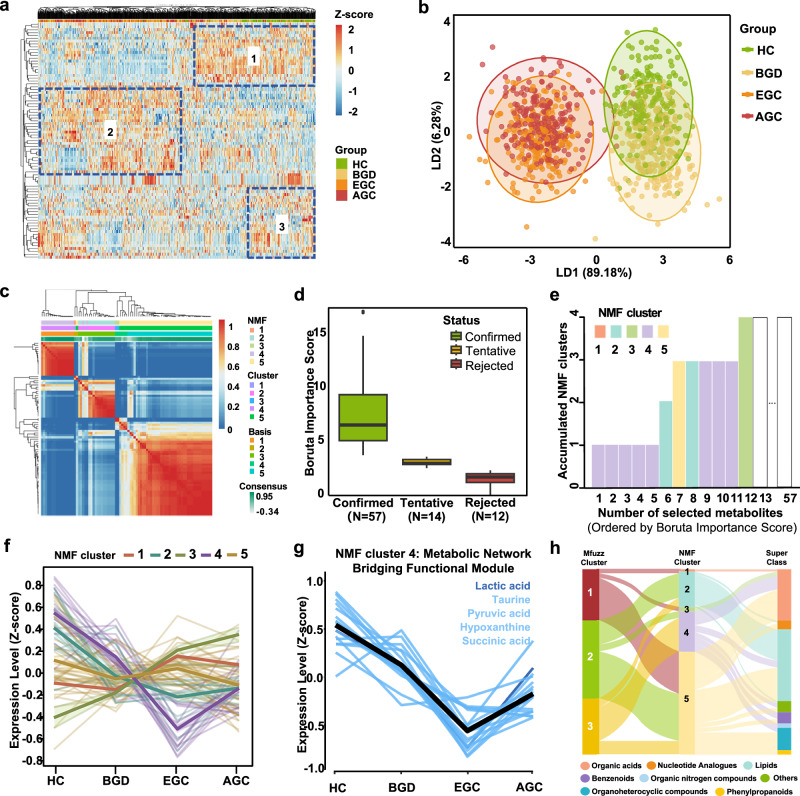
Absolute-quantitative targeted metabolomics leveraging BIO-FIRE algorithm enables biomarker discovery. **a** Heatmap of 83 detected metabolites across groups. **b** Linear discriminant analysis (LDA) scatter plot showing distinct distribution between gastric cancer (GC) and non-gastric cancer (NGC) groups. Ellipses represent the 95% confidence region for each group. **c** Consensus map for non-negative matrix factorization (NMF) clustering of 83 metabolites. **d** Box plot of Boruta feature selection for 57 potential biomarkers. Boxplots show the distribution of mean importance scores for metabolites classified as Confirmed, Tentative, or Rejected by Boruta. The sample size (n) for each box plot represents the number of metabolites in that status group (Confirmed: *n* = 57, Tentative: *n* = 14, Rejected: *n* = 12). Box boundaries: first and third quartiles; center line: median; whiskers extend to 1.5×IQR from the box; points beyond whiskers are outliers. **e** Recursive optimization process identifying 12 final biomarkers. **f**,**g** Metabolic trajectories during disease progression: **f** all NMF clusters; **g** NMF cluster 4, highlighting important metabolites. Shaded ribbons represent the mean ± standard error (SE) of the scaled expression values for metabolites within each NMF cluster. **h** Sankey diagram of associations among Mfuzz cluster, NMF cluster, and metabolite super-class. Abbreviations: GC gastric cancer, NGC non-gastric cancer, HC healthy control, BGD benign gastric disease, EGC early gastric cancer, AGC advanced gastric cancer, NMF non-negative matrix factorization. Source data are provided as a Source Data file.

To acquire clinically applicable concentration data for precise biomarker selection and model construction, we conducted absolute-quantitative targeted metabolomics in the modeling cohort (*n* = 928). The detection panel targeted 84 key metabolites previously identified through relative-quantitative analysis, supplemented by 18 literature-reported GC-associated metabolites (Supplementary Data 3). Following data preprocessing, 83 metabolites demonstrated robust quantifiability across samples and were retained for downstream analyses. Hierarchical clustering based on absolute quantification revealed distinct metabolite clusters with significant alterations (Fig. 3a). LDA further indicated that the primary discriminant component (LD1) explained 89.18% of intergroup variance, confirming significant metabolic divergence among the four groups (Fig. 3b).

We subsequently applied the BIO-FIRE algorithm to identify a robust biomarker panel, ultimately selecting 12 metabolites constituting the final signature (Fig. 3c–e). The selection process comprised three sequential phases: Firstly, unsupervised NMF clustering of all 83 metabolites identified five biologically relevant clusters as the optimized decomposition rank (determined by consensus and residual sum of squares elbow method; Supplementary Fig. 5). Secondly, Boruta algorithm-based feature selection identified 57 metabolites with significantly higher importance scores than shadow features (p < 0.05), designating them as candidate biomarkers (Fig. 3d, Supplementary Fig. 6). Finally, recursive optimization maximized functional coverage of NMF clusters with minimal metabolites: top 5, 6, 7, and 12 metabolites covered 1, 2, 3, and 4 clusters respectively. This yielded the Plasma Metabolic Biomarker—12-metabolite panel (PMB-P12), which simultaneously achieved maximal NMF cluster coverage and minimal biomarker count (Fig. 3e). To rigorously confirm the structural identity of these 12 biomarkers, we performed mirror plots comparing their MS2 spectra in biological samples against authentic standards, confirming high spectral concordance (Supplementary Fig. 7).

### Biological annotation of metabolic modules and PMB-P12

To elucidate the biological interpretability of NMF-derived metabolic functional modules, we integrated the metabolite-module weight matrix (Supplementary Data 4) with pathway enrichment results (Supplementary Fig. 8) for module annotation. Five distinct functional modules corresponding to NMF cluster 1-5 emerged: 1) Glutamine Metabolism; 2) Lipid Metabolism; 3) Host–Microbiome Co-metabolism; 4) Metabolic Network Bridging; 5) Branched-Chain Amino Acid (BCAA) Metabolism. These modules demonstrated significant associations with GC pathophysiology and exhibited characteristic progression trajectories (Fig. 3f, Supplementary Fig. 9), suggesting their involvement in tumor initiation and development.

Among the five functional modules, the BCAA Metabolism module contained the highest metabolite count (n = 46). These metabolites showed significant enrichment in both valine, leucine, and isoleucine biosynthesis and caffeine metabolism pathways, consistent with the pathway enrichment results obtained in the initial relative-quantitative targeted metabolomic analysis. The Metabolic Network Bridging module demonstrated the strongest statistical contribution and exhibited a biphasic trajectory with initial decrease followed by increase during progression (Fig. 3g), suggesting its pivotal role in GC pathogenesis. This 18-metabolite module, featuring lactic acid as a representative component, was enriched in tricarboxylic acid (TCA) cycle, amino acid metabolism, sphingolipid metabolism, and taurine metabolism pathways. Sankey analysis revealed that NMF-derived functional clusters were chemically heterogeneous but partially overlapped with Mfuzz time-series clusters (Supplementary Fig. 10), with most NMF Cluster 4 metabolites mapping to Mfuzz Cluster 3 (Fig. 3h). These findings indicate that the NMF approach not only identifies biologically coherent modules but also captures temporal dynamics, serving as an effective tool for detecting early-stage metabolic alterations in disease progression.

**Fig. 4:**
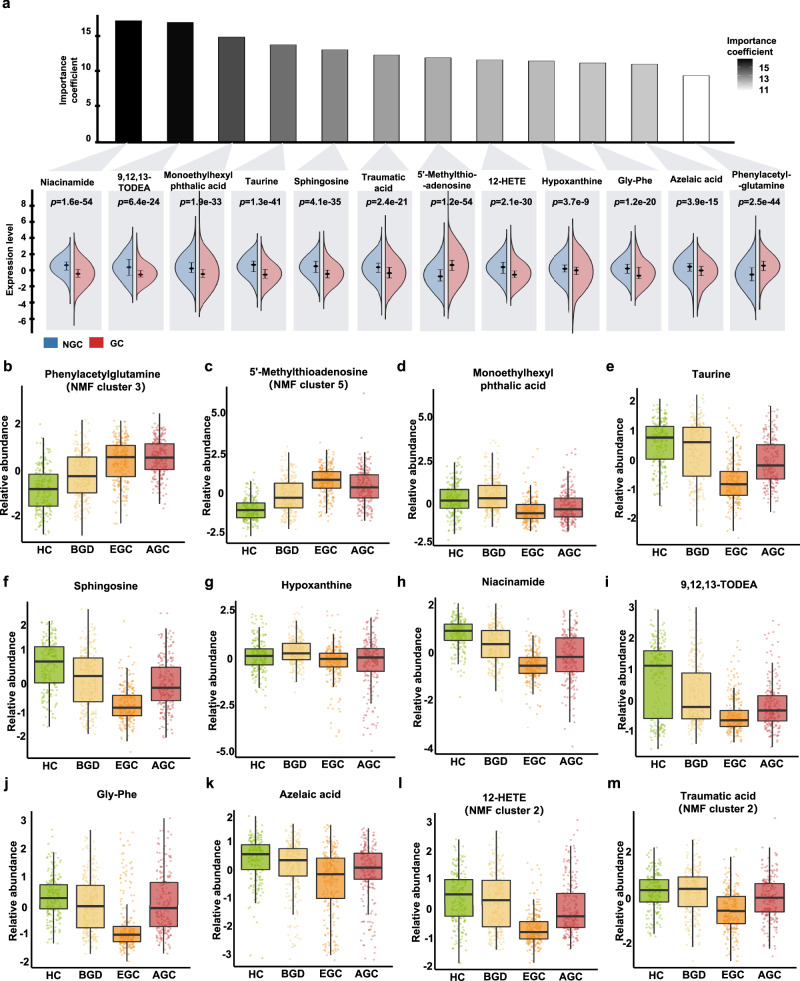
A 12-metabolite biomarker panel for gastric cancer diagnosis. **a** Top: Bar plot of Boruta importance scores for 12 biomarkers. Color intensity (from light to dark) reflects the relative importance score, with darker bars indicating higher importance. Bottom: Split violin plots illustrating the probability density of metabolite expression distributions in gastric cancer (GC, *n* = 468 biologically independent samples) versus non-gastric cancer (NGC, *n* = 460 biologically independent samples) groups from the modeling cohort, where the width of the shaded area represents the proportion of the data located there. Within each violin, the black dot marks the median, and the vertical line spans the interquartile range (IQR) from the 25th to the 75th percentile. Statistical significance was determined using a two-sided Wilcoxon rank-sum test without adjustments for multiple comparisons; exact P values are displayed above each plot. **b–m** Relative abundance (Z-score normalized) of the 12 metabolites across clinical groups: healthy controls (HC, *n* = 216 biologically independent samples), benign gastric disease (BGD, *n* = 244 biologically independent samples), early gastric cancer (EGC, n = 247 biologically independent samples), and advanced gastric cancer (AGC, *n* = 221 biologically independent samples). The metabolites exhibit distinct temporal patterns: **b**,**c** display a sustained monotonic increase with disease progression, while **d–m** follow a biphasic trajectory characterized by a specific depletion in EGC followed by an elevation in AGC (down-up trend). Box plots show NMF cluster annotations (where available); metabolites without annotations belong to cluster 4. For all box plots in (**b–m**), the center line represents the median, the bounds of the box correspond to the 25 and 75th percentiles (first and third quartiles), and the whiskers extend to the minimum and maximum values no further than 1.5 × interquartile range (IQR) from the hinges. Outliers are plotted individually. Abbreviations: GC gastric cancer, NGC non-gastric cancer, HC healthy controls, BGD benign gastric disease, EGC early gastric cancer, AGC advanced gastric cancer, NMF non-negative matrix factorization. Source data are provided as a Source Data file.

Figure 4a detailed the metabolite names of PMB-P12 with their abundance profiles, all exhibiting statistically significant differential expression between GC and NGC groups. Among these, phenylacetylglutamine showed continuous upregulation during GC progression (Fig. 4b), consistent with prior reports^30,31^; 5’-methylthioadenosine (MTA) displayed initial elevation followed by decline across disease stages (Fig. 4c). The remaining 10 biomarkers predominantly exhibited biphasic dynamics—initial downregulation succeeded by upregulation (Fig. 4d–m). Detailed descriptive statistics, fold changes, and significance levels for the 12 biomarkers across all pairwise clinical comparisons, including P-values adjusted for multiple testing via the Benjamini-Hochberg method, are summarized in Supplementary Data 5. These temporal expression patterns provide mechanistic insights into gastric carcinogenesis and establish a framework for identifying stage-specific biomarkers and therapeutic targets.

### Development of ML-based diagnostic models

ML has been widely employed in disease diagnosis due to its robustness, particularly for high-dimensional metabolomic data. To develop efficient GC diagnostic models, we applied eight diverse ML algorithms spanning multiple classification paradigms, constructing models based on the PMB-P12 signature derived from the BIO-FIRE algorithm.

**Fig. 5:**
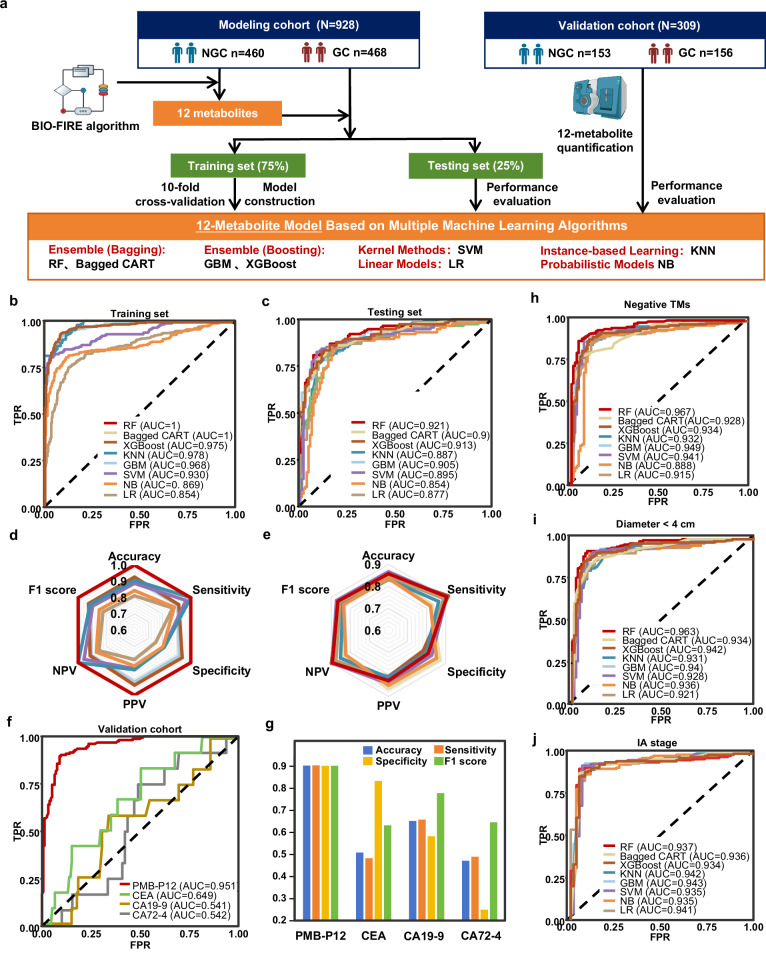
ML-based construction and validation of GC diagnostic model. **a** Overview of the modeling workflow. The illustration was created with a full license on BioRender.com. **b**,**c** Receiver operating characteristic (ROC) curves and area under the curve (AUC) values for eight ML models in training (**b**, *n* = 696 biologically independent samples) and testing (**c**, *n* = 232 biologically independent samples) sets. **d**,**e** Radar plots comparing algorithm performance (accuracy, sensitivity, specificity, positive predictive value [PPV], negative predictive value [NPV], F1 score) in training (**d**) and testing (**e**) cohorts. **f** ROC curves comparing the 12-metabolite panel (PMB-P12) versus clinical tumor markers (CEA, CA19-9, CA72-4) in the validation cohort (*n* = 309 biologically independent samples). **g** Diagnostic metrics (accuracy, sensitivity, specificity, F1 score) for PMB-P12 versus tumor markers. **h-j** ROC analysis of PMB-P12-based ML models in validation subgroups: tumor marker–negative GC (**h**), GC with tumor diameter <4 cm (**i**), and stage IA GC (**j**). Abbreviations: ML machine learning, GC gastric cancer, NGC non-gastric cancer, BIO-FIRE Biomarker Identification and Optimization via Functional and Importance-based Recursive Enhancement, RF random forest, CART classification and regression trees, GBM gradient boosting machine, SVM support vector machine, KNN k-nearest neighbors, LR logistic regression, NB Naïve Bayes, AUC area under the curve, PPV positive predictive value, NPV negative predictive value, FPR false positive rate, TPR true positive rate, PMB-P12 Plasma Metabolic Biomarker—12-metabolite panel, CEA carcinoembryonic antigen, CA19-9 carbohydrate antigen 19-9, CA72-4 carbohydrate antigen 72-4, TMs tumor markers. Source data are provided as a Source Data file.

To enhance models’ robustness, the modeling cohort underwent stratified random splitting into training (75%) and testing (25%) sets. The training set built the diagnostic models, while the testing set evaluated model performance (Fig. 5a). Model efficacy was assessed via area under curve (AUC) and six key metrics: accuracy, sensitivity, specificity, positive predictive value (PPV), negative predictive value (NPV), and F1-score. Most ML models exhibited strong discriminatory power between GC and NGC individuals in training set, with AUCs ranging from 0.854 to 1.000, accuracies from 0.812 to 1.000, sensitivities from 0.872 to 1.000, specificities from 0.751 to 1.000, PPVs from 0.781 to 1, NPVs from 0.852 to 1, and F1 scores from 0.824 to 1. The results demonstrated robustness of the established models in the testing sets, with AUCs ranging from 0.854 to 0.921, accuracies from 0.832 to 0.866, sensitivities from 0.821 to 0.915, specificities from 0.783 to 0.870, PPVs from 0.808 to 0.866, NPVs from 0.824 to 0.900, and F1 scores from 0.835 to 0.872.

Among the eight ML-based models, the random forest (RF) algorithm demonstrated robust diagnostic efficacy in both training set (AUC = 1.000, 95%CI: 1-1.000; accuracy=1.000, 95%CI: 0.995-1.000) and testing set (AUC = 0.921, 95%CI: 0.887-0.956; accuracy=0.858, 95%CI: 0.806-0.900), with the most balanced performance across metrics in radar plot analyses (Fig. 5b-e, Supplementary Fig. 11A). Consequently, we selected the PMB-P12-integrated RF model (PMB-P12-RF) as the final diagnostic model.

### External validation and comparison with clinical tumor markers

To validate the PMB-P12-RF diagnostic model, we performed absolute-quantitative metabolomics targeting the 12 biomarker metabolites in an independent validation cohort (*n* = 309). Inputting individual metabolite concentrations into the established PMB-P12-RF model generated GC probability predictions. The model demonstrated robust diagnostic performance in the validation cohort, achieving an AUC of 0.951 (95% CI: 0.928–0.974), accuracy of 0.903 (95% CI: 0.864–0.934), sensitivity of 0.904 (95% CI: 0.846–0.945), and specificity of 0.902 (95% CI: 0.843–0.944).

Considering clinical TMs, such as carcinoembryonic antigen (CEA), carbohydrate antigen (CA) 19-9, and CA72-4 are also used in GC screening, we further evaluated their performance in our cohorts. Notably, PMB-P12 significantly outperformed all three TMs (Supplementary Fig. 11B). In the validation cohort, CEA had an AUC of 0.581, accuracy of 0.369, sensitivity of 0.278, and specificity of 0.895. CA19-9 yielded an AUC of 0.529, accuracy of 0.302, sensitivity of 0.199, and specificity of 0.895. CA72-4 showed an AUC of 0.556, accuracy of 0.624, sensitivity of 0.722, and a specificity of 0.053. Consistent trends were observed in both the testing set and validation cohort, with all TMs demonstrating lower AUCs, accuracies, sensitivities, and specificities compared to PMB–P12 (Fig. 5f, g, Table 1).

**Table 1:** Classification performance of PMB–P12 and clinical tumor markers in GC patients of different datasets

Dataset	Signature	AUC (95% CI)	Accuracy (95% CI)	Sensitivity (95% CI)	Specificity (95% CI)
Training set	PMB–P12	1.000 (1.000–1.000)	1.000 (0.995–1.000)	1.000 (0.990–1.000)	1.000 (0.989–1.000)
	CEA	0.581 (0.506–0.655)	0.369 (0.320–0.419)	0.278 (0.230–0.330)	0.895 (0.785–0.960)
	CA19–9	0.529 (0.500–0.609)	0.302 (0.256–0.350)	0.199 (0.158–0.247)	0.895 (0.785–0.960)
	CA72–4	0.556 (0.491–0.622)	0.624 (0.573–0.672)	0.722 (0.670–0.770)	0.053 (0.011–0.146)
Testing set	PMB–P12	0.921 (0.887–0.956)	0.858 (0.806–0.900)	0.906 (0.838–0.952)	0.809 (0.725–0.876)
	CEA	0.539 (0.386–0.694)	0.806 (0.727–0.870)	0.891 (0.817–0.942)	0.316 (0.126–0.566)
	CA19–9	0.582 (0.446–0.717)	0.566 (0.476–0.653)	0.545 (0.448–0.641)	0.684 (0.434–0.874)
	CA72–4	0.489 (0.365–0.612)	0.326 (0.246–0.414)	0.227 (0.153–0.317)	0.895 (0.669–0.987)
Validation cohort	PMB–P12	0.951 (0.928–0.974)	0.903 (0.864–0.934)	0.904 (0.846–0.945)	0.902 (0.843–0.944)
	CEA	0.649 (0.500–0.798)	0.509 (0.429–0.589)	0.483 (0.401–0.566)	0.833 (0.516–0.979)
	CA19–9	0.541 (0.379–0.704)	0.652 (0.573–0.725)	0.658 (0.576–0.733)	0.583 (0.277–0.848)
	CA72–4	0.542 (0.402–0.682)	0.472 (0.393–0.552)	0.490 (0.407–0.573)	0.250 (0.055–0.572)

*GC* gastric cancer, *PMB–P12* Plasma Metabolic Biomarker—12-metabolite panel, *CEA* carcinoembryonic antigen, *CA19-9* carbohydrate antigen 19-9, *CA72-4* carbohydrate antigen 72-4, *AUC* area under the curve, *CI *confidence interval.

### Application of PMB-P12 in complex diagnostic scenarios

Beyond general GC diagnosis, we further evaluated the diagnostic performance of PMB-P12 in four clinically critical subgroups: 1) GC patients testing negative for clinical TMs (CEA, CA19-9, CA72-4); 2) GC with tumor diameter <4 cm; 3) GC located at the EGJ, which exhibits distinct biological behavior associated with poorer prognosis; and 4) stage IA GC, representing the absolute indication group for endoscopic submucosal dissection (ESD) where early detection is essential to avoid unnecessary radical gastrectomy. To evaluate PMB-P12’s ability to distinguish each subgroup from NGC subjects, we selected GC patients matching the respective clinical characteristics across all cohorts. Comprehensive results were summarized in Table 2.

**Table 2:** Classification performance of PMB–P12 in GC patients of different datasets under four clinically challenging scenarios

Dataset	Scenario	Accuracy (95%CI)	Sensitivity (95%CI)	Specificity (95%CI)
Training set	Negative TMs	1.000 (0.994–1.000)	1.000 (0.984–1.000)	1.000 (0.989–1.000)
	IA stage	1.000 (0.993–1.000)	1.000 (0.975–1.000)	1.000 (0.989–1.000)
	Diameter <4 cm	1.000 (0.994–1.000)	1.000 (0.984–1.000)	1.000 (0.989–1.000)
	EGJ cancers	1.000 (0.991–1.000)	1.000 (0.934–1.000)	1.000 (0.989–1.000)
Testing set	Negative TMs	0.841 (0.781–0.890)	0.887 (0.814–0.938)	0.770 (0.658–0.860)
	IA stage	0.902 (0.845–0.943)	1.000 (0.926–1.000)	0.861 (0.784–0.918)
	Diameter <4 cm	0.884 (0.829–0.926)	0.973 (0.906–0.997)	0.826 (0.744–0.890)
	EGJ cancers	0.857 (0.786–0.912)	0.889 (0.653–0.986)	0.852 (0.774–0.911)
Validation cohort	Negative TMs	0.889 (0.844–0.925)	0.948 (0.900–0.977)	0.800 (0.708–0.873)
	IA stage	0.903 (0.856–0.939)	0.922 (0.827–0.974)	0.895 (0.836–0.939)
	Diameter <4 cm	0.920 (0.880–0.951)	0.929 (0.858–0.971)	0.915 (0.859–0.954)
	EGJ cancers	0.921 (0.874–0.955)	0.921 (0.786–0.983)	0.922 (0.867–0.959)

*GC* gastric cancer, *PMB-P12* Plasma Metabolic Biomarker—12-metabolite panel, *TMs* tumor markers, *EGJ* esophagogastric junction, *CI* confidence interval.

In TMs–negative GC, PMB-P12 exhibited exceptional diagnostic capacity, achieving AUCs of 0.890–1.000 (training), 0.855–0.923 (testing), and 0.888-0.967 (validation), thereby compensating for diagnostic limitations of conventional serological testing (Fig. 5h, Supplementary Fig. 12A). For sub-4cm tumors, the panel maintained strong discriminatory power with AUCs of 0.890–1.000 (training), 0.881–0.933 (testing), and 0.921–0.963 (validation), overcoming the resolution constraints of standard imaging modalities (Fig. 5i, Supplementary Fig. 12B).

Analysis of stage IA GC subgroups revealed consistently excellent diagnostic capability, with AUCs of 0.896–1.000 (training), 0.897–0.924 (testing), and 0.934–0.943 (validation), fulfilling the need for noninvasive staging prior to ESD (Fig. 5j, Supplementary Fig. 12C). In anatomically distinct EGJ cancers, PMB-P12 exhibited robust diagnostic efficacy, attaining AUCs of 0.882–1.000 (training), 0.848–0.936 (testing), and 0.871–0.957 (validation), confirming conserved metabolic reprogramming in tumors of gastric developmental origin (Supplementary Fig. 12D). These findings suggest that PMB-P12 may have the ability to detect core metabolic alterations occurring early in tumorigenesis rather than secondary changes associated with tumor volume or stage progression, underscoring its significant translational potential.

### Benchmarking of BIO-FIRE against established methods

To validate the methodological superiority of BIO-FIRE, we benchmarked it against established feature selection baselines: LASSO and standard Random Forest-based feature importance ranking. Quantitative analysis revealed that BIO‑FIRE selected a biomarker panel with broader biological coverage (80% for BIO‑FIRE vs. 60% for LASSO), higher current identified rate (72% for BIO‑FIRE vs. 52% for LASSO) and greater chemical diversity (Supplementary Fig. 13A, B). The candidate features identified by BIO-FIRE exhibited the strongest cumulative statistical signals (lowest FDR P-value and highest |Log2FC | ), indicating a prioritization of robust discriminators (Supplementary Fig. 13C). Furthermore, this biologically optimized panel demonstrated superior diagnostic performance in the independent validation cohort (AUC 0.951) compared to baseline methods (AUC 0.946, Supplementary Fig. 13D). Collectively, these quantitative benchmarks demonstrate that BIO-FIRE outperforms established baselines not only in diagnostic accuracy but also in generating a biologically interpretable, diverse, and innovative biomarker panel.

## Discussion

In this study, we conducted comprehensive metabolic landscape profiling and de novo diagnostic biomarker discovery for GC using a multi-phase hybrid metabolomics strategy. Applying our customized BIO-FIRE algorithm, we identified a 12-metabolite panel spanning multiple metabolic functional modules for GC diagnosis. This panel demonstrated superior specificity and sensitivity compared to conventional TMs and exhibited robust diagnostic performance across diverse clinical scenarios. To our knowledge, this represents one GC metabolomics study to both identify biomarkers and develop diagnostic models using absolute quantitation data while achieving broad metabolite coverage, and one to incorporate metabolite functional relationships into the biomarker selection process. Overall, our work addresses key limitations in prior GC metabolomics research through an innovative hybrid framework that includes a customized interpretability-oriented algorithm. This integrative approach provides valuable insights into the early detection and mechanistic understanding of GC, demonstrating promising clinical translation potential.

Plasma metabolomics serves as a powerful tool for biomarker discovery and advancing cancer biology understanding^32^. However, most studies rely on single-platform approaches: untargeted metabolomics enables global profiling but faces low annotation rates compromising biomarker reliability^11,12,33^, while targeted methods provide accurate quantification of known metabolites yet suffer from limited coverage and inadequate metabolite inclusion justification, potentially missing valuable biomarkers^13,14,34^. Recent metabolomic studies have combined untargeted and targeted platforms through parallel^35^, sequential^36^, or pseudo-targeted^17^ approaches. Nevertheless, biomarker selection and model construction in these studies remain conducted on peak area-based relative-quantitation data, with absolute quantitation only been utilized in model validation stages.

Given that relative-quantitative data are susceptible to instrumental drift and noise, and that clinical practice requires absolute concentration measurements, the primary innovation of our study lies in establishing a multi-phase hybrid metabolomics strategy that integrates three complementary technologies, to generate high-quality data for accurate compound identification, de novo biomarker discovery and rigorous model construction. Beyond diagnostic excellence, our framework demonstrated two additional advantages. Firstly, the untargeted and relative-quantitative targeted phases identified 84 key metabolites, with 79% being GC biomarkers that were not previously reported in this context, highlighting efficacy in uncovering previously undetected markers. Secondly, our MSI level 1 compound identification exceed typical hybrid study standards (MSI level 2)^16,18,19^, providing a reliable foundation for absolute quantitation. Building on these 84 metabolites, we finally targeted 102 metabolites (augmented by literature-derived candidates) in the final absolute-quantitative phase. Of these, 83 metabolites (81%) yielded reliable quantitation data, confirming that high-confidence identification supports downstream analysis robustness.

Based on the absolute quantitative data from our hybrid strategy, we introduced a second methodological innovation: the BIO-FIRE algorithm. Unlike conventional black-box feature selection methods, this interpretability-driven workflow integrates biological relevance (via NMF module coverage), statistical importance, and panel conciseness for efficient signature generation. Compared with the 21-metabolite model of Xu et al. (validation AUC 0.94)^37^, our 12-metabolite panel achieved a higher AUC of 0.951 while remaining significantly more concise. Biologically, one upregulated metabolite in the Host–Microbiome Co-metabolic Module (phenylacetyl­glutamine)^30,31^ and three downregulated metabolites in the Metabolic Network Bridging Module (taurine, azelaic acid and hypoxanthine)^38–41^ had been previously reported as GC metabolic biomarkers. The remaining eight metabolites, though identified as GC biomarkers in this study, exhibit functional relevance to carcinogenesis. For instance, 5’-MTA is catabolized by 5’-methylthioadenosine phosphorylase (MTAP) into adenine and 5-methylthioribose-1-phosphate. In various cancers including GC, MTAP is frequently deleted or downregulated, leading to the accumulation of 5’-MTA within tumor cells and the surrounding microenvironment^42–44^, consistent with our finding of elevated 5’-MTA levels in the plasma of GC patients. Moreover, sphingosine modulates gastrointestinal inflammation and plays a dual role in cancer progression: the sphingosine-1-phosphate (S1P)/S1PR axis activates signaling pathways, such as PI3K/Akt to promote tumor development, whereas sphingosine itself exerts anti-tumor effects by inducing apoptosis and pro-inflammatory signaling^45–47^. These potential metabolites may offer additional insights for future mechanistic and therapeutic exploration.

Choosing the appropriate ML algorithm for model construction is also critical for metabolomics biomarker research, yet no consensus exists on the optimal choice^48^. Using the 12-metabolite panel, we benchmarked eight ML algorithms for constructing GC diagnostic models. The AUCs in the testing set ranked as follow: RF > XGBoost > gradient boosting machine (GBM) > bagged CART > support vector machine (SVM) > k-nearest neighbors (KNN) > logistic regression (LR) > Naïve Bayes (NB). Consistent with some studies demonstrating RF’s superiority over other algorithms including SVM^13,49^, our findings further underscored its comparative advantages in clinical metabolomics data analysis. A noteworthy phenomenon is that RF achieved excellent discrimination (AUC = 1.000) on the training set, which indicates a potential overfitting risk. Nevertheless, the algorithm maintained high diagnostic accuracy in both the internal testing set and a completely independent validation cohort. The slightly higher performance in the validation set may be attributed to the focused quantification of the final 12-marker panel, which likely reduced analytical noise compared to the broader profiling phase. This robust external performance substantiates the model’s potential for clinical application, indicating that its strong predictive capability stems from likely capturing reproducible biological patterns rather than cohort-specific noise. Furthermore, our study revealed that ensemble methods, such as bagging or boosting, generally demonstrate enhanced performance in GC metabolomics research. This advantage likely stems from their greater adaptability to the nonlinear relationships and complex interactions inherent in metabolomic datasets^50^. In contrast, algorithms like LR and NB, which rely on assumptions of linearity or feature independence, may struggle with the complexity of metabolomics-driven classification tasks^51^.

Beyond the strong performance in general GC diagnosis, the 12-metabolite panel also demonstrated comparable efficacy when tentatively applied to challenging clinical scenarios. Specifically, the metabolic signature achieved high sensitivity in tumor marker-negative GC (95%) and sub-4cm tumor (93%) subgroups, demonstrating potential to reduce missed diagnoses and improve early detection where conventional methods often fail^52,53^. Unlike prior studies defining EGC as stages I-II^36,54^, we focused on stage IA patients due to its unique clinical implications^55^ and heightened biomarker discovery challenges from subtle metabolic changes^56^. Our study successfully captured metabolites exhibiting significant alterations at the EGC stage, such as lactic acid and taurine, which have been previously reported to have potential for early-stage surveillance^13,38,57^. These valuable findings can likely be attributed to the emphasis on EGC in our study design, including matched sample sizes for EGC and AGC patients and exhaustive pairwise comparisons during key signal identification. Collectively, the panel’s cross-cohort performance confirms the methodological robustness of our hybrid strategy and BIO-FIRE algorithm, demonstrating broad applicability and practical utility in clinical settings.

Several limitations should also be acknowledged. First, while our study demonstrates robust diagnostic performance across independent cohorts from diverse geographical regions within China, we acknowledge that the ethnically homogeneous study population limits immediate global extrapolation. Future validation in multi-ethnic international cohorts is essential to establish its broader clinical applicability. Second, the biological functions of identified metabolic modules and biomarkers lack experimental validation, requiring future mechanistic studies. Third, the retrospective design introduces the potential for uncontrolled confounding factors, incomplete clinical metadata, and limited causal inference; prospective studies with biological validation are required to substantiate observed associations.

In conclusion, our study developed an innovative framework for de novo metabolic biomarker discovery, generating a high-confidence metabolite panel and high-accuracy diagnostic model for early GC detection. The framework holds potential applicability for metabolomics-driven biomarker discovery in other diseases, while the identified metabolites serve a foundation for future mechanistic investigations and clinical translation.

## Methods

### Patient enrollment

This retrospective study recruited a total of 1,706 individuals from four hospital centers across northern to southern China, comprising 826 GC patients and 880 NGC participants. The discovery cohort consisted of 202 GC patients and 267 NGC participants, while the modeling cohort comprised 468 GC patients and 460 NGC participants. Additionally, an independent validation cohort included 156 GC patients and 153 NGC participants. For GC patients, the inclusion criteria were: (1) confirmed GC diagnosis via gastroscopy and pathological examination; (2) blood sample collection before endoscopic or surgical treatment; (3) no prior neoadjuvant therapy; and (4) complete clinical documentation. Exclusion criteria comprised: (1) anticancer therapy (chemotherapy or radiotherapy) within 3 months before blood collection; (2) concurrent systemic malignancies; (3) severe hepatic or renal dysfunction; and (4) pregnancy. EGC was defined as stage I according to the 8th edition of the American Joint Committee on Cancer (AJCC) staging system, while AGC encompassed stage II and beyond. For NGC participants, health status was confirmed through medical history interviews and laboratory examinations. Inclusion criteria for the HC group required the absence of endoscopically confirmed gastric diseases. The BGD group comprised participants with endoscopic evidence of benign gastric lesions (e.g., chronic gastritis, gastric ulcers, gastric polyps). Exclusion criteria for NGC participants were: (1) history of cancer treatment; (2) lack of plasma samples collected before endoscopic examination; (3) severe hepatic or renal dysfunction; and (4) use of medications known to affect hematologic parameters, such as corticosteroids/glucocorticoids. Baseline clinical data, including age, gender, clinical diagnosis, Helicobacter pylori infection status and blood TMs (including CEA, CA19-9, CA72-4), were collected. For GC patients, tumor characteristics including AJCC stage, histological grade, pathological type, tumor location, and tumor size were also recorded. All participants provided written informed consent. This study was approved by the Ethics Committees of Beijing Friendship Hospital (approval number: 2023-P2-266), Cancer Hospital Chinese Academy of Medical Sciences (approval number: 24/485-4765), Renmin Hospital of Wuhan University (approval number: WDRY2025-K042), and Tongji Hospital (approval number: TJ-IRB202412088).

### Plasma sample preparation

Peripheral blood samples were collected from all participants after an overnight fast and prior to any endoscopic examination or surgical intervention. EDTA served as the anticoagulant for all samples. Collected blood samples were transported immediately to the laboratory for processing. Within two hours of collection, plasma was separated by centrifugation at 1800 g for 10 minutes at 4 °C and then immediately stored at −80 °C until metabolite extraction. Standardized collection and processing protocols were followed across all participating centers.

### Metabolite extraction

All extraction procedures were performed on ice. For untargeted and relative-quantitative metabolomic profiling, 50 µL of plasma was mixed with 300 µL of ice-cold methanol. After vortexing, samples were centrifuged at 12,000 rpm for 10 min at 4 °C. Supernatant was vacuum-concentrated and lyophilized extracts stored at −80 °C pending LC-MS/MS analysis. For absolute-quantitative metabolomic profiling, 50 µL of plasma was combined with 150 µL of the extraction solvent and vortexed for 3 minutes. Following centrifugation under identical conditions, 170 µL of the supernatant was loaded onto a dedicated filter-based protein precipitation plate for a secondary clean-up step to effectively remove phospholipids and residual particulates. A positive pressure manifold was then applied to drive the supernatant through the filter membrane, and the purified filtrate was collected directly into a 96-well collection plate. The plate was then sealed and stored pending LC-MS/MS analysis. Quality control (QC) samples were prepared by pooling 20 µL plasma of each person and then processed identical processing.

### Hybrid metabolomics analysis

Our multi-phase hybrid metabolomic framework employed three sequential analytical tiers. The initial comprehensive signal detection phase utilized two complementary untargeted platforms: high-resolution full-scan profiling on TripleTOF 6600 and high-sensitivity Multiple Ion Monitoring-Information Dependent Acquisition -Enhanced Product Ion (MIM-IDA-EPI) analysis on QTRAP 6500^58^. Resultant ion features were integrated with in-house and literature databases to construct a deduplicated gastric cancer-specific plasma metabolic library. Subsequent ion features prioritization and compound identification were performed via relative quantification using MRM mode on QTRAP 6500, refining candidates to GC-associated metabolites. Final absolute quantification employed identical instrumentation with standard calibration curves to generate concentration data for biomarker discovery. Metabolites selected for absolute quantification derived from differential analysis of relative quantification results supplemented by literature-curated metabolite biomarkers of GC (Supplementary Data 3).

Chromatographic separation for targeted analyses used an ACQUITY UPLC HSS T3 column (2.1 × 100 mm, 1.8 μm; Waters) maintained at 40 °C. The gradient program consisted of mobile phase B (0.05% formic acid in acetonitrile) increasing from 5 to 95% over 10 min, holding for 2 min, returning to 5% in 0.1 min, and re-equilibrating for 2.9 min, with mobile phase A (0.05% formic acid in water) at 0.35 mL/min flow rate and 2 μL injection volume. The LC effluent was analyzed using an API 6500 QTRAP LC/MS/MS System (SCIEX) operating in polarity switching mode. Electrospray ionization (ESI) parameters included: 5500 V (positive)/-4500 V (negative) ion spray voltage, 450 °C temperature, 50 psi GS1, 60 psi GS2, and 35 psi curtain gas. MRM detection with optimized declustering potential and collision energy was controlled via Analyst 1.6.3 software.

### Absolute quantitation and internal standard calibration strategy

Mass spectrometry data were processed using MultiQuant 3.0.3 (SCIEX) for chromatographic peak integration and calibration. Peaks were retained only if the signal-to-noise ratio (S/N) exceeded 10 and the retention time (RT) deviation was within 0.1 min. Integrated peak area data were exported for quantitative analysis. To enhance rigorous absolute quantification, we implemented a hybrid calibration strategy. For the majority of metabolites, matched isotope-labeled internal standards (IS) were utilized. Standard calibration curves were constructed by plotting the peak area ratio of the authentic standard to its corresponding IS (Y-axis) against the concentration of authentic standard (X-axis), using a series of standard solutions (Supplementary Data 6). This method effectively corrects for matrix effects and ionization variations. For the remaining metabolites where specific isotope-labeled standards were not commercially available, the external standard method was employed. The reliability of this approach was supported by the proactive phospholipid removal during sample preparation and optimized chromatographic separation on the HSS T3 column, which minimized ion suppression^59,60^. In these cases, calibration curves were generated by plotting the peak area of the authentic standard (Y-axis) directly against its concentration (X-axis). The final analyte concentrations (ng/mL) in plasma samples were calculated by substituting the sample peak response (area or area ratio) into the corresponding linear regression equation, applying a dilution factor of four (Supplementary Data 7).

### Data splitting and preprocessing

To ensure rigorous model evaluation and prevent data leakage, a strict data management protocol was implemented. The modeling cohort (N = 928) was randomly partitioned into a training set (75%) and an internal testing set (25%) using stratified sampling (createDataPartition in R, seed=672) to preserve class balance. All model training and hyperparameter optimization were conducted exclusively within the training set, while the internal testing set was held out solely for final performance evaluation. The external validation cohort (*N* = 309) remained completely blinded to all stages of feature selection and model development.

Data preprocessing was executed in three distinct stages designed to maintain independence. For initial cleaning, metabolite filtering (80% rule, i.e., metabolites detected in fewer than 4/5 of samples were considered undetectable and removed) and missing value imputation (using half the minimum detected value) were performed based on fixed, dataset-wide thresholds. For the BIO-FIRE feature selection phase, row-sum normalization was applied to the entire modeling cohort. This sample-wise operation facilitates stable NMF clustering by mitigating technical variations in total ion intensity without leaking class information. For the final diagnostic model, input features were fixed based on the BIO-FIRE selection. The model was trained directly on absolute quantitative concentrations (ng/mL) without additional scaling or normalization within cross-validation folds. This approach preserves the physical interpretability of the biomarkers and eliminates the risk of leakage associated with fold-dependent statistics.

### Metabolic differential analysis

Metabolic profiles across the four groups (HC, BGD, EGC, AGC) were compared through exhaustive pairwise analysis (10 combinations). Preprocessed relative-quantitative metabolomics data were subjected to differential analysis. Differentially expressed signals were identified using integrated thresholds: 1) Wilcoxon rank-sum test (FDR-adjusted *P* values < 0.05); 2) Fold change (FC) > 1.2 or <1/1.2; and 3) Orthogonal partial least squares discriminant analysis (OPLS-DA)-derived VIP > 1. LDA was applied for dimensionality reduction. Fuzzy c-means clustering was implemented via the Mfuzz R package. KEGG pathway enrichment was performed in MetaboAnalyst 5.0 (www.metaboanalyst.ca), with significance determined by Fisher’s exact test. The union of differentially expressed signals across all comparisons served as key signals. To enhance spectral quality, these signals were further curated using high-resolution MS1 data (from QTOF) and high-sensitivity MS2 spectra (from QTRAP MIM-IDA-EPI). Only features exhibiting high signal-to-noise ratios and distinguishable fragmentation patterns were retained for downstream structural elucidation.

### Compounds identification

For the curated key signals, high-resolution MS data were subjected to ion pair deconvolution to determine accurate neutral masses. Molecular formulas were assigned using PeakView 2.2 (AB SCIEX) with <10 ppm mass accuracy based on exact m/z. Candidate structures matching these formulas were retrieved from ChemSpider and PubChem databases. Structures were prioritized by biochemical relevance and validated through spectral comparison (fragment matching score >70%) in PeakView and manual interpretation of diagnostic fragment ions and neutral losses (e.g., -COOH). Identity confirmation was achieved by analyzing authentic reference standards alongside biological samples. Verification criteria included: 1) RT consistency (±0.2 min); 2) Isotopic distribution pattern match; and 3) MS/MS spectral similarity (dot product >0.8) with <5 ppm mass accuracy. Only metabolites fulfilling all confirmation criteria were designated MSI Level 1 identifications^29^. To benchmark the novelty of identified metabolites, a systematic literature review was conducted searching PubMed, Embase, and ScienceDirect (up to July 2025). A reference set of 243 “known GC-associated metabolites” was compiled from 57 published studies. Novelty was defined as metabolites absent from this cumulative list.

### BIO-FIRE for signature generation

Conceptually, BIO-FIRE is an interpretability-driven framework—an approach that prioritizes biological coherence (via NMF modules) alongside statistical importance, in contrast to black-box methods that select features purely based on numerical thresholds. This design overcomes the limitations of traditional statistics-driven methods (e.g., LASSO), which often select redundant features from dominant pathways. Instead, BIO-FIRE explicitly enforces the selection of biomarkers spanning multiple orthogonal metabolic modules, ensuring comprehensive biological coverage. The BIO-FIRE algorithm integrates NMF and Boruta feature selection within a recursive workflow to generate biologically interpretable biomarker signatures. The process was applied to the entire modeling cohort (*N* = 928) to facilitate the identification of stable and biologically representative metabolic modules. The subsequent model training and validation were then performed on the split datasets and the independent external cohort, respectively.

To facilitate reproducibility, the operational workflow of BIO-FIRE operates through three sequential stages with strictly defined inputs, outputs, and a designated stopping criterion (see flowchart in Supplementary Fig. 14). Briefly, Stage 1 takes row-sum normalized absolute quantitative data as input and outputs biologically orthogonal NMF functional modules. Stage 2 inputs the absolute quantitative data alongside clinical labels to output a narrowed list of statistically significant candidate metabolites. Stage 3 takes these Boruta-ranked candidates and their assigned NMF modules as input to recursively optimize the biomarker panel. This recursive selection terminates upon a strict stopping criterion: when the addition of subsequent ranked metabolites yields no further increase in unique NMF module coverage. The detailed operational principles are described below: 1) Functional Module Identification: NMF was applied to metabolomic data to decompose the non-negative feature matrix into lower-dimensional, biologically coherent functional modules^23^. Each module represents a group of co-regulated metabolites participating in related metabolic pathways, enhancing cluster orthogonality and interpretability. 2) Candidate Biomarker Screening: Boruta, a robust random forest-based algorithm, evaluated the statistical significance of each metabolite^61^. This involved iterative comparison of feature importance against permuted synthetic noise features (“shadows”). Only metabolites demonstrating significantly higher importance than their shadow counterparts (*P* < 0.05) were retained, mitigating noise and selection bias while improving model stability. 3) Recursive Signature Optimization: Candidate metabolites, ranked by Boruta-derived importance scores, were incorporated sequentially into the signature. At each iteration, the incremental improvement in functional module coverage (i.e., the proportion of NMF-derived modules represented by ≥1 selected biomarker) was assessed. Selection terminated upon reaching a predetermined maximal coverage threshold, ensuring each added biomarker contributed unique biological context and complemented prior selections. Collectively, BIO-FIRE systematically balances biological relevance (via NMF modules), statistical robustness (via Boruta), and parsimony (via recursive coverage optimization) to derive concise, interpretable, and translationally viable biomarker panels.

### ML-based diagnostic model

The modeling cohort was randomly partitioned into training (75%) and testing (25%) subsets via stratified sampling using the createDataPartition function from the R caret package, preserving class distributions. Reproducibility was ensured through fixed random seeding. Model performance was assessed using multiple machine learning algorithms representing diverse computational paradigms including ensemble methods (e.g., RF, bagged CART, GBM, XGBoost), kernel-based method (e.g., SVM-Radial), linear classification (e.g., LR), instance-based model (e.g., KNN) and probabilistic model (e.g., NB). This selection encompassed both classical and contemporary algorithms spanning parametric and non-parametric approaches. For hyperparameter tuning, a grid search was implemented using the caret package (version 7.0.1) in R. The tuning process employed 10-fold cross-validation, with the optimal hyperparameter combination for each algorithm being selected based on the highest average classification accuracy across the cross-validation folds. The complete hyperparameter search grids and the final selected values for all eight algorithms are detailed in Supplementary Data 8.

### Bioinformatics and statistical analyses

Bioinformatics and statistical analyses were comprehensively detailed throughout the Results section, corresponding figure captions, and dedicated Methods subsections. All computational workflows were implemented in R (version 4.4.2) leveraging critical packages including NMF (version 0.28) for consensus clustering, Boruta (version 9.0.0) for feature selection, Mfuzz (version 2.66.0) for time-series analysis, and caret (version 7.0.1) for machine learning model training and hyperparameter tuning. Additionally, the pheatmap (version 1.0.12) package was used for heatmap visualization, MASS (version 7.3.65) for LDA, pROC (version 1.18.5) for receiver operating characteristic curve (ROC) evaluation, and ggplot2 (version 3.5.2) for graphical representations. For group comparisons of unpaired samples, statistical significance was determined using two-sided Wilcoxon rank-sum tests with Benjamini-Hochberg false discovery rate correction. Diagnostic performance of the predictive model was quantified through AUC with 95% CIs computed via binomial exact tests, where optimal classification thresholds were established through Youden index maximization. Comprehensive performance metrics including accuracy, sensitivity, specificity, PPV, NPV, and F1-score were subsequently calculated. Overall, the threshold of statistical significance was set with a *P* value < 0.05.

### Reporting summary

Further information on research design is available in the Nature Portfolio Reporting Summary linked to this article.

## Supplementary information

Supplementary Information

Transparent Peer Review file

Reporting Summary

Description of Additional Supplementary Files

Supplementary Data 1–8

## Source data

Source Data

## Data Availability

Data supporting the graphs are provided in the Source Data file. All raw and processed data are included in the Supplementary Information and Supplementary Data files. The metabolomic mass spectrometry data generated in this study have been deposited in the ProteomeXchange Consortium via the iProX partner repository^62,63^ with the dataset identifier PXD071237. Source data are provided with this paper.

## Code availability

The custom code developed for this study is available on GitHub at https://github.com/liyibai123/GC_Metabolomics and has been archived on Zenodo with the 10.5281/zenodo.19426005.
