# Supplementary material for: Multi-phase hybrid metabolomics framework identifies clinically applicable plasma signatures for early detection of gastric cancer
Source: Nat Commun. 2026 May 13;17:6372. doi: 10.1038/s41467-026-72983-8 (PMC13376920; doi:10.1038/s41467-026-72983-8)
Supplement: Supplementary file 1 — Supplementary Information [file 41467_2026_72983_MOESM1_ESM.pdf]

Multi-phase hybrid metabolomics framework identifies clinically applicable plasma signatures for early detection of gastric cancer

Liyi Bai[1,2,3,4#], Fayong Hu[5#], Weiqin Zhang[6], Huanqin Peng[6], Haowen Peng[6], Huan Li[6], Xu Zhu[7*], Yibin Xie[8*], Shutian Zhang[1,2,3,4*], Li Min[1, 2,3,4,9*]

1. Department of Gastroenterology, Beijing Friendship Hospital, Capital Medical University, Beijing 100050, China
2. State Key Laboratory of Digestive Health, Beijing 100050, China
3. National Clinical Research Center for Digestive Disease, Beijing 100050, China
4. Beijing Key Laboratory of Early Gastrointestinal Cancer Medicine and Medical Devices, Beijing 100050, China
5. Department of Gastrointestinal Surgery, Tongji Hospital, Tongji Medical College, Huazhong University of Science and Technology, Wuhan 430030, China
6. MetWare Biotechnology Co., Ltd., Wuhan 430075, China
7. Department of Gastrointestinal Surgery, Renmin Hospital of Wuhan University, Wuhan 430000, China
8. Department of Pancreatic and Gastric Surgery, National Cancer Center, National Clinical Research Center for Cancer, Cancer Hospital, Chinese Academy of Medical Sciences and Peking Union Medical College, Beijing, 100021, China.
9. Research Center, Beijing Friendship Hospital, Capital Medical University, Beijing 101318, China

[#] These authors contributed equally.

***Corresponding Authors:**

Li Min (minli@ccmu.edu.cn); Shutian Zhang (zhangshutian@ccmu.edu.cn); Yibin Xie (yibinxie@cicams.ac.cn); Xu Zhu (zhuxuwhu@whu.edu.cn)

Supplementary Information

Supplementary Figures:

Supplementary Fig. 1 Clinical and pathological characteristics of the study cohort.

Supplementary Fig. 2 Schematic workflow of the multi-phase hybrid metabolomics strategy.

Supplementary Fig. 3 Identification of differential metabolic signals across pairwise comparisons.

Supplementary Fig. 4 Pathway enrichment analysis of the 84 key GC-associated metabolites.

Supplementary Fig. 5 Determination of the optimal factorization rank for NMF clustering.

Supplementary Fig. 6 Feature importance ranking and selection using the Boruta algorithm.

Supplementary Fig. 7 Representative MS/MS mirror plots verifying the structural identity of the 12 diagnostic biomarkers.

Supplementary Fig. 8 Biological functional annotation of NMF-derived metabolic modules via KEGG enrichment.

Supplementary Fig. 9 Longitudinal metabolic trajectories of NMF functional modules during gastric carcinogenesis.

Supplementary Fig. 10 Metabolic trajectories in Mfuzz temporal clusters during gastric carcinogenesis.

Supplementary Fig. 11 Performance evaluation of machine learning models and comparison with tumor markers.

Supplementary Fig. 12 Diagnostic performance of the PMB-P12 panel in clinically challenging subgroups.

Supplementary Fig. 13 Comprehensive benchmarking of the BIO-FIRE algorithm against baseline feature selection methods.

Supplementary Fig. 14 Flowchart illustrating the BIO-FIRE biomarker discovery framework.

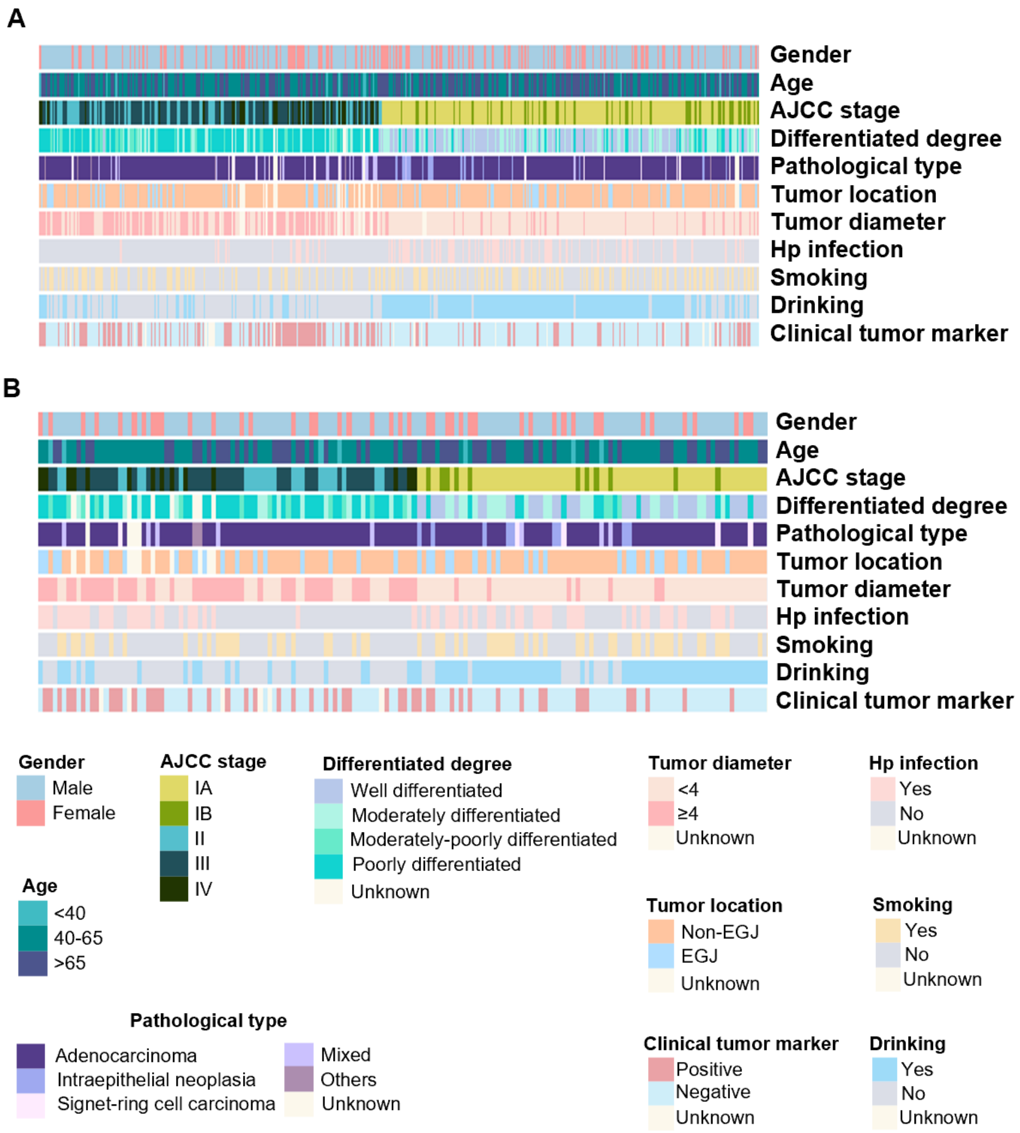

Supplementary Fig. 1 Clinical and pathological characteristics of the study cohort. (**A**) Modeling cohort (GC, n=468). (**B**) Validation cohort (GC, n=156). Abbreviations: GC, gastric cancer; AJCC, American Joint Committee on Cancer; EGJ, esophagogastric junction. Source data are provided as a Source Data file.

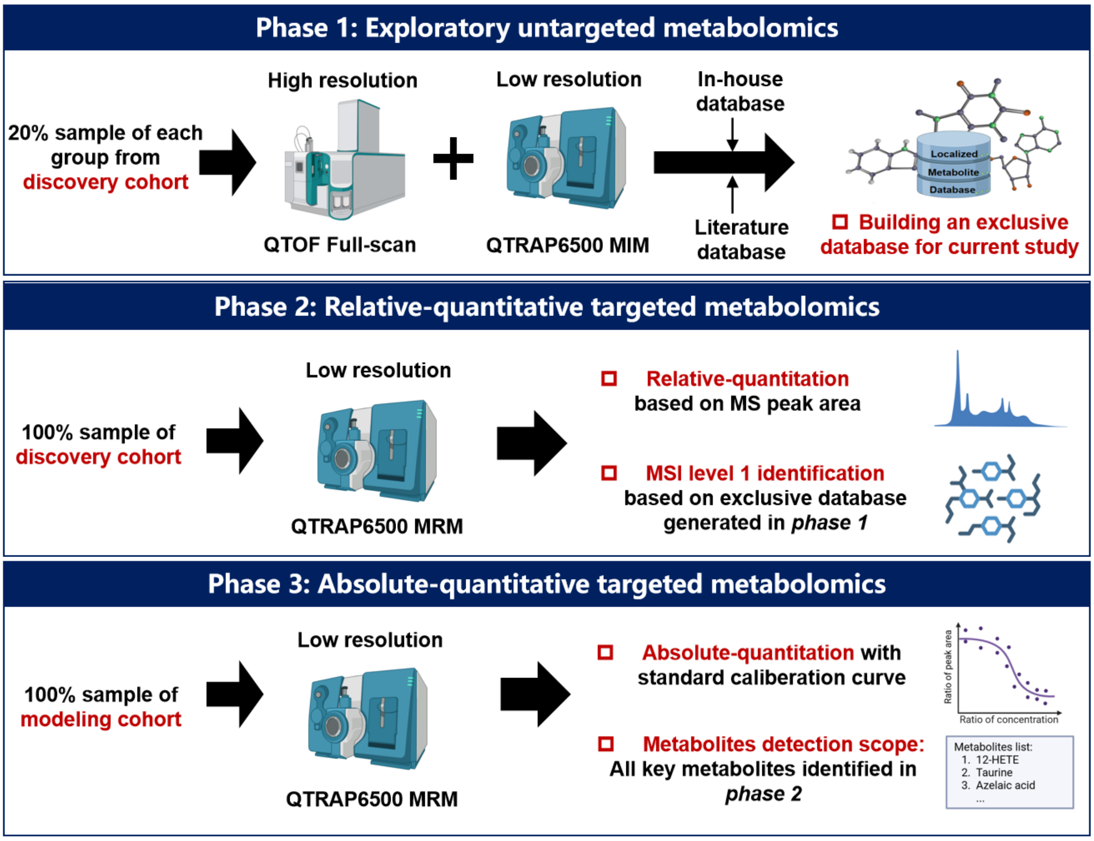

Supplementary Fig. 2 Schematic workflow of the multi-phase hybrid metabolomics strategy. The illustration was created with a full license on BioRender.com. Abbreviations: QTOF, quadrupole time-of-flight; MIM, multiple ion monitoring; MRM. multiple reaction monitoring; MS, mass spectrometry; MSI, metabolomics standards initiative.

Technical details of MIM-IDA-EPI analysis: The Multiple Ion Monitoring (MIM) scan served as a survey scan to trigger the Information-Dependent Acquisition (IDA) of Enhanced Product Ion (EPI) spectra. In the MIM mode, a minimal collision energy (CE) of 5 eV was applied in Q2, allowing precursor ions isolated in Q1 to pass through to Q3 without fragmentation for detection. Upon exceeding an intensity threshold, EPI scans were triggered and acquired with a declustering potential (DP) of 40 V and CE of 40 V to generate MS/MS spectra. A stepwise MIM-EPI strategy was adopted covering a mass range from m/z 50 to 1000 in positive ion mode. The scan range was segmented with a step size of 1 Da (e.g., 50/50, 51/51... to 1000/1000 Da). Each MIM transition was monitored with a 5-ms dwell time and a 5-ms pause time. Each experiment monitored 30 MIM transitions, with product ions scanned from 50 to 1000 Da in Q3. The total cycle time for one scan was approximately 1.9 s. In total, 32 LC/MS runs were performed to cover the entire mass range.

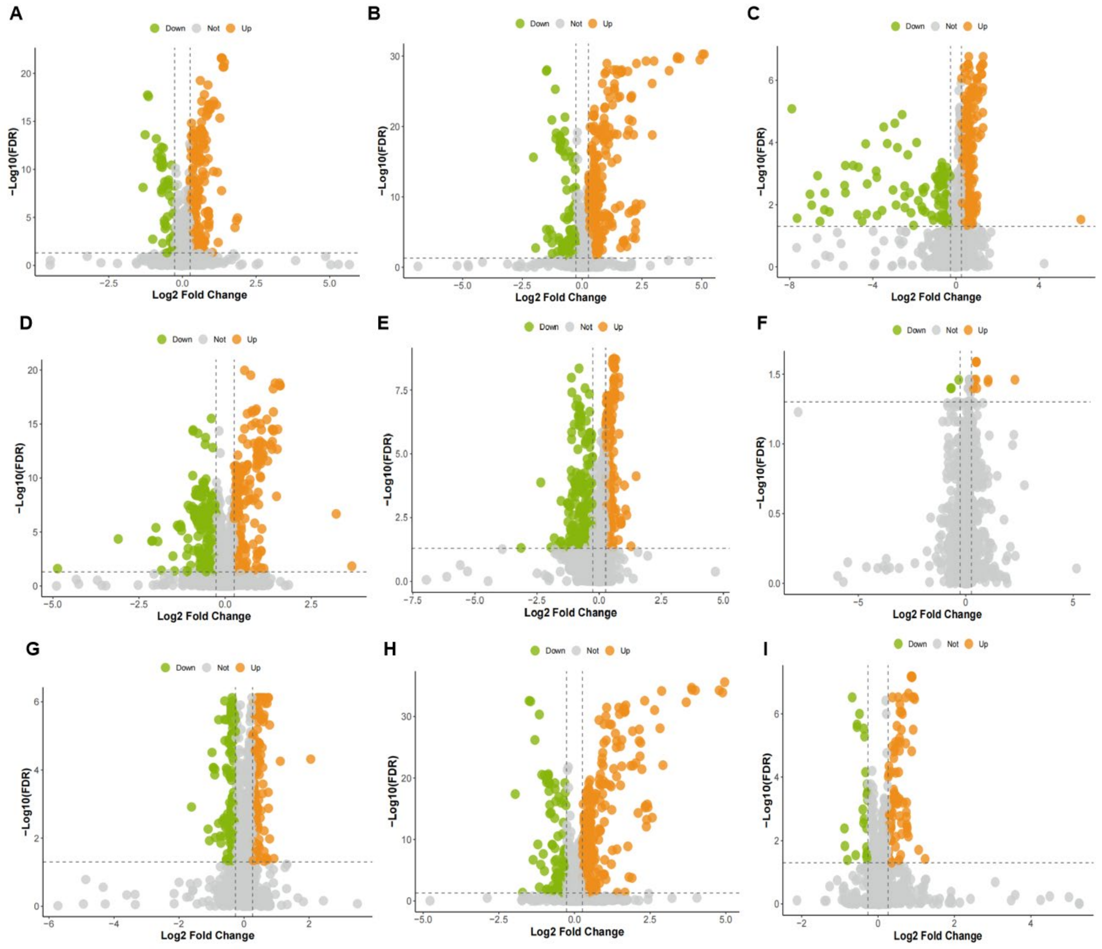

Supplementary Fig. 3 Identification of differential metabolic signals across pairwise comparisons. Volcano plots for group comparisons: (**A**) HC vs. BGD, (**B**) HC vs. EGC, (**C**) AGC vs. HC, (**D**) EGC vs. BGD, (**E**) BGD vs. AGC, (**F**) EGC vs. AGC, (**G**) GC vs. BGD, (**H**) GC vs. HC, (**I**) GC+BGD vs. HC. Each volcano plot compares the indicated groups using two-sided Wilcoxon rank-sum tests with Benjamini-Hochberg FDR correction. The exact n numbers for each group are: HC n=216, BGD n=244, EGC n=247, AGC n=221, GC n=468, NGC n=460 (modeling cohort). Screening criteria: false discovery rate (FDR) adjusted *P* value < 0.05, fold change (FC) > 1.20 or < 1/1.20, variable importance in projection (VIP) > 1. Abbreviations: HC, healthy controls; BGD, benign gastric disease; EGC, early gastric cancer; AGC, advanced gastric cancer. Source data are provided as a Source Data file.

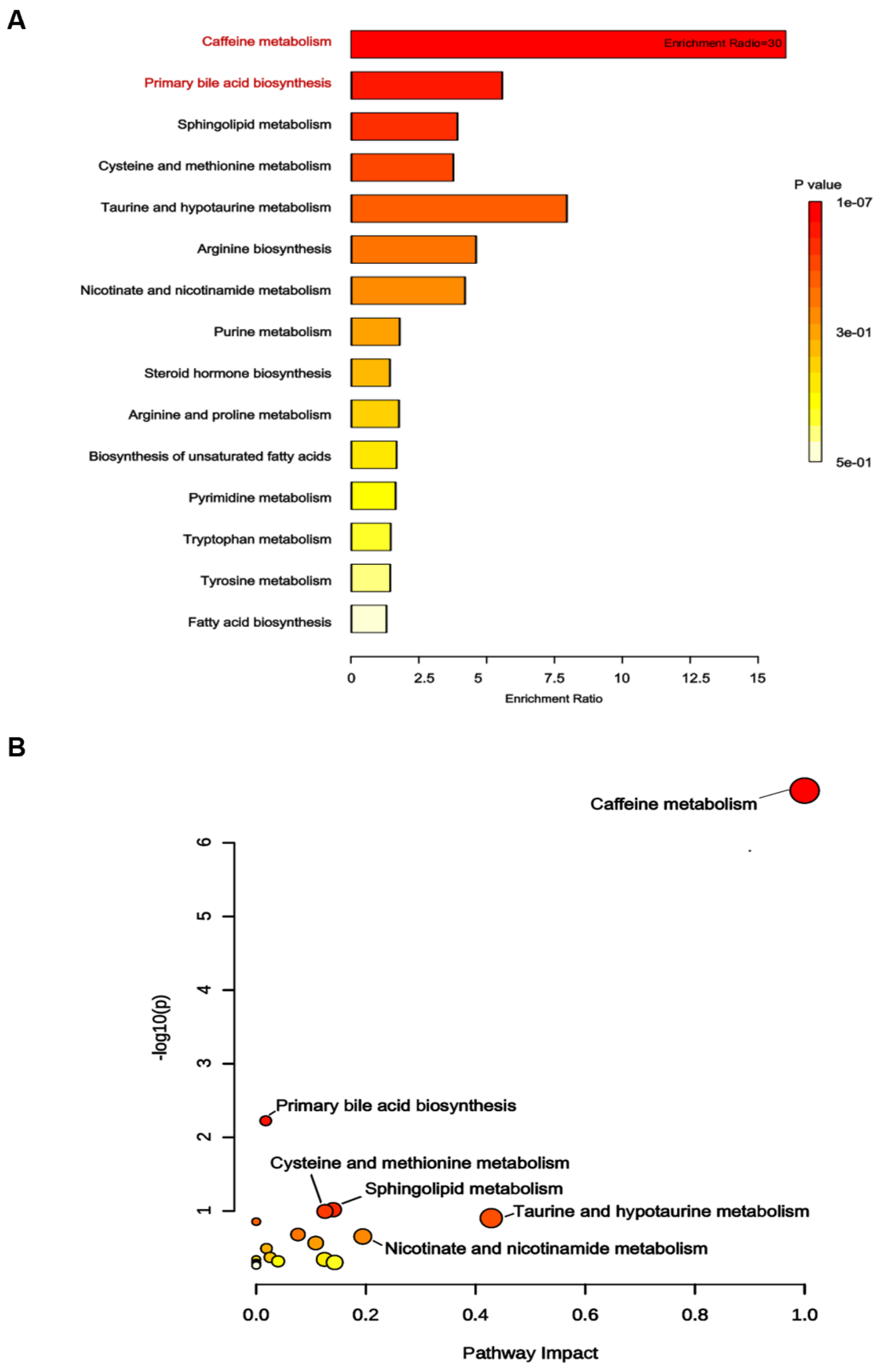

Supplementary Fig. 4 Pathway enrichment analysis of the 84 key GC-associated metabolites. (**A**) KEGG enrichment analysis of 84 metabolites. (**B**) Topology analysis of relevant metabolic pathways. Abbreviations: GC, gastric cancer. Source data are provided as a Source Data file.

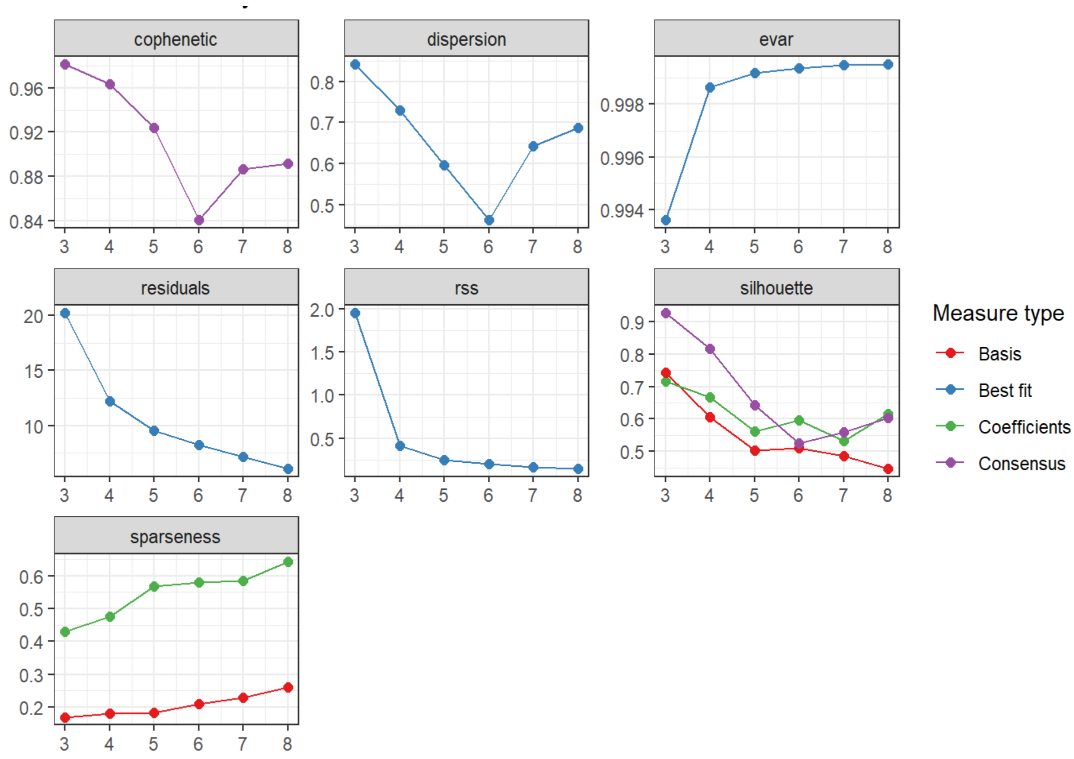

Supplementary Fig. 5 Determination of the optimal factorization rank for NMF clustering. Optimal rank selection (k=5) based on consensus of: cophenetic coefficient, silhouette width, sparseness, and residual sum of squares (RSS) elbow point. Metabolites classified into five biologically relevant NMF clusters. Abbreviations: NMF, non-negative matrix factorization. Source data are provided as a Source Data file.

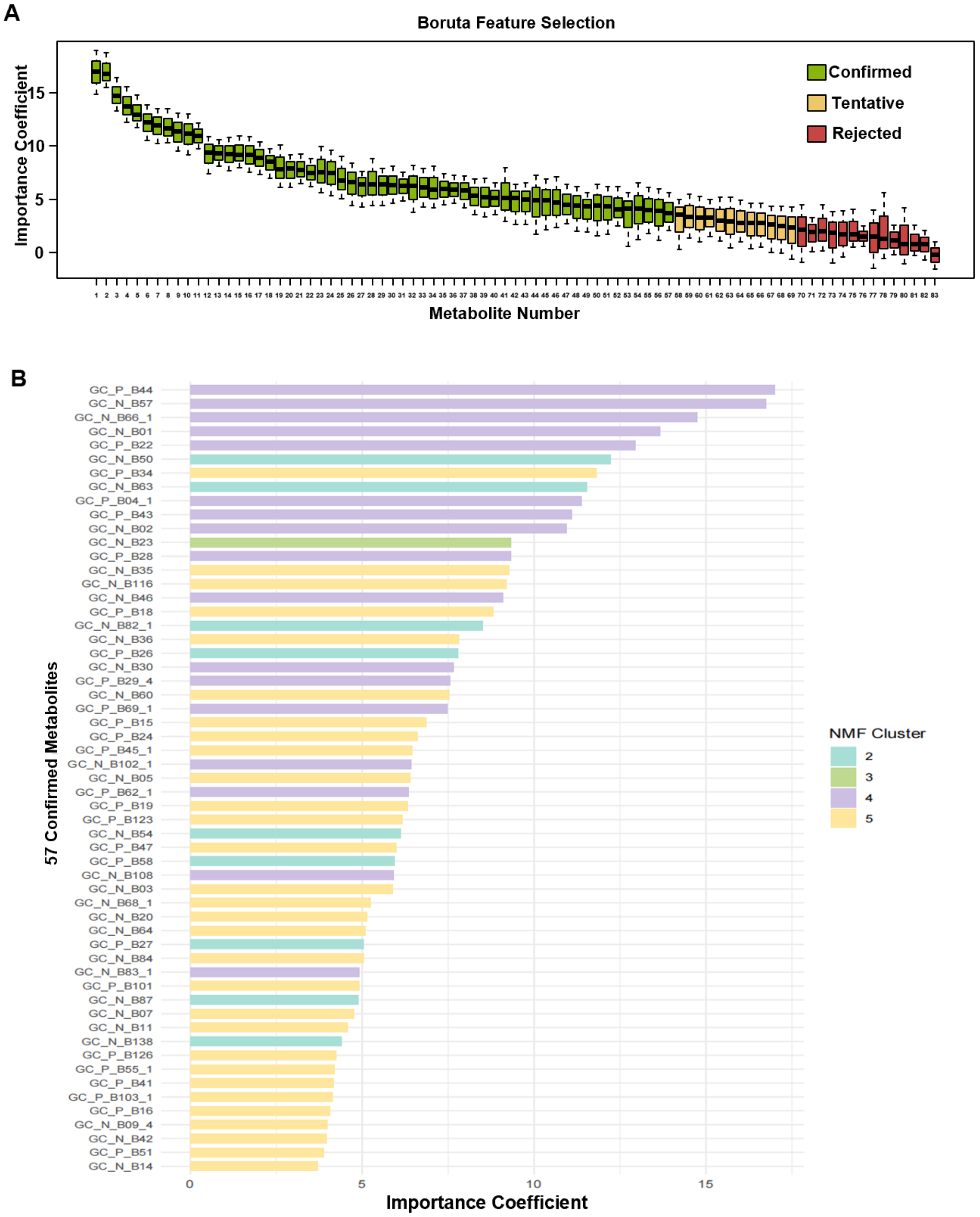

Supplementary Fig. 6 Feature importance ranking and selection using the Boruta algorithm. (**A**) Feature importance ranking of 83 metabolites. For each metabolite, a standard boxplot is drawn based on four input values: minimum, median, mean, and maximum. Box: 25th–75th percentiles; center line: median; whiskers: min–max. Colors: Confirmed (green, n=57), Tentative (yellow, n=14), Rejected (red, n=12). Metabolites are ordered from top to bottom by decreasing mean importance. (**B**) Importance scores of 57 confirmed biomarkers. Bar colors indicate NMF cluster membership. Abbreviations: NMF, non-negative matrix factorization. Source data are provided as a Source Data file.

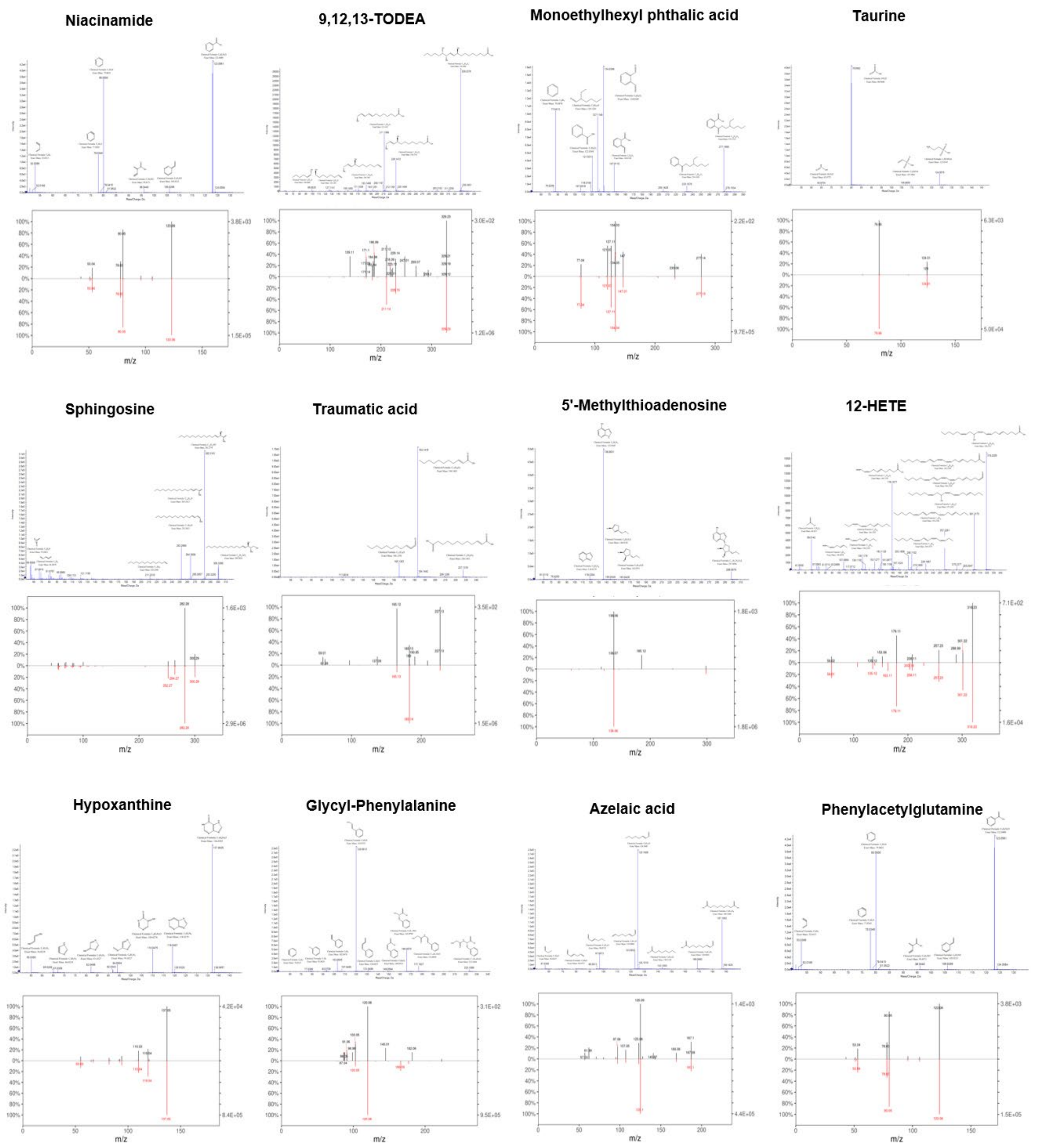

Supplementary Fig. 7 Representative MS/MS mirror plots verifying the structural identity of the 12 diagnostic biomarkers. The MS2 fragmentation spectra from the plasma samples (top blue panels) show a high degree of similarity to the spectra obtained from authentic chemical standards (bottom red panels), confirming the structural identification at Metabolomics Standards Initiative (MSI) Level 1 standard.

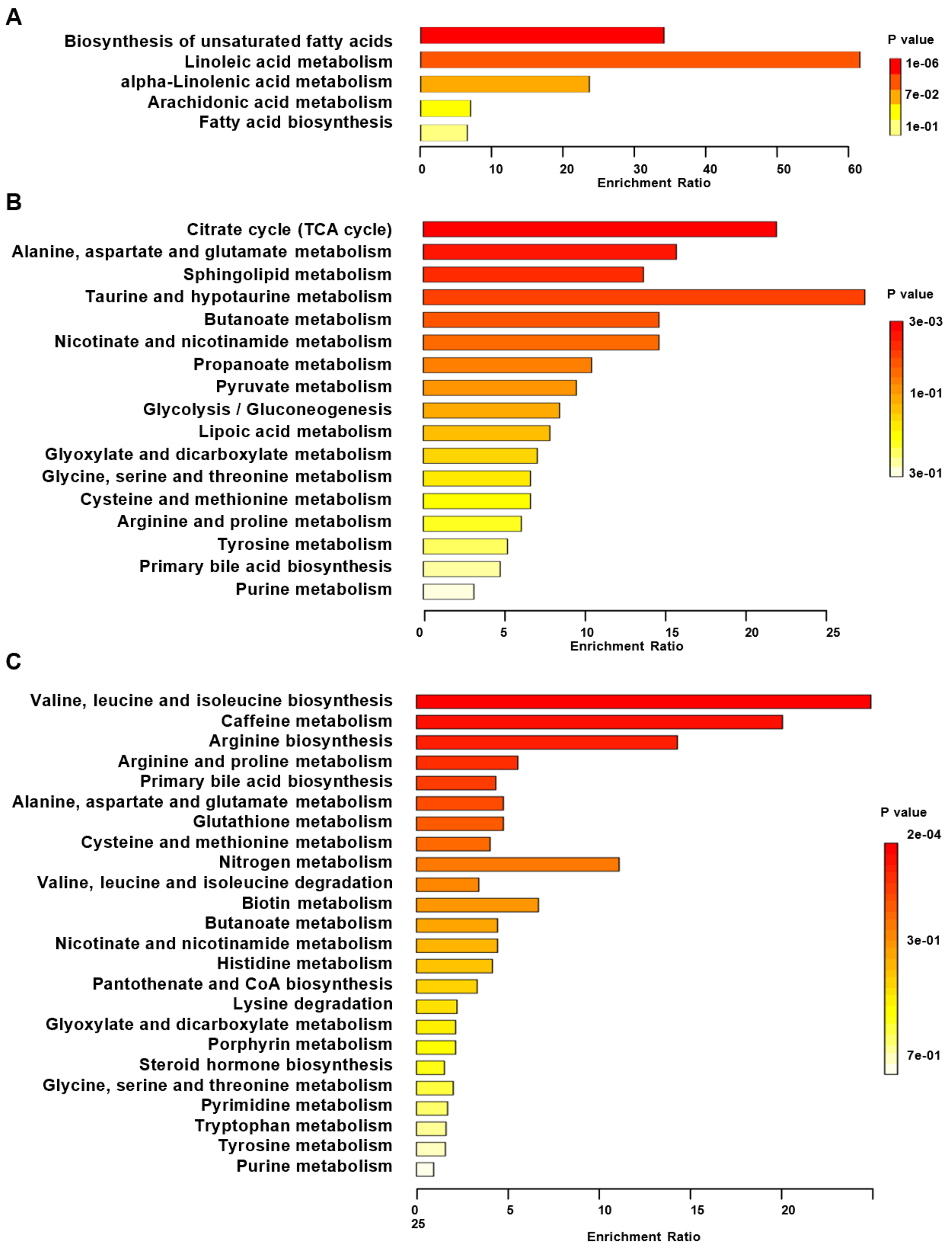

Supplementary Fig. 8 Biological functional annotation of NMF-derived metabolic modules via KEGG enrichment. (**A–C**) Enriched pathways for cluster 2 (A), cluster 4 (B), cluster 5 (C). Bar length: enrichment ratio; color scale: *P* value. Abbreviations: NMF, non-negative matrix factorization. Source data are provided as a Source Data file.

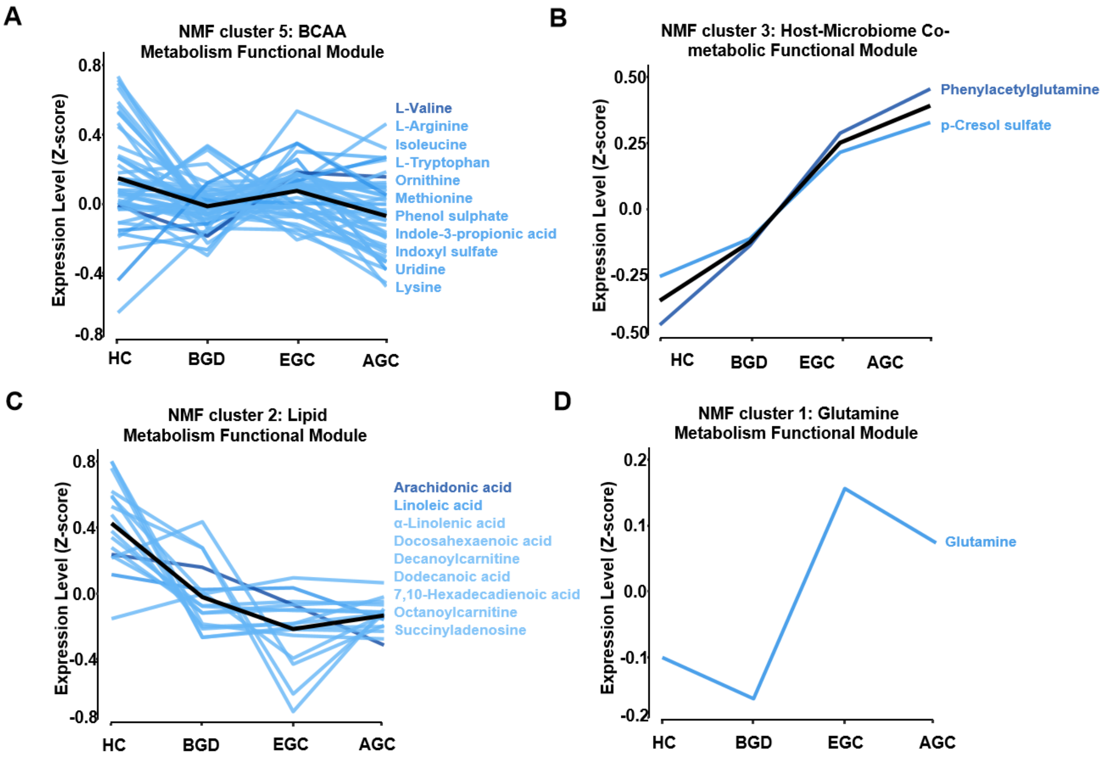

Supplementary Fig. 9 Longitudinal metabolic trajectories of NMF functional modules during gastric carcinogenesis. (A–D) Longitudinal expression (Z-score) alteration across different clinical stages. Modules: BCAA metabolism (**A**, cluster 5), host-microbiome co-metabolism (**B**, cluster 3), lipid metabolism (**C**, cluster 2), glutamine metabolism (**D**, cluster 1). Line color intensity: metabolite contribution to module (darker = higher). Bold black lines: mean trajectory per cluster. Shaded ribbons represent mean ± standard error (SE) of the scaled expression values within each cluster. Abbreviations: HC, healthy controls; BGD, benign gastric disease; EGC, early gastric cancer; AGC, advanced gastric cancer; NMF, non-negative matrix factorization. Source data are provided as a Source Data file.

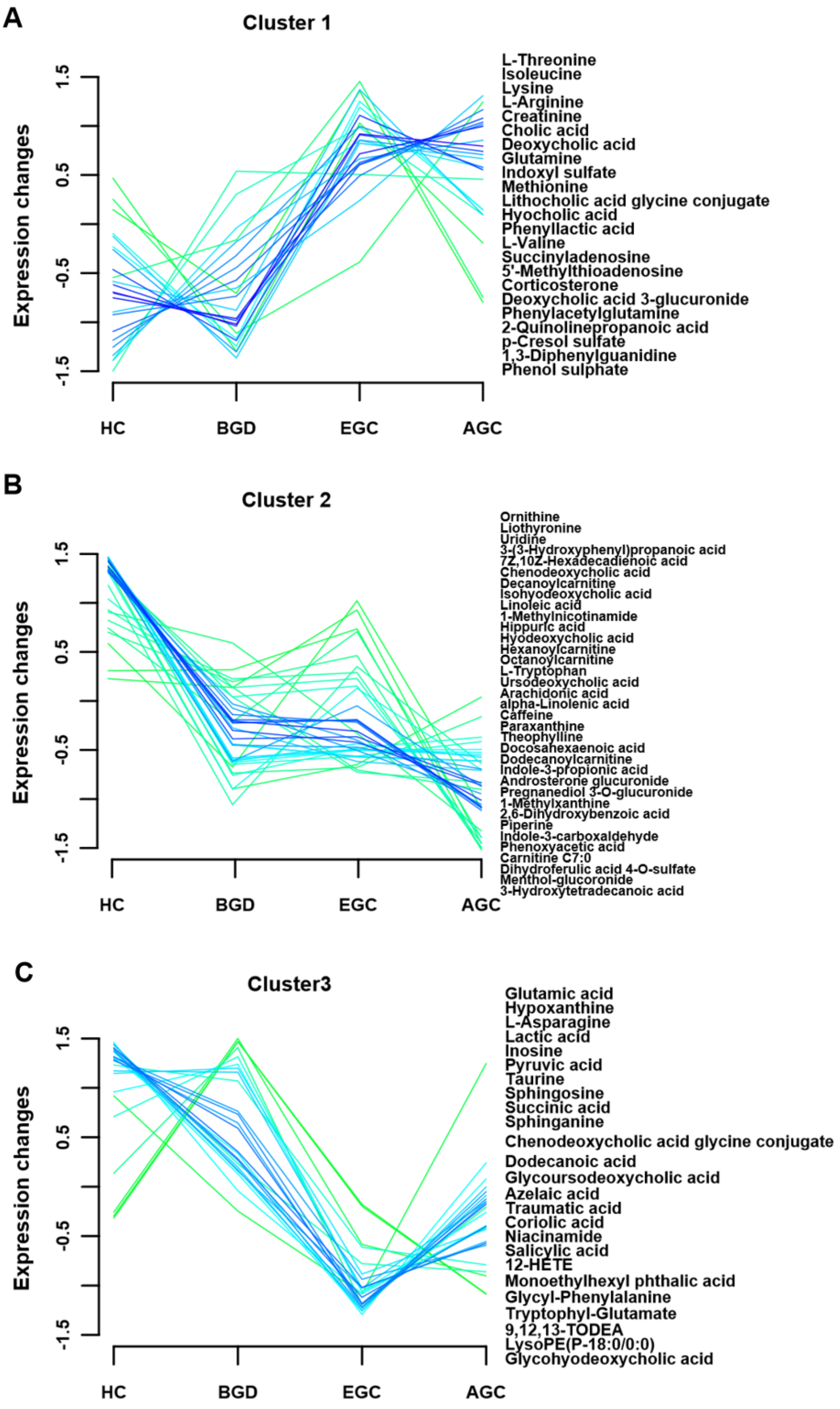

Supplementary Fig. 10 Metabolic trajectories in Mfuzz temporal clusters during gastric carcinogenesis. (**A–C**) Expression profiles (Z-score) across different clinical stages. Metabolites grouped into three temporal clusters (Cluster 1–3) via Mfuzz. Line color intensity: membership score (darker = higher). Shaded ribbons represent mean ± standard error (SE) of the scaled expression values within each cluster. Abbreviations: HC, healthy controls; BGD, benign gastric disease; EGC, early gastric cancer; AGC, advanced gastric cancer. Source data are provided as a Source Data file.

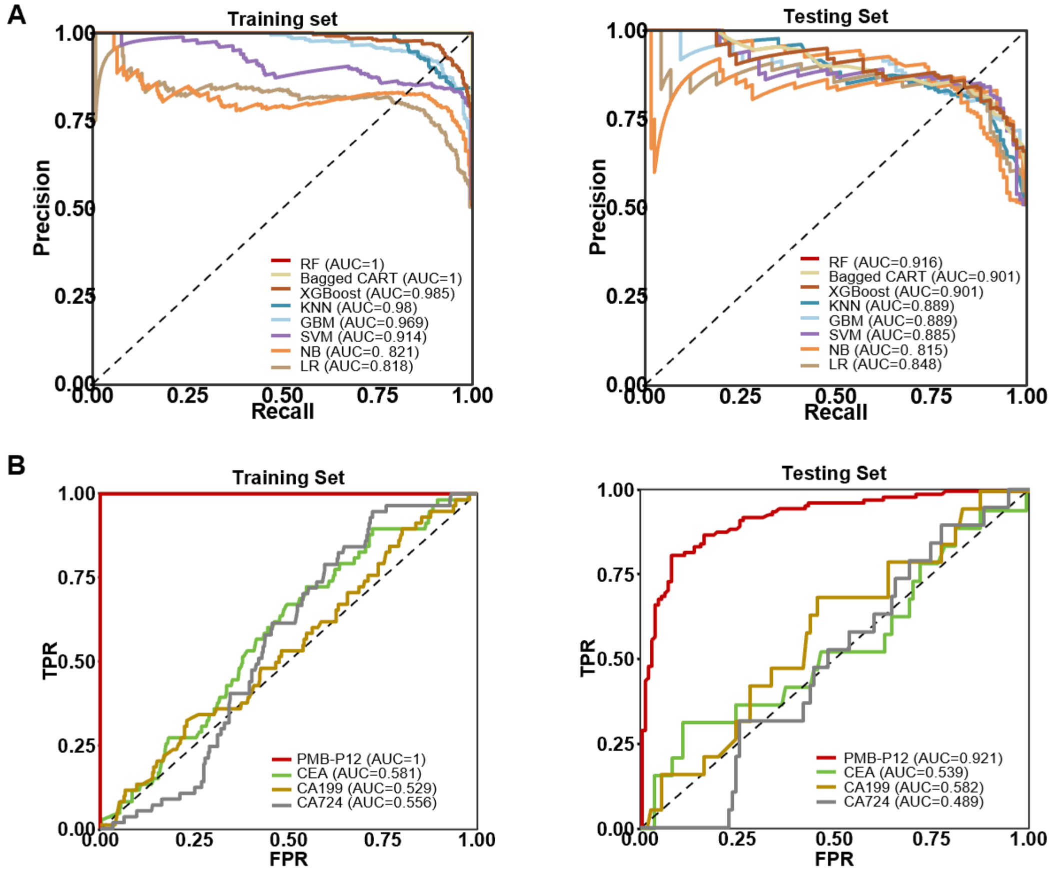

Supplementary Fig. 11 Performance evaluation of machine learning models and comparison with tumor markers. (**A**) Precision-recall curves for eight ML algorithms (PMB-P12): training set (left), testing set (right). (**B**) ROC curves: best model (random forest, RF) vs. tumor markers (CEA, CA19-9, CA72-4) in training (left) and test (right) sets. Abbreviations: ML, machine learning; GC, gastric cancer; RF, random forest; CART, classification and regression trees; GBM, gradient boosting machine; SVM, support vector machine; KNN, k-nearest neighbors; LR, logistic regression; NB, Naïve Bayes; AUC: area under the curve; PMB-P12, Plasma Metabolic Biomarker—12-metabolite panel; CEA, carcinoembryonic antigen; CA19-9, carbohydrate antigen 19-9; CA72-4, carbohydrate antigen 72-4; ROC, receiver operating characteristic curve. Source data are provided as a Source Data file.

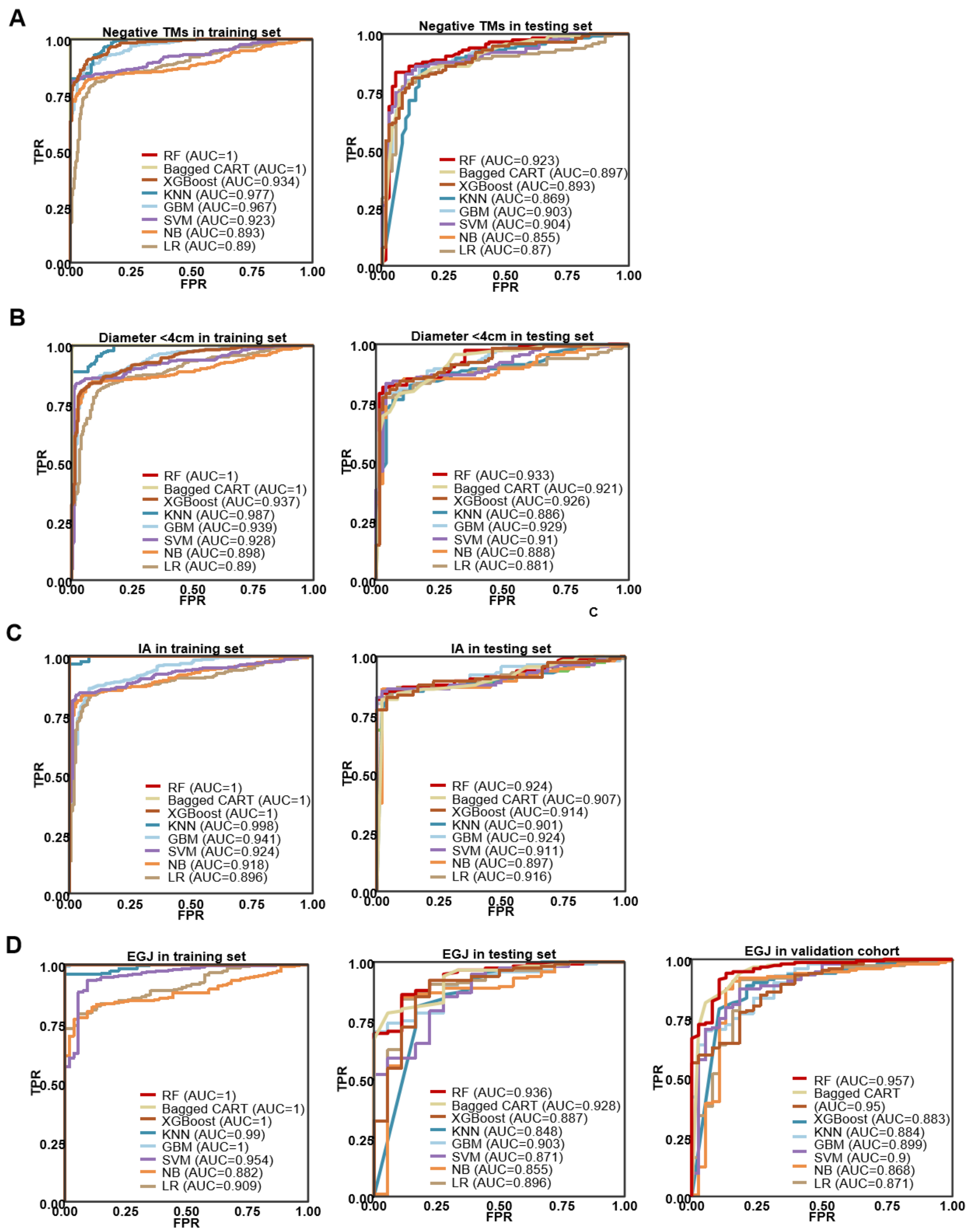

Supplementary Fig. 12 Diagnostic performance of the PMB-P12 panel in clinically challenging subgroups. ROC curves for eight ML classifiers (PMB-P12 panel) in subgroups: (**A**) TMs-negative GC (CEA/CA19-9/CA72-4 negative), (**B**) GC diameter <4 cm, (**C**) stage IA GC, (**D**) EGJ cancers. A–C: Training set (left), test set (right); D: Training (left), test (middle), validation (right). AUC values per model in legends. Sample sizes: training set n=696, testing set n=232, validation cohort n=309. Abbreviations: PMB-P12, Plasma Metabolic Biomarker—12-metabolite panel; ML, machine learning; GC, gastric cancer; EGJ, esophagogastric junction; RF, random forest; CART, classification and regression trees; GBM, gradient boosting machine; SVM, support vector machine; KNN, k-nearest neighbors; LR, logistic regression; NB, Naïve Bayes; AUC: area under the curve; ROC, receiver operating characteristic curve. Source data are provided as a Source Data file.

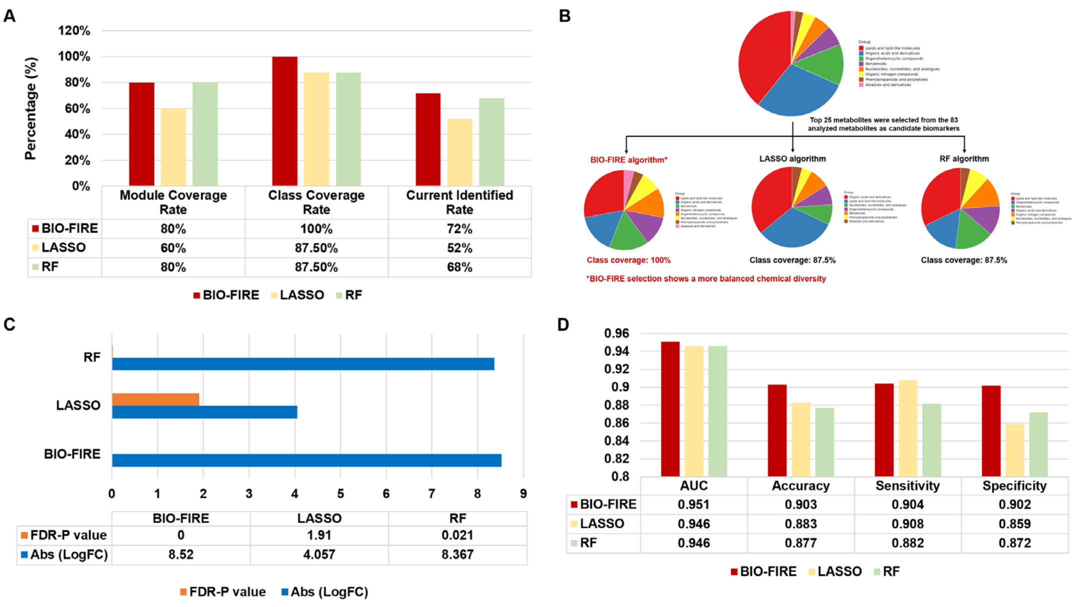

Supplementary Fig. 13 Comprehensive benchmarking of the BIO-FIRE algorithm against baseline feature selection methods. (A) Quantitative benchmarking of biological interpretability. **(B)** Changes of super class distribution before and after feature selection. **(C)** Quantitative benchmarking of statistical power. **(D)** Quantitative benchmarking of diagnostic performance. Source data are provided as a Source Data file.

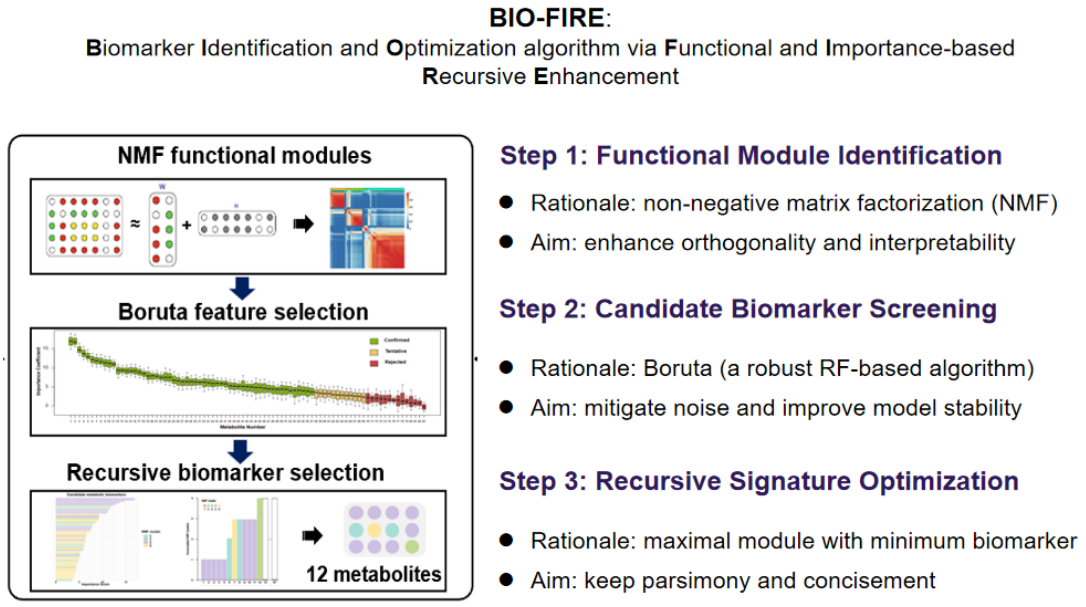

Supplementary Fig. 14 Flowchart illustrating the BIO-FIRE biomarker discovery framework. The illustration was created with a full license on BioRender.com. Abbreviations: RF, random forest.
