# Supplementary material for: Multi-phase hybrid metabolomics framework identifies clinically applicable plasma signatures for early detection of gastric cancer
Source: Nat Commun. 2026 May 13;17:6372. doi: 10.1038/s41467-026-72983-8 (PMC13376920; doi:10.1038/s41467-026-72983-8)
Supplement: Supplementary file 4 — Description of Additional Supplementary Files [file 41467_2026_72983_MOESM4_ESM.pdf]

Description of Additional Supplementary Data

Title: Supplementary Data 1

Description: Detailed list of the 84 key GC-associated metabolites identified at MSI Level 1, including metabolite name, ionization mode, retention time, m/z, and molecular formula.

Title: Supplementary Data 2

Description: KEGG pathway enrichment analysis results for the 84 key metabolites, including total pathway size, expected hits, observed hits, P-values, and pathway impact values.

Title: Supplementary Data 3

Description: Literature-curated metabolite biomarkers of gastric cancer, with their CAS numbers and status in the current study (e.g., already included, additionally detected, or not significantly altered).

Title: Supplementary Data 4

Description: Metabolite-module weight matrix and final NMF cluster assignment (Clusters 1–5) for all 83 quantified metabolites.

Title: Supplementary Data 5

Description: Comprehensive statistical summary for the 12-metabolite panel, including median abundance per group, fold changes, Kruskal-Wallis P-values, and Benjamini-Hochberg adjusted P-values for all pairwise comparisons.

Title: Supplementary Data 6

Description: Assignment of matched isotope-labelled internal standards (IS) for 56 metabolites, with regression equation, R^2, linear range, LOD, and LOQ.

Title: Supplementary Data 7

Description: Calibration curve parameters (regression equation, R^2, linear range, LOD, LOQ) for all 83 quantified metabolites.

Title: Supplementary Data 8

Description: Hyperparameter search grids and optimal values for the eight machine learning algorithms used in the study.
