# Supplementary material for: Multi-phase hybrid metabolomics framework identifies clinically applicable plasma signatures for early detection of gastric cancer
Source: Nat Commun. 2026 May 13;17:6372. doi: 10.1038/s41467-026-72983-8 (PMC13376920; doi:10.1038/s41467-026-72983-8)
Supplement: Supplementary file 2 — Transparent Peer Review file [file 41467_2026_72983_MOESM2_ESM.pdf]

nature portfolio

Peer Review File

Multi-phase hybrid metabolomics framework identifies clinically applicable plasma signatures for early detection of gastric cancer

Corresponding Author: Professor Li Min

This file contains all reviewer reports in order by version, followed by all author rebuttals in order by version.

Version 0:

Reviewer comments:

Reviewer #1

(Remarks to the Author)
In this study, Bai et al. present a multi-phase hybrid metabolomics framework, successfully identifying and validating a 12-metabolite panel with high diagnostic performance for gastric cancer. The research is thoughtfully designed, methodologically innovative, and executed with remarkable rigor, particularly in the mass spectrometry analysis and de novo biomarker discovery process. The results are compelling, demonstrating excellent diagnostic capability (AUC up to 0.951) and robust performance across multiple clinically challenging subgroups. The manuscript is logically structured, well-supported by data, and features rich graphical content, representing a high-quality piece of work. I am satisfied with both the work and its presentation and recommend only minor revisions regarding the points below:
1. In Results 2.2, it is stated that "66 of these 84 metabolites (79%) are novel identifications compared to established GC biomarkers." Please briefly specify the reference set for these "established GC biomarkers" (e.g., specific prior studies or a public database) to better contextualize the claim of novelty.
2. In Results 2.5, although the reporting of AUC with confidence intervals is excellent, the perfect AUC of 1.000 (with a CI of 1-1.000) for the RF model in the training set warrants a brief comment in the text or figure legend. A brief statement is needed to recognize the potential for overfitting, while simultaneously highlighting that the model's validation on independent datasets proves its generalizability.
3. The custom algorithm name "BIO-FIRE" is memorable. The authors are suggested to provide the rationale for this name, since it is an integral part of the algorithm's description and serves to improve transparency and readability for the scientific audience.
4. In Results 2.2, the fold change threshold is described as "FC > 1.2 or < 0.83." To enhance immediate clarity, consider rephrasing this to "|fold change| (FC) > 1.2" or "FC > 1.2 or FC < 1/1.2."
5. The abbreviation "NMF" in the legends for Figures 3 and 4 currently expands to "negative matrix factorization." This should be corrected to the standard full term, "non-negative matrix factorization."
6. The legend for Figure 4 would benefit from concise descriptions of the dominant temporal patterns for metabolites B-M (e.g., "sustained increase", "biphasic trajectory") to immediately guide reader interpretation.

Reviewer #2

(Remarks to the Author)
The authors propose a plasma-based 12-metabolite panel for gastric cancer detection. While the motivation is sound, the methodology is not reproducible, and several claims are insufficiently substantiated.

Strengths
• Scale of the dataset. The cohort includes 1,706 individuals, nearly half of whom have gastric cancer. The scale is impressive.
• Use of targeted and untargeted metabolomics. Combining both approaches for biomarker discovery strengthens coverage.

Major Concerns

• Machine-learning models lack hyperparameters. The paper states that performance was assessed across multiple algorithms—"ensemble methods (RF, bagged CART, GBM, XGBoost), a kernel method (SVM-Radial), a linear classifier (LR), an instance-based method (KNN), and a probabilistic model (NB)"—but hyperparameters and tuning procedures are not reported for any model. This prevents replication.

• Potential data leakage. Several metrics are reported as 1.0 (e.g., "AUCs ranging from 0.854 to 1.000; accuracies from 0.812 to 1.000; sensitivities from 0.872 to 1.000; specificities from 0.751 to 1.000; PPVs from 0.781 to 1.000; NPVs from 0.852 to 1.000; and F1 scores from 0.824 to 1.000"), which suggests possible leakage or overfitting. The data-splitting strategy and any preprocessing within folds should be clarified.

• Overstated generalizability and unsupported claims. The external validation cohort appears to be drawn from a similar Chinese population and likely processed in the same laboratory. Without validation at an independent site (different batch, instrument, and ethnicity), claims of "clinical generalizability" are overstated. More broadly, statements such as "Non-negative matrix factorization (NMF) significantly advances precision medicine and has strong potential for elucidating meaningful metabolic modules" makes a reader question the authors understanding of the subject. Several assertions throughout the manuscript are presented without sufficient evidence and should be substantiated.

Minor Concerns:
• Code transparency. A publicly available repository (e.g., GitHub) with all analysis code and workflows is essential for reproducibility and community uptake.
• Tool versions missing. Multiple tools are cited (e.g., NMF, Boruta, Mfuzz, caret), but the exact versions are not reported. Versioning is required for reproducibility.
• Limited transparency around BIO-FIRE. Although BIO-FIRE integrates NMF and Boruta, there is no comparison with simpler feature-selection baselines (e.g., LASSO). The "recursive coverage optimization" criterion is described qualitatively; quantitative benchmarking of biological interpretability is absent.

Reviewer #3

(Remarks to the Author)
The manuscript by Bai et al describes the combination of targeted and untargeted metabolomics for gastric cancer biomarker discovery. The manuscript addresses important limitations of "traditional" metabolomics based biomarker discovery and proposes a novel framework. The manuscript is well written and should be of interest to the broad readership of Nature Communications.

Please define "interpretability-driven algorithm" what does this exactly mean exactly?
In line 157 the authors state "1,097 key signals…were identified" is that true? Were all these signals identified and assigned unambiguously to a metabolite? If I understand correctly the authors only used molecular formula matching without MS2. This is risky and might lead to misleading conclusions. Please clarify this. The matching with standards is sound though.
What does not become entirely clear to me and should maybe be stressed a bit more, how does BIO-FIRE work what does it exactly do and why is it advantageous when compared to established methods?

In the methods section, this sentence seems flawed "For absolute-quantitative metabolomic profiling, 50 μL 473 of plasma was combined with 150 μL of the extraction solvent and vortexed for 3 474 minutes. Following centrifugation under identical condition, 170 μL of the supernatant 475 was transferred to a 96-well plate for protein precipitation." Wasn't the protein precipitated by adding the extraction solvent?
Please define, MIM-IDA-EPI
In the absolute quantification work-flow did the authors use labelled internal standards? Given several of the identified biomarkers are bile acids this is highly important. From experience in my laboratory we even know that absolute BA quantification is only possible by using individual labelled IS for each BA metabolite. Please clarify and comment. Moreover, if no labelled IS were used how solid are the so called absolute quantities?

Reviewer #4

(Remarks to the Author)
I co-reviewed this manuscript with one of the reviewers who provided the listed reports. This is part of the Nature Communications initiative to facilitate training in peer review and to provide appropriate recognition for Early Career Researchers who co-review manuscripts.

Version 1:

Reviewer comments:

Reviewer #1

(Remarks to the Author)

The authors have clearly made meaningful improvements to the manuscript based on earlier reviewer feedback. Specifically, the expanded methodological descriptions, additional explanations around the hybrid metabolomics workflow, and improved figure organization all contribute to better clarity and readability.

1. BIO-FIRE Algorithm Still Needs Clearer Consolidation

The manuscript explains the components of BIO-FIRE (NMF, Boruta, recursive optimization), but the description remains scattered across multiple sections. A short, unified paragraph that outlines:
- the sequential steps,
- inputs and outputs of each stage, and
- the stopping criteria for the recursive step

would make the workflow much easier to follow for readers attempting to understand or replicate the method.

2. Add Key Statistics for the 12 Biomarkers to Support Fig. 4b–m

While the visual progression patterns are informative, adding a small summary table (or statistics in the supplement) for each of the 12 final metabolites would make Fig. 4 more self-explanatory. Also, given that the authors have multiple different classes, multiple testing inflation might help account for the data.

Minor Changes:
1. Improving Readability:

There are multiple sentences which are difficult to read through and understand. Here are some examples:

“Conceptually, BIO-FIRE is an interpretability-driven framework designed to overcome the limitations of traditional statistics-driven methods (e.g., LASSO), which often select redundant features from dominant pathways.” - what does interpretability-driven mean?
“To validate the methodological superiority of BIO-FIRE, we benchmarked it against established baselines: LASSO and standard RF.” – was RF used as classifier or regression? How exactly?
(Remarks on code availability)

Reviewer #3

(Remarks to the Author)
The authors have responded convincingly and in great detail to my questions. I have no further remarks.

(Remarks on code availability)

Reviewer #4

(Remarks to the Author)
I co-reviewed this manuscript with one of the reviewers who provided the listed reports. This is part of the Nature Communications initiative to facilitate training in peer review and to provide appropriate recognition for Early Career Researchers who co-review manuscripts.

(Remarks on code availability)

Open Access This Peer Review File is licensed under a Creative Commons Attribution 4.0 International License, which permits use, sharing, adaptation, distribution and reproduction in any medium or format, as long as you give appropriate credit to the original author(s) and the source, provide a link to the Creative Commons license, and indicate if changes were made.
In cases where reviewers are anonymous, credit should be given to 'Anonymous Referee' and the source.
The images or other third party material in this Peer Review File are included in the article's Creative Commons license, unless indicated otherwise in a credit line to the material. If material is not included in the article's Creative Commons license and your intended use is not permitted by statutory regulation or exceeds the permitted use, you will need to obtain permission directly from the copyright holder.
To view a copy of this license, visit https://creativecommons.org/licenses/by/4.0/

Point-by-point responses to the reviewer's comments:

We would like to express our sincere gratitude to all reviewers for the comprehensive and constructive evaluation of our manuscript. We have carefully considered all comments and have performed additional analyses and text revisions to address each point raised. These revisions have significantly strengthened the rigor, clarity, and transparency of our work. Below, we provide a point-by-point response to the reviewers' comments. All modifications pertaining to the original manuscript are indicated using *italic and underlined text.*

<Comments from Reviewer #1>

In this study, Bai et al. present a multi-phase hybrid metabolomics framework, successfully identifying and validating a 12-metabolite panel with high diagnostic performance for gastric cancer. The research is thoughtfully designed, methodologically innovative, and executed with remarkable rigor, particularly in the mass spectrometry analysis and de novo biomarker discovery process. The results are compelling, demonstrating excellent diagnostic capability (AUC up to 0.951) and robust performance across multiple clinically challenging subgroups. The manuscript is logically structured, well-supported by data, and features rich graphical content, representing a high-quality piece of work. I am satisfied with both the work and its presentation and recommend only minor revisions regarding the points below.

Response: We appreciate your time and efforts in reviewing our manuscript, along with your positive and constructive comments aimed at strengthening our paper.

#Comment 1: In Results 2.2, it is stated that "66 of these 84 metabolites (79%) are novel identifications compared to established GC biomarkers." Please briefly specify the reference set for these "established GC biomarkers" (e.g., specific prior studies or a public database) to better contextualize the claim of novelty.

#Response 1: We appreciate this valuable suggestion. To rigorously define the "novelty" of our identified biomarkers, we constructed a curated literature set of gastric cancer (GC) associated metabolites through a systematic literature review. Specifically,

we searched PubMed, Embase, and ScienceDirect for GC metabolomics studies published up to July 2025. From 57 eligible original articles, we compiled a list of 243 distinct metabolites previously reported as differentially expressed in GC. By cross-referencing our 84 key metabolites against this curated list, we identified an overlap of 18 metabolites (defined as "literature reported"). Consequently, the remaining 66 metabolites were classified as "current identified" in the context of GC plasma biomarkers. The novelty assessment results of the 84 key metabolites are clearly shown in **Supplementary Table 4**.

We have added the detailed methodology for constructing this reference set in the Methods section (Page 19-20, Line 618-622) as below: *To benchmark the novelty of identified metabolites, a systematic literature review was conducted searching PubMed, Embase, and ScienceDirect (up to July 2025). A reference set of 243 "known GC-associated metabolites" was compiled from 57 published studies. Novelty was defined as metabolites absent from this cumulative list.*

Additionally, we have illustrated the detailed process of systematic review and the list of included 57 original articles in **Response Letter Figure 1**, and provided the full list of 243 metabolites in **Response Letter Table 1** (The bolded items represent the 18 overlapped metabolites).

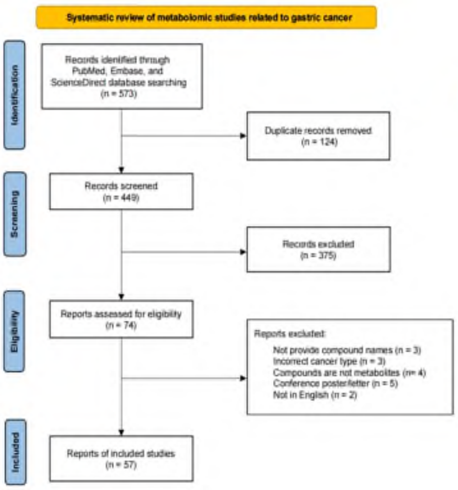

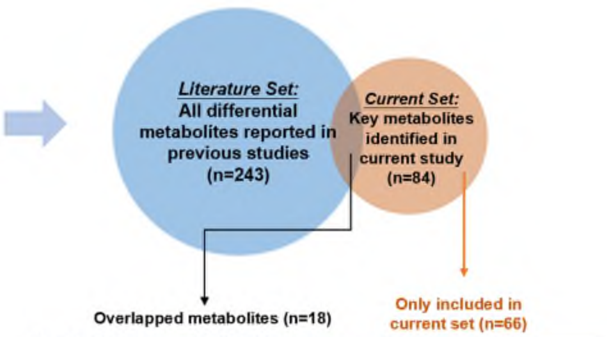

Overlapped metabolites (n=18)

No.	Metabolite name	No.	Metabolite name
1	Taurine	10	Hexanoylcarnitine
2	Azelaic acid	11	Hippuric acid
3	Hypoxanthine	12	Hyocholic acid
4	Phenylacetylglutamine	13	Inosine
5	Decanoylcarnitine	14	Methionine
6	Androsterone glucuronide	15	L-Tryptophan
7	L-Arginine	16	Succinyladenosine
8	Deoxycholic acid 3-glucuronide	17	Salicylic acid
9	Dodecanoic acid	18	Uridine

Only included in current set (n=66)

No.	Metabolite name
1	12-HETE
2	Monoethylhexyl phthalic acid
3	5'-Methylthioadenosine
4	9,12,13-TODEA
5	Glycyl-Phenylalanine
6	Niacinamide
7	Sphingosine
8	Traumatic acid
…	…
66	Valsartan

List of finally included 57 original articles for candidate GC biomarker set construction

No.	Year	Journal	Title	DOI/PMID
1	2004	Magn Reson Imaging	Ex vivo proton MR spectroscopy (1H-MRS) for evaluation of human gastric carcinoma	10.1016/j.mri.2004.01.045
2	2006	Oncol Rep	Ex vivo HR-MAS Magnetic Resonance Spectroscopy of human gastric adenocarcinomas: a comparison with healthy gastric mucosa	10.3892/or.16.3.543
3	2008	J Chromatogr B Analyt Technol	Identification of volatile organic compounds secreted from cancer tissues and bacterial cultures	10.1016/j.jchromb.2008.04.038
4	2008	Cancer Epidemiol Biomarkers	Biochemical alterations from normal mucosa to gastric cancer by ex vivo magnetic resonance spectroscopy	10.1158/1055-9965.Epi-07-2676
5	2009	Cancer Res	Quantitative metabolome profiling of colon and stomach cancer microenvironment by capillary electrophoresis time-of-flight mass spectrometry	10.1158/0008-5472.CAN-08-4806
6	2010	Mol Cell Proteomics	A combined proteomics and metabolomics profiling of gastric cardia cancer reveals characteristic dysregulations in glucose metabolism	10.1074/mcp.M110.000661
7	2010	World J Gastroenterol	Metabolomics of gastric cancer metastasis detected by gas chromatography and mass spectrometry	10.3748/wjg.v16.i46.5874
8	2010	J Toxicol Environ Health A	Toxicometabolomics of urinary biomarkers for human gastric cancer in a mouse model	10.1080/15287394.2010.511545
9	2010	Anal Bioanal Chem	Metabolomic investigation of gastric cancer tissue using gas chromatography/mass spectrometry	10.1007/s00216-009-3317-4
10	2011	Metabolomics	Metabolic features of the tumor microenvironment of gastric cancer and the link to the systemic macroenvironment	10.1007/s11306-011-0297-0
11	2011	World J Gastroenterol	Prediction of gastric cancer metastasis through urinary metabolomic investigation using GC/MS	10.3748/wjg.v17.i6.727
12	2011	PLoS One	Plasma free amino acid profiling of five types of cancer patients and its application for early detection	10.1371/journal.pone.0024143
13	2011	Oncol Rep	Tissue metabolomic fingerprinting reveals metabolic disorders associated with human gastric cancer morbidity	10.3892/or.2011.1302
14	2011	J Gastroenterol Hepatol	Metabolomic phenotype of gastric cancer and precancerous stages based on gas chromatography time-of-flight mass spectrometry	10.1111/j.1440-1746.2011.06724.x
15	2011	J Thorac Cardiovasc Surg	Metabolomics study of esophageal adenocarcinoma	10.1016/j.jtcvs.2010.08.025
16	2012	Biomed Chromatogr	Serum metabolomics as a novel diagnostic approach for gastrointestinal cancer	10.1002/bmc.1671
17	2012	Braz J Med Biol Res	Serum metabolic profiling of human gastric cancer based on gas chromatography/mass spectrometry	10.1590/s0100-879x2011007500158
18	2012	Chin Med J (Engl)	Metabolic disorders of fatty acids and fatty acid amides associated with human gastric cancer morbidity	PMID: 22490569
19	2013	Chin Med J (Engl)	Urinary metabonomics of stomach cancer assessed by rapid resolution liquid chromatography/time-of-fight mass spectrometry	PMID: 23673112
20	2014	Electrophoresis	CE-MS based on moving reaction boundary method for urinary metabolomic analysis of gastric cancer patients	10.1002/elps.201300243
21	2014	PLoS One	Quantitative measurement of organic acids in tissues from gastric cancer patients indicates increased glucose metabolism in gastric cancer	10.1371/journal.pone.0098581
22	2014	Ann Surg Oncol	Noninvasive diagnosis and evaluation of curative surgery for gastric cancer by using NMR-based metabolomic profiling	10.1245/s10434-014-3886-0
23	2015	Appl Biochem Biotechnol	Metabolomic Analysis Using Liquid Chromatography/Mass Spectrometry for Gastric Cancer	10.1007/s12010-015-1706-z
24	2015	J Gastrointest Cancer	Determination of urinary 5-hydroxyindoleacetic acid as a metabolomics in gastric cancer	10.1007/s12029-015-9700-9
25	2016	Br J Cancer	(1)H-NMR urinary metabolomic profiling for diagnosis of gastric cancer	10.1038/bjc.2015.414
26	2016	Oncotarget	A characteristic biosignature for discrimination of gastric cancer from healthy population by high throughput GC-MS analysis	10.18632/oncotarget.11754
27	2016	Biomed Chromatogr	Development of simultaneous analysis of tryptophan metabolites in serum and gastric juice - an investigation towards establishing a biomarker test for gastric	10.1002/bmc.3773
28	2016	J Chromatogr A	Comprehensive profiling of ribonucleosides modification by affinity zirconium oxide-silica composite monolithic column online solid-phase microextraction - Mass	10.1016/j.chroma.2016.07.086
29	2016	J Proteome Res	Metabolomic Analysis of Gastric Cancer Progression within the Correa's Cascade Using Ultraperformance Liquid Chromatography-Mass Spectrometry	10.1021/acs.jproteome.6b00281
30	2016	BMC Cancer	Tissue metabolic profiling of human gastric cancer assessed by (1)H NMR	10.1186/s12885-016-2356-4
31	2017	Sci Rep	Plasma sample based analysis of gastric cancer progression using targeted metabolomics	10.1038/s41598-017-17921-x
32	2017	Oncotarget	Serum metabolomic profiling of human gastric cancer and its relationship with the prognosis	10.18632/oncotarget.21314
33	2018	Int J Mol Sci	Use of Metabolomics as a Complementary Omic Approach to Implement Risk Criteria for First-Degree Relatives of Gastric Cancer Patients	10.3390/ijms19030750
34	2018	IUBMB Life	Discriminating gastric cancer and gastric ulcer using human plasma amino acid metabolic profile	10.1002/iub.1748
35	2018	BMC Cancer	Serum microRNA signatures and metabolomics have high diagnostic value in gastric cancer	10.1186/s12885-018-4343-4
36	2018	Metabolomics	(1)H NMR metabolic profiling of gastric cancer patients with lymph node metastasis	10.1007/s11306-018-1344-x
37	2018	Anal Chem	Highly Sensitive Quantification Method for Amine Submetabolome Based on AQC-Labeled-LC-Tandem-MS and Multiple Statistical Data Mining: A Potential	10.1021/acs.analchem.8b02372
38	2019	J Biophotonics	A novel urine analysis technique combining affinity chromatography with Au nanoparticle based surface enhanced Raman spectroscopy for potential applications in	10.1002/jbio.201800327
39	2019	Sci Rep	A multicentre clinical validation of AminoIndex Cancer Screening (AICS)	10.1038/s41598-019-50304-y
40	2020	Cancer Sci	Nuclear magnetic resonance-based tissue metabolomic analysis clarifies molecular mechanisms of gastric carcinogenesis	10.1111/cas.14443
41	2020	Gastric Cancer	Metabolomic profiling of gastric cancer tissues identified potential biomarkers for predicting peritoneal recurrence	10.1007/s10120-020-01065-5
42	2021	JAMA Netw Open	Identification and Validation of Plasma Metabolomic Signatures in Precancerous Gastric Lesions That Progress to Cancer	10.1001/jamanetworkopen.2021.14186
43	2021	Cancer Epidemiol Biomarkers	A Prospective Investigation of Circulating Metabolome Identifies Potential Biomarkers for Gastric Cancer Risk	10.1158/1055-9965.Epi-20-1633
44	2021	Anal Chim Acta	Across-polarity quantification method for broad metabolome coverage based on consecutive nanoconfined liquid phase nanoextraction technology: Application in	10.1016/j.aca.2021.338577
45	2021	Front Oncol	Serum Metabolic Profiling Analysis of Chronic Gastritis and Gastric Cancer by Untargeted Metabolomics	10.3389/fonc.2021.636917
46	2022	PeerJ	Targeted metabolomic profiles of serum amino acids and acylcarnitines related to gastric cancer	10.7717/peerj.14115
47	2022	Front Endocrinol (Lausanne)	Metabolomics study identified bile acids as potential biomarkers for gastric cancer: A case control study	10.3389/fendo.2022.1039786
48	2022	Clin Cancer Res	Spatial Metabolomics Identifies Distinct Tumor-Specific Subtypes in Gastric Cancer Patients	10.1158/1078-0432.CCR-21-4383
49	2022	Sci Rep	Metabolite biomarker discovery for human gastric cancer using dried blood spot mass spectrometry metabolomic approach	10.1038/s41598-022-19061-3
50	2022	J Transl Med	Microbiota and metabolites alterations in proximal and distal gastric cancer patients	10.1186/s12967-022-03650-x
51	2022	Front Genet	Lipid metabolism characterization in gastric cancer identifies signatures to predict prognostic and therapeutic responses	10.3389/fgene.2022.959170
52	2023	Digestion	Targeted Metabolomic Profiling of Plasma Samples in Gastric Cancer by Liquid Chromatography-Mass Spectrometry	10.1159/000526864
53	2023	Metabolomics	Circulating metabolites as potential biomarkers for the early detection and prognosis surveillance of gastrointestinal cancers	10.1007/s11306-023-02002-0
54	2023	Gut	Efficient plasma metabolic fingerprinting as a novel tool for diagnosis and prognosis of gastric cancer: a large-scale, multicentre study	10.1136/gutjnl-2023-330045
55	2023	J Proteome Res	Metabolomics Reveals Novel Serum Metabolic Signatures in Gastric Cancer by a Mass Spectrometry Platform	10.1021/acs.jproteome.2c00295
56	2024	EMBO Molecular Medicine	Diagnosis and prognosis prediction of gastric cancer by high-performance serum lipidome fingerprints	10.1038/s44321-024-00169-0
57	2024	Nat Commun	Metabolomic machine learning predictor for diagnosis and prognosis of gastric cancer	10.1038/s41467-024-46043-y

Response Letter Figure 1. Flowchart of the process for evaluating metabolites novelty

Respond Letter Table 1. Summary of the 243 known GC-associated metabolites list.

No.	Metabolite Name	No.	Metabolite Name	No.	Metabolite Name	No.	Metabolite Name	No.	Metabolite Name	No.	Metabolite Name
1	Succinylacetone	46	L-Cysteine	91	Tryptophol	136	N-Acetylaspartic acid	181	Allantoic acid	226	O-Acetylcarnitine
2	4-Acetamidobutanoic acid	47	L-Kynurenine	92	gamma-Glu-Cys	137	N-Acetylglucosamine	182	Adenosine	227	Malonylcarnitine
3	Succinic acid	48	Kynurenic acid	93	Suberic acid	138	N1-Acetylspermine	183	Acetamide	228	S-Adenosylmethionine (SAM)
4	L-Asparagine	**49**	**Inosine**	94	Cer(t42:0)	139	N,N-Diacetylspermine	184	2,3-Dihydroxyisobutyric acid	229	Isovalerylcarnitine
5	Glutamine	50	Indolelactic acid	95	Testosterone	140	Methylmalonic acid	185	Oleic acid	230	3-Hydroxybutyrylcarnitine
6	1-Methylnicotinamide	**51**	**Hippuric acid**	96	Taurolithocholic acid	141	Methylguanidine	186	Oleamide	**231**	**Hexanoylcarnitine**
7	Ornithine	52	Glycolic acid	97	Temurin	142	Methylamine	187	7-Methylguanosine	232	Hexadecadienylcarnitine
8	Pyruvic acid	53	Gluconic acid	98	Propanoic acid	143	Melatonin	188	5-Methyluridine	233	Hexadecasphinganine
9	Linoleic acid	54	Fructose	99	Tryptamine	144	Megestrol	189	5-Methoxytryptamine	234	FAHFA (18:1/18:0)
10	Arachidonic acid	55	Anthranilic acid	100	Serotonin	145	Mannitol	190	5-HIAA	235	PE(Me2_36:2)
11	Sarcosine	56	Xanthosine	101	Myristic acid	146	Levulinic acid	191	5-Aminolevulinic acid	**236**	**Decanoylcarnitine**
12	Creatinine	57	Uracil	102	Urea	147	Isocitric acid	192	4-Hydroxybenzoic acid	237	Decadienoylcarnitine
13	Choline	58	TMAO	103	Squalene	148	IMP	193	3-Methyluridine	238	beta-Ureidoisobutyric acid
14	Lactic acid	59	beta-Alanine	104	Linoleamide	149	Indole-3-acetamide	194	3-Methylindole	239	1-Palmitoylglycerol
15	alpha-Linolenic acid	**60**	**Uridine**	105	Salicyluric acid	150	Hydroxyproline	195	Indole-3-acetic acid	240	Plasmologen PE
**16**	**Hyocholic acid**	61	Sucrose	106	gamma-Linolenic acid	151	Homocysteine	196	3-Hydroxykynurenine	241	N-Acetyl-GABA
17	L-Valine	62	Stearic acid	107	trans-3-Hydroxy-L-proline	152	Pimelic acid	197	3-Hydroxyisobutyric acid	242	N-Formylkynurenine
18	Tyrosine	63	Spermidine	108	N-Acetylserotonin	153	Harzianopyridone	198	3-Hydroxybutyric acid	243	S-Adenosylhomocysteine (SAH)
19	Alanine	64	Uric acid	109	Thymine	154	Guanosine	199	2-Ketobutyric acid		
**20**	**L-Tryptophan**	65	Threonic acid	110	Xanthine	155	Guanine	200	2-Hydroxyisobutyric acid		
**21**	**Methionine**	66	Pyroglutamic acid	111	Ribulose	156	Glycocholic acid	201	2-Hydroxybutyric acid		
22	Isoleucine	67	Xanthurenic Acid	112	Quinic acid	157	Glycerol	202	2-Furoic acid		
23	Histidine	**68**	**Deoxycholic acid 3-glucuronide**	113	Quinolinic acid	158	Glutathione	203	2-Butanone		
24	L-Threonine	69	myo-Inositol	114	Pyrophosphate	159	Glutaric acid	204	2-Aminophenol		
25	Proline	70	Malic acid	115	Pyrimidine	160	Fructose 6-Phosphate	205	1-Propanol		
**26**	**L-Arginine**	71	Glyceraldehyde	116	SDMA	161	Ethanol	206	1-Methylinosine		
27	Phenylalanine	72	GABA	117	Xylonic acid	162	D-Tagatose	207	1-Methyladenosine		
28	Lysine	73	L-Carnitine	**118**	**Salicylic acid**	163	D-Ribose	208	NMNH		
29	Leucine	74	Dimethylamine	119	Pseudouridine	**164**	**Dodecanoic acid**	209	Decatrienoylcarnitine		
30	Glutamic acid	75	Cystine	120	Pipecolic acid	165	Dimethylglycine	210	Acetylcarnitine		
31	Citrulline	76	Creatine	121	Phosphoric acid	166	Dihydrouracil	211	Adipoylcarnitine		
**32**	**Taurine**	77	Betaine	122	Phosphocreatine	167	Dihydronicotinamide	212	Adrenic acid		
**33**	**Androsterone glucuronide**	78	Adipic acid	123	Phomalone	168	Deoxyribose	213	Octadecenoylcarnitine		
34	Cholesterol	79	Adenine	124	p-Cresol	169	Cytidine	214	Phosphocholine		
35	alpha-Ketoglutaric acid	**80**	**Succinyladenosine**	125	Pantothenic acid	170	Cystathionine	215	TAG(66:3)		
36	alpha-D-Glucose	81	Acetone	126	o-Tyrosine	171	cis-Vaccenic acid	216	Sphingosine (d16:1)		
37	Palmitic acid	82	Acetoacetic acid	127	Orotidine	172	cis-Aconitic acid	217	LysoPC(18:0)		
**38**	**Hypoxanthine**	83	5-Hydroxytryptophan	128	Orotic acid	173	Chlorogenic acid	218	LysoPC(16:0)		
39	Formic acid	84	4-Hydroxyphenylacetic acid	129	Octanoic acid	174	Carnosine	219	Propionylcarnitine		
40	L-Aspartic acid	85	3-Hydroxyphenylacetic acid	130	N-Methylhydantoin	175	Cadaverine	220	PG(36:2)		
41	Acetic acid	86	3-Aminoisobutyric acid	131	1-Methylguanosine	176	Crotonic acid	221	PE(38:4)		
42	Sebacic acid	87	2-Aminobutyric acid	132	N6-Methyladenosine	177	Butyric acid	222	PC(42:3)		
43	Putrescine	88	trans-Aconitic acid	133	Nicotinic acid	**178**	**Azelaic acid**	223	PC(O-34:1)		
44	Phenylacetylglycine	89	gamma-Tocopherol	134	NADPH	179	Ascorbic acid	224	PC(32:0)		
**45**	**Phenylacetylglutamine**	90	Valeric acid	135	N-Acetylornithine	180	Apolipoprotein A1	225	OAHFA 27:0		

#Comment 2: In Results 2.5, although the reporting of AUC with confidence intervals is excellent, the perfect AUC of 1.000 (with a CI of 1-1.000) for the RF model in the training set warrants a brief comment in the text or figure legend. A brief statement is needed to recognize the potential for overfitting, while simultaneously highlighting that the model's validation on independent datasets proves its generalizability.

#Response 2: We fully agree with your point. As an ensemble algorithm, Random Forest (RF) can indeed achieve excellent classification performance (AUC = 1.000) on training sets with high-dimensional features, which suggests a potential risk of overfitting. However, the true measure of a model's performance lies in its generalizability to independent data. In our study, although the AUC of training set was 1.000, the model maintained a high level of performance on the testing set (AUC = 0.921), which aligns with the expected reasonable decline from training to testing phase. More importantly, the model demonstrated consistently strong diagnostic performance on a completely independent validation set, with no substantial drop in effectiveness. This confirms that the model captures stable, biologically meaningful patterns rather than random noise in the training data.

To enhance the rigor of the manuscript, we have added a discussion of this issue in the relevant section (Page 13, Line 412-420): *A noteworthy phenomenon is that RF achieved excellent discrimination (AUC=1.000) on the training set, which indicates a potential overfitting risk. Nevertheless, the algorithm maintained high diagnostic accuracy in both the internal testing set and a completely independent validation cohort. The slightly higher performance in the validation set may be attributed to the focused quantification of the final 12-marker panel, which likely reduced analytical noise compared to the broader profiling phase. This robust external performance substantiates the model's potential for clinical application, indicating that its strong predictive capability stems from likely capturing reproducible biological patterns rather than cohort-specific noise.*

#Comment 3: The custom algorithm name "BIO-FIRE" is memorable. The authors are suggested to provide the rationale for this name, since it is an integral part of the

algorithm's description and serves to improve transparency and readability for the scientific audience.

#Response 3: Thank you for the positive comment. The name BIO-FIRE was constructed to strictly reflect the algorithmic logic and scientific objectives of our framework:

BIO: **Bio**marker **I**dentification and **O**ptimization.

F: **F**unctional module-guided clustering (via NMF).

I: **I**mportance-based screening (via Boruta).

R: **R**ecursive Enhancement (iterative panel optimization).

E: **E**nhancement (representing the overall optimization process).

The acronym encapsulates the core innovation of our method: shifting from a purely statistical selection to a biologically grounded, recursive optimization process. We have updated the manuscript to clearly define each component of the acronym upon its first mention in introduction session (Page 4, Line 114-117): *We named this algorithm BIO-FIRE (Biomarker Identification and Optimization via Functional and Importance-based Recursive Enhancement) to reflect its integration of functional clustering, statistical importance, and iterative optimization during biomarker selection.*

Furthermore, in response to concerns raised by other reviewers, we have supplemented the manuscript with a quantitative and qualitative comparative analysis between the BIO-FIRE algorithm and two baseline algorithms (LASSO and standard Random Forest). The main results are shown in **Supplementary Figure 13**. Generally, these results demonstrate that BIO-FIRE outperforms conventional baselines not only in diagnostic accuracy but also in generating a biologically interpretable, diverse, and novel biomarker panel. For detailed benchmarking process and interpretation of results, please see our responses to *Minor Comment 3 of Reviewer 2*. Corresponding description has been added in the last part of Results session (Page 11, Line 326-339).

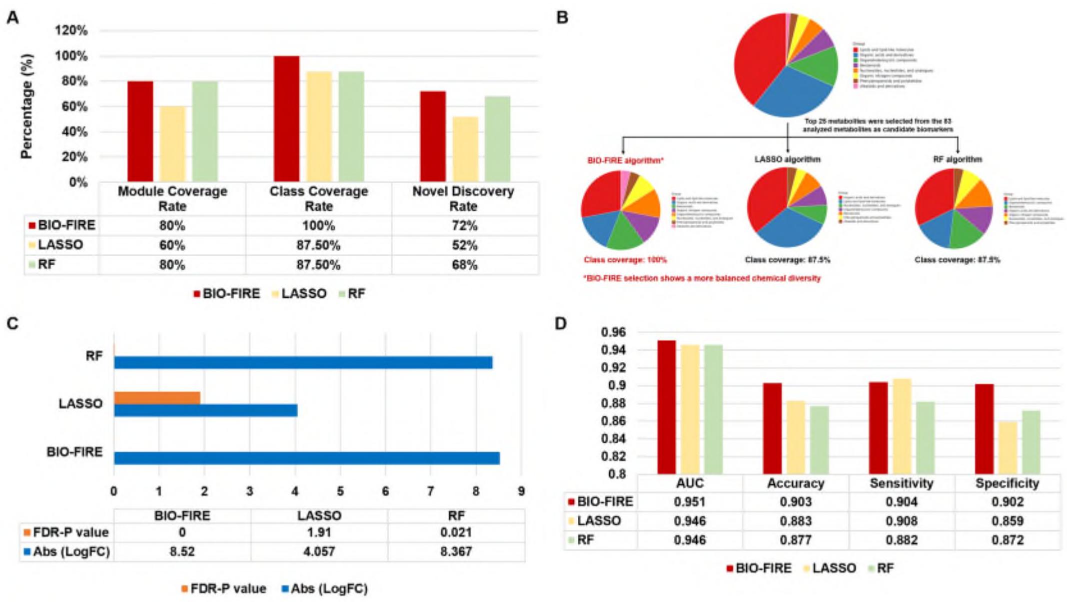

Supplementary Fig. 13 Comprehensive benchmarking of the BIO-FIRE algorithm against baseline feature selection methods. (A) Quantitative benchmarking of biological interpretability. **(B)** Changes of super class distribution before and after feature selection. **(C)** Quantitative benchmarking of statistical power. **(D)** Quantitative benchmarking of diagnostic performance.

#Comment 4: In Results 2.2, the fold change threshold is described as "FC > 1.2 or < 0.83." To enhance immediate clarity, consider rephrasing this to "|fold change| (FC) > 1.2" or "FC > 1.2 or FC < 1/1.2."

#Response 4: Thanks for your comments. We have corrected the fold change description to " FC > 1.2 or FC < 1/1.2" as suggested at all the places where it has appeared in the original text.

#Comment 5: The abbreviation "NMF" in the legends for Figures 3 and 4 currently expands to "negative matrix factorization." This should be corrected to the standard full term, "non-negative matrix factorization."

#Response 5: Thanks for your comments. We have corrected "negative matrix factorization" to " non-negative matrix factorization " in all figure legends.

#Comment 6: The legend for Figure 4 would benefit from concise descriptions of the dominant temporal patterns for metabolites B-M (e.g., "sustained increase", "biphasic

trajectory") to immediately guide reader interpretation.

#Response 6: Thanks for your comments. We have updated the legend for Figure 4 to include concise descriptions of temporal patterns: *The metabolites exhibit distinct temporal patterns: **b-c** display a sustained monotonic increase with disease progression, while **d-m** follow a biphasic trajectory characterized by a specific depletion in EGC followed by an elevation in AGC (down-up trend).*

<Comments from Reviewer #2>

The authors propose a plasma-based 12-metabolite panel for gastric cancer detection. While the motivation is sound, the methodology is not reproducible, and several claims are insufficiently substantiated.

Strengths

• Scale of the dataset. The cohort includes 1,706 individuals, nearly half of whom have gastric cancer. The scale is impressive.

• Use of targeted and untargeted metabolomics. Combining both approaches for biomarker discovery strengthens coverage.

Major Concerns

#Comment 1: Machine-learning models lack hyperparameters. The paper states that performance was assessed across multiple algorithms—"ensemble methods (RF, bagged CART, GBM, XGBoost), a kernel method (SVM-Radial), a linear classifier (LR), an instance-based method (KNN), and a probabilistic model (NB)"—but hyperparameters and tuning procedures are not reported for any model. This prevents replication.

#Response 1: Thank you for raising this important point regarding hyperparameter reporting. We sincerely apologize for the oversight in our original manuscript. To ensure full transparency and reproducibility, we have taken the following comprehensive actions:

First, we have added a detailed table below **(Supplementary Table 12)** listing both the grid search space and the optimal hyperparameters selected for each of the eight machine learning algorithms (RF, Bagged CART, GBM, XGBoost, SVM-Radial, LR, KNN, and NB).

Supplementary Table 12. Hyperparameter search grids and optimal values for the machine learning models.

Algorithm	Package (R)	Hyperparameters	Search Range / Grid	Optimal Value (Final Model)	Note / Description
RF	caret (method='rf')	mtry (No. of variables sampled)	Tested mtry values: 2, 7, 12	2	Tuned via 10-fold cross-validation
		ntree (No. of trees)	Fixed	500	Default setting
		nodesize	Fixed	1 (Default)	Default (Minimum size of terminal nodes)
		maxnodes	Default	-	Trees grew to maximum purity
Bagged CART	*caret (method = 'treebag')*	nbagg (Number of bootstrap replications)	Fixed	25	Default setting in caret/ipred
		cp (Complexity Parameter)	Default	-	Standard CART pruning (no tuning)
		Tuning Parameters	None	None	No hyperparameters are tunable for this method in caret
**GBM**	caret (method = 'gbm')	n.trees (Number of iterations)	50, 100, 150	100	Number of trees to fit
		interaction.depth (Max tree depth)	1, 2, 3	3	Controls complexity of variable interactions
		shrinkage (Learning rate)	Fixed at 0.1	0.1	Step size reduction
		n.minobsinnode (Min observations in node)	Fixed at 10	10	Minimum number of observations in terminal nodes
**XGBoost**	caret (method = 'xgbTree')	nrounds (Iterations)	50, 100, 150	50	Number of boosting rounds
		max_depth	1, 2, 3	2	Tree depth
		eta (Learning rate)	0.3, 0.4	0.3	Shrinkage
		gamma	Fixed at 0	0	Minimum loss reduction
		colsample_bytree	0.6, 0.8	0.8	Subsample ratio of columns
		min_child_weight	Fixed at 1	1	Minimum sum of instance weight
		subsample	0.5, 0.75, 1.0	1	Subsample ratio of rows
**SVM**	caret (method = 'svmRadial')	C (Cost)	0.25, 0.50, 1.00	1	Penalty for misclassification
		sigma (Kernel parameter)	Fixed	0.221435	Estimated from data (sigest)
**LR**	caret (method = 'glm')	Tuning Parameters	None	None	Standard GLM (generalized linear model) with no tunable hyperparameters
**KNN**	caret (method = 'kknn')	kmax (Max neighbors)	5, 7, 9	9	Maximum number of neighbors to consider
		distance (Minkowski distance)	2	2	Fixed (Euclidean distance)
		kernel (Weighting function)	Optimal	Optimal	Fixed (Uses an optimal weighting kernel)
NB	caret (method = 'nb')	usekernel (Kernel density estimation)	TRUE, FALSE	TRUE	Whether to use kernel density estimation
		fL (Laplace correction)	Fixed at 0	0	Laplace smoothing parameter
		adjust (Bandwidth adjustment)	Fixed at 1	1	Bandwidth adjustment for density estimation

Note: All models were trained using the caret package in R with 10-fold cross-validation. For XGBoost, a grid search of 108 combinations (2 eta × 3 max_depth × 2 colsample × 3 subsample × 3 nrounds) was performed to identify the optimal settings. Abbreviations: RF, random forest; CART, classification and regression trees; GBM, gradient boosting machine; SVM, support vector machine; KNN, k-nearest neighbors; LR, logistic regression; NB, Naïve Bayes.

Second, the corresponding methodological description has been added to the main text (Page 21, Line 666-672): *For hyperparameter tuning, a grid search was implemented using the caret package (version 7.0.1) in R. The tuning process employed 10-fold cross-validation, with the optimal hyperparameter combination for each algorithm being selected based on the highest average classification accuracy across the cross-validation folds. The complete hyperparameter search grids and the final selected values for all eight algorithms are detailed in Supplementary Table 12.*

Finally, to facilitate complete replication, we have uploaded all model training codes (including the random seed settings and the exact caret tuning loops implemented with the caret package) to our public GitHub repository (https://github.com/liyibai123/GC_Metabolomics.git). All analyses can be reproduced using the provided code and the described hyperparameter configurations.

#Comment 2: Potential data leakage. Several metrics are reported as 1.0 (e.g., "AUCs ranging from 0.854 to 1.000; accuracies from 0.812 to 1.000; sensitivities from 0.872 to 1.000; specificities from 0.751 to 1.000; PPVs from 0.781 to 1.000; NPVs from 0.852 to 1.000; and F1 scores from 0.824 to 1.000"), which suggests possible leakage or overfitting. The data-splitting strategy and any preprocessing within folds should be clarified.

#Response 2: Thanks for this critical scrutiny regarding potential data leakage and overfitting. We have carefully re-examined our pipeline and confirm that **no data leakage occurred during the model training and evaluation phases**. The excellent metrics observed in the training set primarily reflect the inherent risk of overfitting in ensemble methods like Random Forest (RF), not leakage. We provide detailed clarifications below.

1. Clarification on " Excellent Metrics": Overfitting vs. Leakage

The excellent performance metrics (e.g., AUC=1.000) cited by the reviewer were obtained **exclusively from the training set.** As a powerful ensemble algorithm, RF can achieve excellent discrimination on high-dimensional training data, indicating a potential overfitting risk. However, the true test of a model lies in its performance on

independent data.

In our study, although training AUC reached 1.000, the model maintained high performance on the strictly held-out testing set (AUC=0.921)—a decline consistent with expected generalization. Most importantly, the model demonstrated robust efficacy in a **completely independent external validation cohort**. This sustained performance confirms that the model captures stable biological patterns rather than memorizing training noise.

To enhance the rigor of the manuscript, we have added the following clarification in the Discussion (Page 13, Line 412-420): *A noteworthy phenomenon is that RF achieved excellent discrimination (AUC=1.000) on the training set, which indicates a potential overfitting risk. Nevertheless, the algorithm maintained high diagnostic accuracy in both the internal testing set and a completely independent validation cohort. The slightly higher performance in the validation set may be attributed to the focused quantification of the final 12-marker panel, which likely reduced analytical noise compared to the broader profiling phase. This robust external performance substantiates the model's potential for clinical application, indicating that its strong predictive capability stems from likely capturing reproducible biological patterns rather than cohort-specific noise.*

2. Data-Splitting and Preprocessing Strategy

To address the specific concern of data leakage, we clarify our workflow as follows.

2.1 Data-Splitting

Our modeling cohort (N=928) was split once into a training set (75%) and an internal testing set (25%) using stratified random sampling (createDataPartition in R, seed=672). All model training and hyperparameter tuning (via 10-fold cross-validation) were confined to the training set. The internal testing set was strictly held out for final evaluation only. The external validation cohort (N=309) was completely blinded to both feature selection and model development.

2.2 Preprocessing

We implemented a three-stage preprocessing protocol designed to prevent leakage:

Stage 1 (Pre-split, leakage-free preprocessing): Metabolite filtering (80% rule) and

missing-value imputation (half-minimum) were applied before any splitting. These steps use fixed thresholds or cohort-wide constants and do not introduce leakage.

Stage 2 (Normalization for feature selection): Row-sum normalization was performed on the entire modeling cohort solely for the purpose of NMF clustering and Boruta-based feature screening. This step is sample-wise (each sample divided by its own total) and does not transfer information between samples.

Stage 3 (Modeling based on original data and fixed feature): The final model was trained and validated using absolute concentrations (ng/mL) of the selected biomarkers, without any further normalization or scaling within cross-validation folds. Because the features were fixed and the data were on a comparable physical scale, no fold-dependent preprocessing was required, eliminating potential source of leakage.

To enhance the transparency, we have added a dedicated subsection titled "**Data Splitting and Preprocessing**" in the Methods section, where these procedures are now clearly described. Please see the details on Page 18-19, Line 566-587.

3. Transparency on Feature-Selection Timing

We explicitly acknowledge that the BIO-FIRE feature selection was performed on the entire modeling cohort (N=928) before train-test splitting. This approach was necessary to obtain stable metabolic modules via unsupervised learning such as NMF. We recognize that this could introduce an optimistic bias in the internal testing set. To overcome this potential bias, we validated the model on a fully independent external validation cohort that was never used in feature selection or model training. The high performance in this cohort provides strong evidence that the selected biomarkers capture generalizable biological signals, not cohort-specific artifacts.

We have added the following clarification in the Method session to clearly state the feature-selection timing and its rationale (Page 20, Line 629-634): *The BIO-FIRE algorithm integrates NMF and Boruta feature selection within a recursive workflow to generate biologically interpretable biomarker signatures. The process was applied to the entire modeling cohort (N=928) to facilitate the identification of stable and biologically representative metabolic modules. The subsequent model training and validation were then performed on the split datasets and the independent external*

cohort, respectively.

4. Enhancements for Reproducibility

All analysis code (including data splitting, preprocessing, and model training) is publicly available on GitHub (https://github.com/liyibai123/GC_Metabolomics.git). Hyperparameter search grids and optimal values for all eight algorithms are provided in **Supplementary Table 12**.

In summary, we have: 1) confirmed that **no data leakage occurred** in model training and evaluation; 2) clarified the **data-splitting and preprocessing protocols** in the Methods; 3) acknowledged and mitigated the potential **bias from feature-selection timing** via rigorous external validation; 4) added transparent discussion and released all code to ensure **full reproducibility.** We believe these clarifications could address your concerns. Thank you again for the insightful comments that have strengthened our manuscript.

#Comment 3: Overstated generalizability and unsupported claims. The external validation cohort appears to be drawn from a similar Chinese population and likely processed in the same laboratory. Without validation at an independent site (different batch, instrument, and ethnicity), claims of "clinical generalizability" are overstated. More broadly, statements such as "Non-negative matrix factorization (NMF) significantly advances precision medicine and has strong potential for elucidating meaningful metabolic modules" makes a reader question the authors understanding of the subject. Several assertions throughout the manuscript are presented without sufficient evidence and should be substantiated.

#Response 3: We sincerely appreciate your constructive critique regarding the scope of our claims. We fully agree that rigorous validation across diverse populations and platforms is essential for establishing true clinical generalizability. In response, we have tempered our conclusions and refined the manuscript to more precisely reflect the evidence.

1. Clarification on Population and Geographic Scope

We acknowledge that our study is currently limited to Chinese population. This

focus is partly driven by the epidemiology of gastric cancer (GC), as East Asia—and China in particular—shoulders a disproportionate burden, accounting for over 40% of global GC cases[1]. Furthermore, while we lack immediate access to multi-ethnic cohorts, we maximized the diversity within our available resources by recruiting participants from four distinct medical centers across Northern and Southern China. These regions exhibit significant differences in dietary habits (e.g., wheat-based vs. rice-based diets) and lifestyle factors. The consistent performance of our model across these geographically distinct cohorts supports its robustness within the Chinese population, addressing a critical public health priority.

We have revised all instances of "clinical generalizability" to more specific terms such as "robustness in independent cohorts" or "potential for clinical application." We also explicitly added the need for cross-ethnic validation in the Limitations section (Page 14, Line 443-447): *First, while our study demonstrates robust diagnostic performance across independent cohorts from diverse geographical regions within China, we acknowledge that the ethnically homogeneous study population limits immediate global extrapolation. Future validation in multi-ethnic international cohorts is essential to establish its broader clinical applicability.*

2. Addressing Batch and Instrument Variability

You are correct that samples were processed in a centralized laboratory to minimize technical variance during this discovery and initial validation phase. However, a key methodological distinction of our study is the transition from relative quantification to absolute quantification (ng/mL) using standard curves and matched internal standards.

Unlike peak areas used in untargeted metabolomics, absolute concentration is a physical quantity that is theoretically platform-independent. By building our model on absolute concentrations, we have laid the necessary groundwork for future cross-laboratory reproducibility and clinical translation, even though multi-site instrument validation was not performed in this specific study.

3. Revision of Overstated Statements

Regarding the role of NMF, our original statement was based on recent literature highlighting NMF as a promising computational approach for precision oncology[2].

However, we fully agree that in the specific context of this manuscript, the phrasing should be more precise. We have revised the text to as follows (Page 4, Line 96-99): *Non-negative matrix factorization (NMF), a machine learning technique known for its ability to generate interpretable, additive models through low-rank approximation, has emerged as a powerful tool for advancing precision medicine.*

Furthermore, we have carefully reviewed the entire manuscript to ensure that all tools are introduced accurately to describe their specific contribution to our study. Assertions regarding the performance and potential of our biomarker panel are now framed with greater caution and nuance.

4. Substantiating Assertions with Data

We have carefully reviewed the manuscript to ensure all claims are evidence-based. As detailed in our response to the minor comment 3, we have added a quantitative benchmark comparing BIO-FIRE against LASSO and standard Random Forest (**Supplementary Figure 13**), providing concrete evidence for its superiority in balancing interpretability and performance. We have provided comprehensive documentation of our calibration curves and internal standard usage (**Supplementary Table 10**) to substantiate the reliability of our absolute quantification data. We believe these additions could strengthen the evidence for our claims and improve the overall rigor of the manuscript.

Minor Concerns:

#Comment 1: Code transparency. A publicly available repository (e.g., GitHub) with all analysis code and workflows is essential for reproducibility and community uptake.

#Response 1: Thanks for your comments. We have created a public GitHub repository containing all analysis code and workflows to ensure full reproducibility. The repository is available at: https://github.com/liyibai123/GC_Metabolomics.git.

#Comment 2: Tool versions missing. Multiple tools are cited (e.g., NMF, Boruta, Mfuzz, caret), but the exact versions are not reported. Versioning is required for reproducibility.

#Response 2: Thanks for your comments. The exact versions of all key software tools and packages used in this study are now listed in the revised manuscript as follows (Page 21, Line 677-683): *All computational workflows were implemented in R (version 4.4.2) leveraging critical packages including NMF (version 0.28) for consensus clustering, Boruta (version 9.0.0) for feature selection, Mfuzz (version 2.66.0) for time-series analysis, and caret (version 7.0.1) for machine learning model training and hyperparameter tuning. Additionally, the pheatmap (version 1.0.12) package was used for heatmap visualization, MASS (version 7.3.65) for LDA, pROC (version 1.18.5) for receiver operating characteristic curve (ROC) evaluation, and ggplot2 (version 3.5.2) for graphical representations.*

#Comment 3: Limited transparency around BIO-FIRE. Although BIO-FIRE integrates NMF and Boruta, there is no comparison with simpler feature-selection baselines (e.g., LASSO). The "recursive coverage optimization" criterion is described qualitatively; quantitative benchmarking of biological interpretability is absent.

#Response 3: We appreciate the reviewer's request for greater transparency and quantitative benchmarking. In response, we have conducted a comprehensive comparison between **BIO-FIRE** and two established baseline methods: **LASSO** and Standard Random Forest (**RF**).

We quantitatively benchmarked these algorithms across three critical dimensions: **Biological Interpretability, Statistical Power, and Diagnostic Performance.** The results of this comparison have been integrated into the revised manuscript as a dedicated subsection in the Results section (Page 11, Lines 326–339) and are visualized in the newly added **Supplementary Figure 13**. The detailed benchmarking process and interpretation of results are provided below:

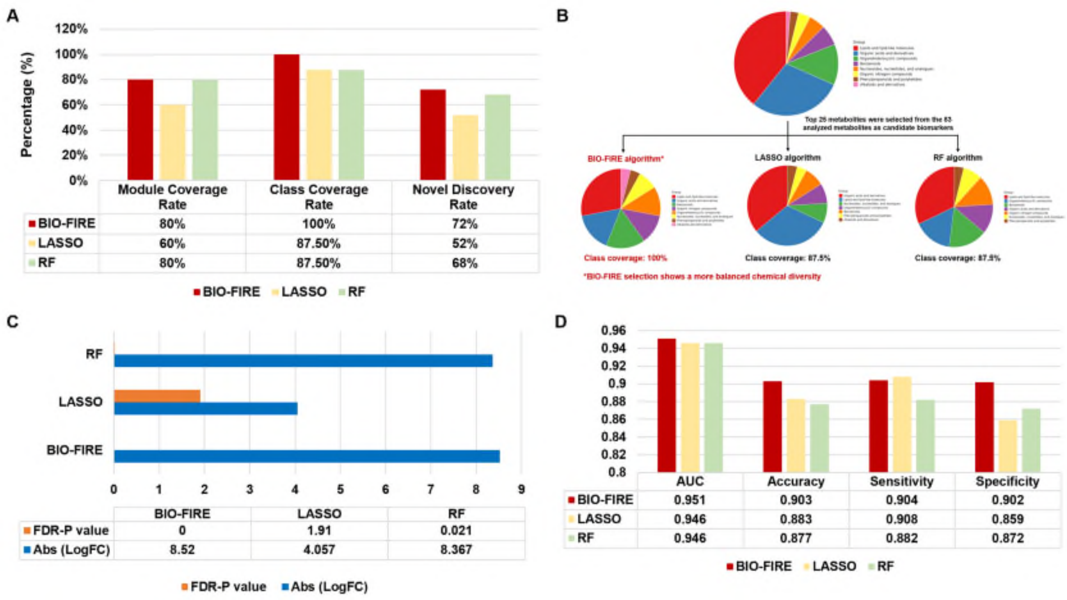

Supplementary Fig. 13 Comprehensive benchmarking of the BIO-FIRE algorithm against baseline feature selection methods. (A) Quantitative benchmarking of biological interpretability. **(B)** Changes of super class distribution before and after feature selection. **(C)** Quantitative benchmarking of statistical power. **(D)** Quantitative benchmarking of diagnostic performance.

1. Quantitative Benchmarking of Biological Interpretability

To assess how well each algorithm captures the biological complexity of gastric cancer, we analyzed the top 25 candidate biomarkers selected by each method (ranked by importance):

Functional Module Coverage: As shown in **Supplementary Fig. 13A**, BIO-FIRE achieved 80% coverage of the NMF-derived functional modules, significantly outperforming LASSO (60% coverage). This indicates that BIO-FIRE is more sensitive in capturing the diversity of the underlying metabolic network.

Chemical Class Diversity: Based on HMDB super-class annotations, BIO-FIRE selected metabolites spanning all 8 detected chemical classes (100% coverage), whereas LASSO and RF missed certain classes (87.5% coverage). As illustrated in the pie charts in **Supplementary Fig. 13B**, the BIO-FIRE panel exhibits a more balanced distribution across chemical classes, reflecting a more holistic representation of the metabolome.

Novelty of Discovery: By cross-referencing with our established "GC Biomarker Reference Set" (see Response to *Comment 1 of Reviewer 1*), we found that BIO-FIRE

achieved the highest Novel Discovery Rate (72%), compared to 52% for LASSO and 68% for RF. This demonstrates BIO-FIRE's strong capacity for *de novo* discovery, effectively identifying novel, data-driven signals rather than merely selecting well-known literature markers.

2. Benchmarking of Statistical Power

We evaluated the discriminatory power of the selected features by calculating cumulative statistical metrics. As shown in **Supplementary Fig. 13C**, the candidate panel selected by BIO-FIRE exhibited the strongest overall statistical signal, characterized by the lowest cumulative FDR P-value and the highest cumulative absolute Log2FC. This confirms that BIO-FIRE prioritizes features with robust individual discriminatory power.

3. Benchmarking of Diagnostic Performance

We compared the diagnostic efficacy of the final biomarker panels constructed using each method. To ensure a fair comparison, we applied the same "Recursive Coverage Optimization" stopping criterion across all three algorithms to determine the final panel size. As shown in **Supplementary Fig. 13D**, the BIO-FIRE panel achieved the highest performance in the independent validation cohort across key metrics, including AUC (0.951), accuracy (0.903), and specificity (0.902).

Collectively, these quantitative benchmarks demonstrate that BIO-FIRE outperforms conventional baselines not only in diagnostic accuracy but also in generating a biologically interpretable, diverse, and novel biomarker panel.

<Comments from Reviewer #3>

The manuscript by Bai et al describes the combination of targeted and untargeted metabolomics for gastric cancer biomarker discovery. The manuscript addresses important limitations of "traditional" metabolomics based biomarker discovery and proposes a novel framework. The manuscript is well written and should be of interest to the broad readership of Nature Communications.

Response: We appreciate your time and efforts in reviewing our manuscript, along with your positive and constructive comments aimed at strengthening our paper.

#Comment 1: Please define "interpretability-driven algorithm" what does this exactly mean exactly?

#Response 1: We thank the reviewer for this insightful question regarding the conceptual framework of our algorithm. We define an "interpretability-driven algorithm" as a biomarker selection workflow that prioritizes biological interpretability alongside statistical performance, distinguishing it from traditional "black-box" or purely "statistics-driven" methods.

Specifically, traditional feature selection methods (e.g., LASSO) primarily optimize statistical metrics like prediction accuracy or sparsity. While effective, these approaches lack explicit constraints for biological diversity, often resulting in biomarker panels that are clustered within a single dominant metabolic pathway while missing other critical metabolic alterations essential for a comprehensive diagnosis.

Our BIO-FIRE algorithm is "interpretability-driven" because it explicitly integrates interpretable metabolic functional modules (e.g., Lipid Metabolism, Host-Microbiome Co-metabolism, BCAA Metabolism) into the selection decision. By employing a "Recursive Coverage Optimization" strategy, the algorithm enforces the selection of a biomarker panel that spans multiple orthogonal NMF metabolic modules. This ensures that the final panel not only predicts cancer accurately but also provides a comprehensive view of the underlying metabolic dysregulation.

To enhance the clarity and rigor of our manuscript, we have refined the definition in three key sections:

Introduction (Page 4, Line 111-114): *Furthermore, we developed a customized interpretability-driven algorithm with NMF, defined as a strategy that prioritizes biological interpretability alongside statistical performance, distinguishing it from traditional statistics-driven methods.*

Methods (Page 20, Line 625-629): *Conceptually, BIO-FIRE is an interpretability-driven framework designed to overcome the limitations of traditional statistics-driven methods (e.g., LASSO), which often select redundant features from dominant pathways. Instead, BIO-FIRE explicitly enforces the selection of biomarkers spanning multiple orthogonal metabolic modules, ensuring comprehensive biological coverage.*

Discussion (Page 12, Line 383-386): *Unlike conventional black-box feature selection methods, this interpretability-driven workflow integrates biological relevance (via NMF module coverage), statistical importance, and panel conciseness for efficient signature generation.*

Additionally, to visually demonstrate the design rationale and operational process, we have added a detailed flowchart of the BIO-FIRE algorithm in **Supplementary Figure 14** (see details in our Response to *Comment 3 of Reviewer 3*). To substantiate the practical value of this "interpretability-driven" approach, we performed a comprehensive quantitative benchmarking against baseline methods (LASSO and standard Random Forest). As illustrated in **Supplementary Figure 13**, the results demonstrate that **BIO-FIRE outperforms conventional baselines not only in diagnostic accuracy but, more importantly, in generating a biologically interpretable, diverse, and novel biomarker panel**. For the detailed benchmarking process and interpretation, please refer to our Response to *Comment 3 of Reviewer 3* or *Minor Comment 3 of Reviewer 2*. Corresponding descriptions have also been added to the Results section (Page 11, Line 326-339).

#Comment 2: In line 157 the authors state “1,097 key signals…were identified” is that true? Were all these signals identified and assigned unambiguously to a metabolite? If I understand correctly the authors only used molecular formula matching without MS2. This is risky and might lead to misleading conclusions. Please clarify this. The matching

with standards is sound though.

#Response 2: We appreciate the reviewer's rigorous attention to terminology. We acknowledge that the statement "1,097 key signals···were identified" was imprecise. We intended to convey that these signals were detected as differentially expressed signals and prioritized for subsequent identification efforts, rather than being fully identified and assigned to a metabolite at that stage. We have corrected this phrasing in the revised manuscript to avoid ambiguity (Page 6, Line 158-160): *The union of these comparisons yielded 1,097 differentially expressed metabolic signals (defined as key signals), which were then prioritized for structural identification.*

As for clarification on identification confidence, we wish to reassure the reviewer that **there is no risk of misleading conclusions regarding the biomarkers used in our model**. For all metabolites included in the final relative-quantitative and absolute-quantitative targeted analysis, **we strictly adhered to Metabolomics Standards Initiative (MSI) Level 1 standards[3], utilizing both MS2 spectra and authentic standards for verification.** As illustrated in the funnel plot (Figure 2f), our identification strategy proceeded through three rigorous stages:

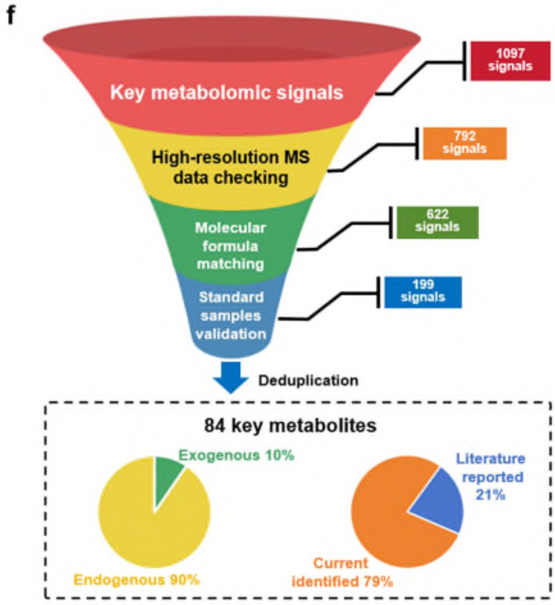

1. **High-Resolution MS Data Curation:** From the initial 1,097 differential signals, we prioritized features based on spectral quality. We leveraged high-resolution MS1 data (QTOF) coupled with high-sensitivity MS2 spectra (MIM-IDA-EPI from

QTRAP) to filter out noise and background artifacts. Only features with high signal-to-noise ratios and distinguishable fragmentation patterns were retained for structural elucidation.

2. **Structural Annotation (Molecular formula matching)**: For the retained signals, we performed molecular formula generation (using PeakView 2.2, error < 10 ppm) and structural matching against databases (e.g., ChemSpider, PubChem). Crucially, this step involved manual spectral interpretation, where we analyzed diagnostic fragment ions and neutral losses (e.g., loss of -COOH) to narrow down candidate isomers.
3. **Confirmatory Identification (Standards validation)**: The identity of candidate metabolites was definitively confirmed by running authentic chemical standards alongside the biological samples within the same analytical batch. A metabolite was considered "Identified" (MSI Level 1) only if it matched the standard in both Retention Time (RT) and MS2 fragmentation pattern, thereby eliminating potential ambiguities caused by inter-batch RT drift.

As conclusive proof, we have provided **Mirror Plots** for the 12 final diagnostic biomarkers in **Supplementary Figure 7** (also shown below). These plots demonstrate the high concordance between the MS2 spectra of the biological samples and the authentic standards, confirming structural identification. Correspondingly, we have made minor revision of the Results session (Page 7, Line 209-212) and Methods session (Page 20, Line 604-605, Line 610, Line 614-615) to explicitly detail this identification hierarchy.

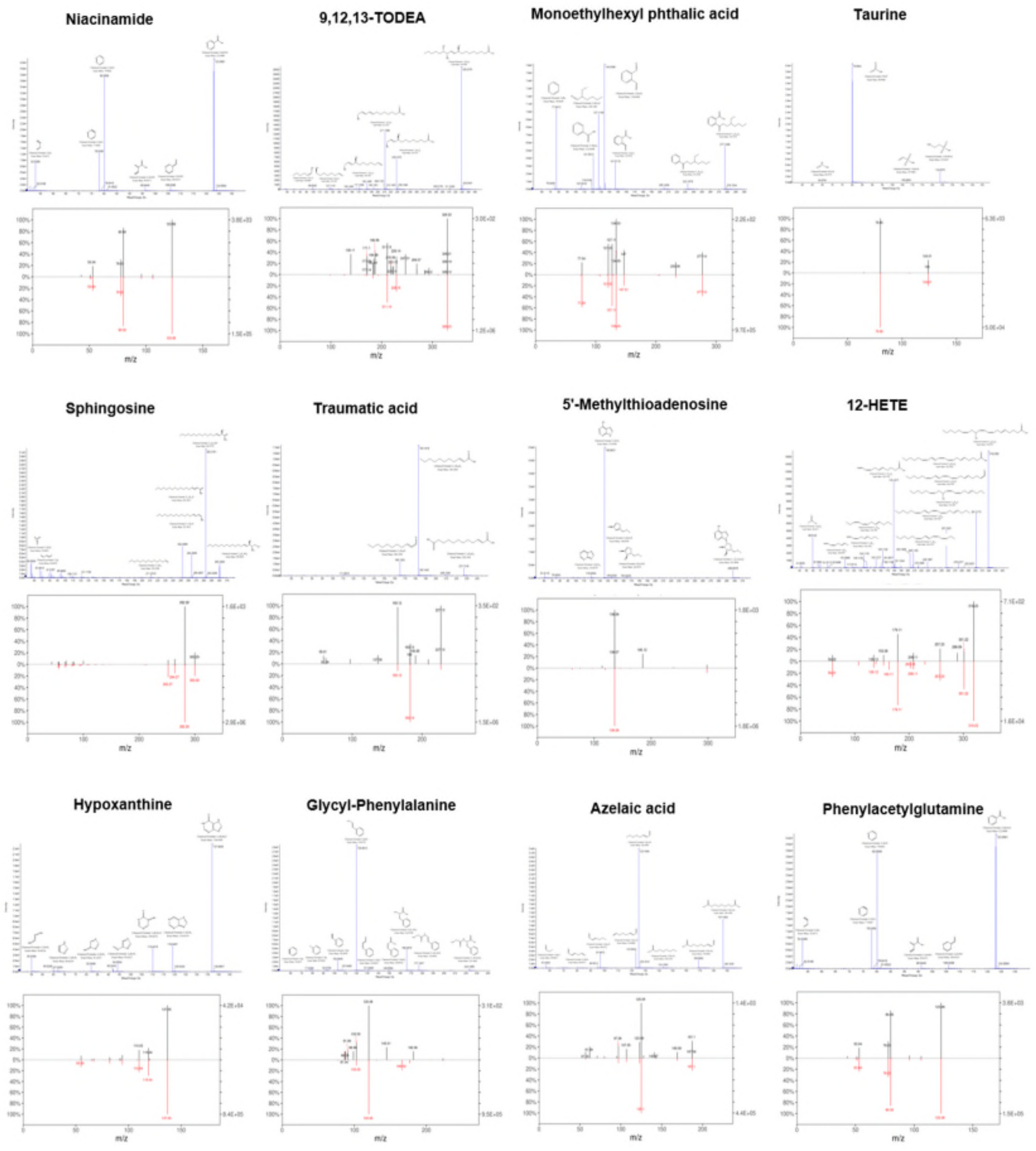

Supplementary Fig. 7 Representative MS/MS mirror plots verifying the structural identity of the 12 diagnostic biomarkers. The MS2 fragmentation spectra from the plasma samples (top blue panels) show a high degree of similarity to the spectra obtained from authentic chemical standards (bottom red panels), confirming the structural identification at Metabolomics Standards Initiative (MSI) Level 1 standard.

#Comment 3: What does not become entirely clear to me and should maybe be stressed a bit more, how does BIO-FIRE work what does it exactly do and why is it advantageous when compared to established methods?

#Response 3: We thank the reviewer for this request. We have clarified the algorithm's mechanism and demonstrated its advantages as follows:

1. Mechanism Clarification: How does BIO-FIRE work?

As visually summarized in the newly added **Supplementary Figure 14** (BIO-FIRE Flowchart, also shown as below), the algorithm operates through three sequential stages designed to integrate biological structure with statistical power:

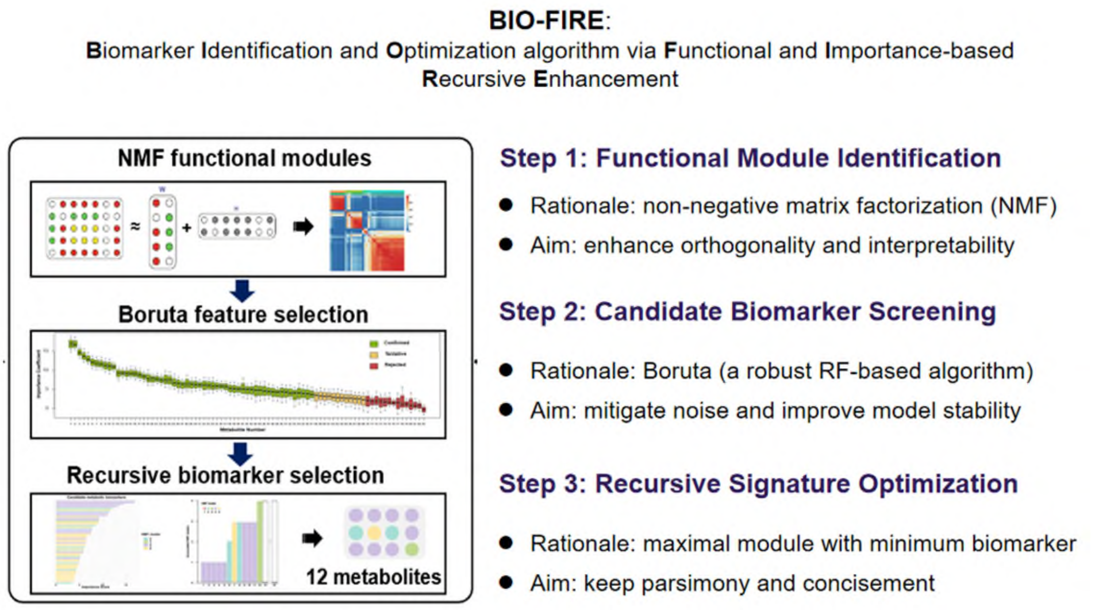

Supplementary Fig. 14 Flowchart illustrating the BIO-FIRE biomarker discovery framework.

Step 1: Functional Module Identification: We first decompose the high-dimensional metabolomic data into biologically coherent functional modules using Non-negative Matrix Factorization (NMF). This step transforms the feature space from individual metabolites to orthogonal biological axes (e.g., Lipid Metabolism, BCAA Metabolism), enhancing interpretability.

Step 2: Candidate Biomarker Screening: We then apply the Boruta algorithm to identify statistically significant features. By comparing importance scores against permuted "shadow features," Boruta rigorously filters out noise, ensuring that only robust candidates are retained for the next step.

Step 3: Recursive Signature Optimization: Instead of simply selecting the top-ranked markers, BIO-FIRE employs a recursive greedy strategy. Candidate metabolites are added sequentially based on their importance only if they contribute to covering a new NMF functional module. This process continues until maximal module coverage is achieved with the minimum number of markers, ensuring both parsimony and

biological diversity.

2. Advantage Demonstration: Why is it superior to established methods?

The superiority of BIO-FIRE stems from its unique design philosophy (prioritizing biological information density over mere statistical sparsity) and is confirmed by our quantitative benchmarking:

- **<u>Theoretical Advantage:</u>** Unlike traditional methods (e.g., LASSO) that may select redundant features from a single dominant pathway, BIO-FIRE enforces coverage across diverse metabolic modules (as detailly explained in our Response to *Comment 1 of Reviewer 3*). This ensures the panel captures the full systemic heterogeneity of the disease.
- **<u>Empirical Superiority:</u>** To quantitatively demonstrate the advantage of BIO-FIRE, we performed a comprehensive benchmarking against two established baselines: LASSO and standard Random Forest (RF). As detailed in our Response to *Minor Comment 3 of Reviewer 2* and illustrated in **Supplementary Figure 13**, BIO-FIRE exhibits distinct superiorities:

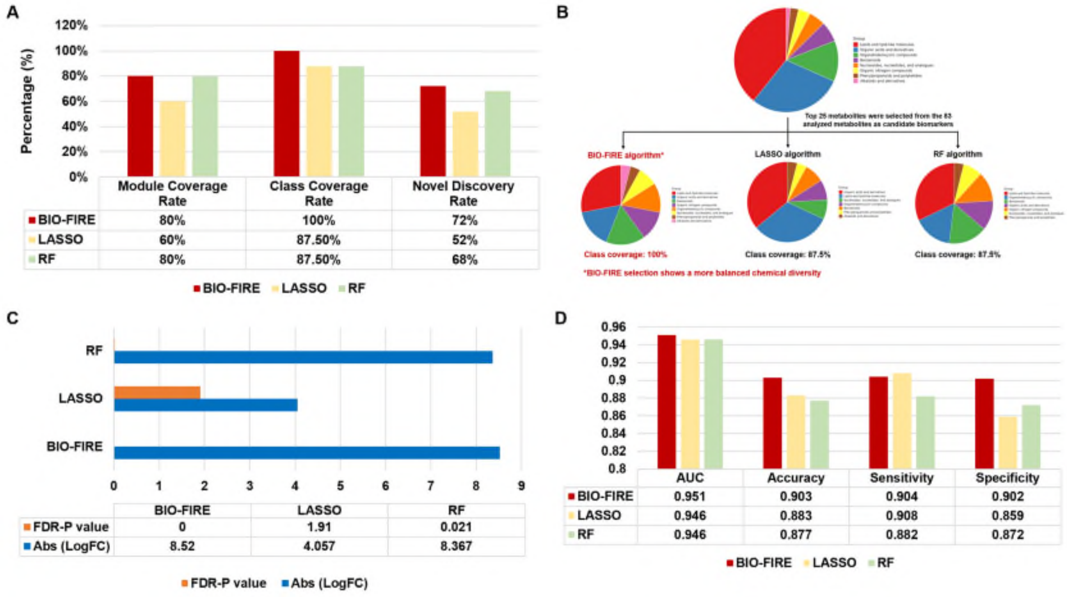

Supplementary Fig. 13 Comprehensive benchmarking of the BIO-FIRE algorithm against baseline feature selection methods. (A) Quantitative benchmarking of biological interpretability. **(B)** Changes of super class distribution before and after feature selection. **(C)** Quantitative benchmarking of statistical power. **(D)** Quantitative benchmarking of diagnostic performance.

(1) **Higher Interpretability:** BIO-FIRE achieved 80% coverage of functional modules,

whereas LASSO covered only 60%, indicating that traditional methods may miss critical biological axes due to redundancy.

(2) **Greater Novelty:** BIO-FIRE demonstrated a higher rate of discovering novel biomarkers (72%) compared to baselines (52% for LASSO), highlighting its capacity for data-driven discovery beyond prior knowledge.

(3) **Better Performance:** In the independent validation cohort, the BIO-FIRE panel achieved the highest AUC (0.951) compared to LASSO (0.946) and RF (0.946), proving that incorporating biological structure improves model generalization.

We have incorporated these comparative results into the Results section (Page 11, Line 326-339) to substantiate the advantages of our method.

#Comment 4: In the methods section, this sentence seems flawed "For absolute-quantitative metabolomic profiling, 50 μL of plasma was combined with 150 μL of the extraction solvent and vortexed for 3 minutes. Following centrifugation under identical condition, 170 μL of the supernatant was transferred to a 96-well plate for protein precipitation." Wasn't the protein precipitated by adding the extraction solvent?

#Response 4: We thank the reviewer for this keen observation and apologize for the lack of clarity in our original description.

We agree that protein precipitation primarily occurs upon the initial addition of the organic extraction solvent. The specific step mentioned in the query referred to a secondary purification process. Instead of a simple transfer, the supernatant was loaded onto a specialized filter-based protein precipitation plate (designed for phospholipid removal). A positive pressure manifold was then applied to drive the supernatant through the filter. This additional rigorous step was implemented to effectively deplete interfering phospholipids and residual particulates, thereby minimizing matrix effects and enhancing the robustness of absolute quantification.

We have revised the "Metabolite extraction" section (Page 16, Line 509-513) to provide a precise description of this protocol: *Following centrifugation under identical conditions, 170 μL of the supernatant was loaded onto a dedicated filter-based protein precipitation plate for a secondary clean-up step to effectively remove phospholipids*

and residual particulates. A positive pressure manifold was then applied to drive the supernatant through the filter membrane, and the purified filtrate was collected directly into a 96-well collection plate.

#Comment 5: Please define, MIM-IDA-EPI

#Response 5: We thank the reviewer for requesting this clarification. MIM-IDA-EPI stands for **M**ultiple **I**on **M**onitoring - **I**nformation **D**ependent **A**cquisition - **E**nhanced **P**roduct **I**on scanning mode. This is a sophisticated hybrid data acquisition strategy performed on the QTRAP mass spectrometry system, which enables the simultaneous acquisition of quantitative data and qualitative MS/MS spectra in a single run. The methodology was originally established for widely targeted metabolomics by Chen et al[4].

Specifically, the workflow operates as follows:

MIM (Survey Scan for Quantification): The instrument performs Multiple Ion Monitoring, where both Q1 and Q3 are set to the same mass (e.g., 50/50, 51/51... up to 1000/1000 Da) with minimal collision energy (approx. 5 eV) in Q2. This allows precursor ions to pass through without fragmentation, functioning similarly to a Selected Ion Monitoring (SIM) scan to provide sensitive quantitative data.

IDA (Trigger): The Information Dependent Acquisition (IDA) logic monitors the MIM signal intensity in real-time. Once the intensity exceeds a pre-set threshold, it triggers the subsequent scan.

EPI (Dependent Scan for Identification): Upon triggering, an Enhanced Product Ion (EPI) scan is executed using the linear ion trap functionality to capture high-resolution MS/MS fragmentation spectra for the corresponding metabolite.

We have defined the full term MIM-IDA-EPI and cited the foundational reference[4] in the Methods section (Page 17, Line 522-523) of the main text. Furthermore, to ensure full reproducibility without disrupting the flow of the main text, we have provided the detailed operational parameters (e.g., scan range, dwell time, cycle time) in **the legend of Supplementary Fig. 2**: *Technical details of MIM-IDA-EPI analysis: The Multiple Ion Monitoring (MIM) scan served as a survey scan to trigger the Information-*

Dependent Acquisition (IDA) of Enhanced Product Ion (EPI) spectra. In the MIM mode, a minimal collision energy (CE) of 5 eV was applied in Q2, allowing precursor ions isolated in Q1 to pass through to Q3 without fragmentation for detection. Upon exceeding an intensity threshold, EPI scans were triggered and acquired with a declustering potential (DP) of 40 V and CE of 40 V to generate MS/MS spectra. A stepwise MIM-EPI strategy was adopted covering a mass range from m/z 50 to 1000 in positive ion mode. The scan range was segmented with a step size of 1 Da (e.g., 50/50, 51/51... to 1000/1000 Da). Each MIM transition was monitored with a 5-ms dwell time and a 5-ms pause time. Each experiment monitored 30 MIM transitions, with product ions scanned from 50 to 1000 Da in Q3. The total cycle time for one scan was approximately 1.9 s. In total, 32 LC/MS runs were performed to cover the entire mass range.

#Comment 6: In the absolute quantification work-flow did the authors use labelled internal standards? Given several of the identified biomarkers are bile acids this is highly important. From experience in my laboratory we even know that absolute BA quantification is only possible by using individual labelled IS for each BA metabolite. Please clarify and comment. Moreover, if no labelled IS were used how solid are the so called absolute quantities?

#Response 6: We thank the reviewer for raising this essential point regarding quantification rigor, particularly for bile acids (BAs). We fully agree that the use of stable isotope-labelled internal standards (IS) is critical for accurate absolute quantification, especially for compounds like BAs that are highly susceptible to matrix effects. We clarify our quantification strategy as follows:

1. Rigorous IS Calibration for Bile Acids

Regarding the reviewer's specific concern, we confirm that all major detected BAs were quantified using their specific isotope-labelled internal standards. For example, cholic acid (CA) was corrected by [$^2H_4$]-CA, chenodeoxycholic acid (CDCA) by [$^2H_4$]-CDCA, and so forth. **Response Letter Table 2** (below) summarizes the IS assignments and performance metrics for all detected BAs. Standard calibration curves were

constructed by plotting the peak area ratio of the authentic standard to its corresponding IS (Y-axis) against the concentration of authentic standard (X-axis), yielding excellent linearity. **Response Letter Figure 2** shows representative calibration curves for two key BAs.

Response Letter Table 2. Internal standard (IS) assignment and performance metrics of calibration curves for bile acids.

Metabolite Name	Abbreviation	IS Name	Regression Equation	Coefficient of Determination	Linear Dynamic Range(ng/ml)
Glycochenodeoxycholic acid	GCDCA	[2H4]-GCDCA	y = 22.25488 x + 18.40743	0.99510	0.125-625
Chenodeoxycholic acid	CDCA	[2H4]-CDCA	y = 0.00538 x + 0.00109	0.99812	0.125-625
Deoxycholic acid	DCA	[2H4]-DCA	y = 0.00267 x + 0.00609	0.99833	0.5-10000
Cholic acid	CA	[2H4]-CA	y = 0.00801 x + 0.00346	0.99778	0.0625-625
Glycoursodeoxycholic acid	GUDCA	[2H4]-GUDCA	y = 0.88796 x + 0.05490	0.99820	0.125-625
Isohyodeoxycholic acid	isoHDCA	[2H4]-isoHDCA	y = 8.09063e-4 x + 5.42192e-4	0.99936	3.125-625
Hyodeoxycholic acid	HDCA	[2H5]-HDCA	y = 0.00473 x + 0.00302	0.99798	0.125-625
Ursodeoxycholic acid	UDCA	[2H4]-UDCA	y = 0.00183 x + 0.00100	0.99887	0.125-625
Glycolithocholic acid	GLCA	[2H4]-GLCA	y = 0.00147 x + 1.72935e-4	0.99923	0.0125-625
Hyocholic acid	HCA	[2H4]-HCA	y = 0.01270 x + 2.61830e-4	0.99487	0.03125-625

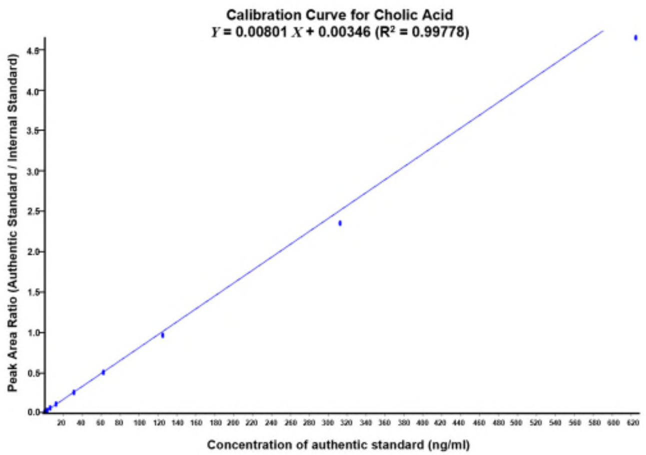

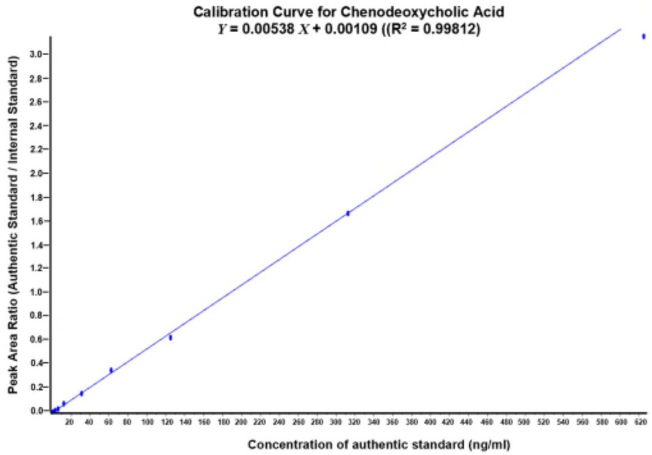

Response Letter Figure 2. Representative calibration curves for key bile acids demonstrating robust quantification. The calibration curves for Cholic Acid (left) and Chenodeoxycholic Acid (right) were constructed using the internal standard method. The plots display the linear relationship between the concentration and the peak area ratio.

2. Broad Use of IS for Other Metabolites:

Beyond BAs, we implemented a broad isotope-labelled IS strategy for precise quantification across diverse metabolic classes. Our selection of IS followed a strategic hierarchy: Firstly, we systematically covered metabolite classes known to present specific analytical challenges. For instance, we employed matched IS for endogenous nucleosides and bases (e.g., [2H4]-Hypoxanthine, [13C5]-Inosine, Uridine) to correct for variable ionization efficiency; for conjugated steroids (e.g., [13C5]-Pregnanediol 3-O-glucuronide) and sulfates (e.g., [2H5]-Indoxyl Sulfate) to counteract significant matrix effects and in-source fragmentation; and for sphingolipids (e.g., [13C2,2H2]-Sphingosine) to compensate for variable extraction recovery. Additionally, for critical analytes of other classes (e.g., specific amino acids and organic acids in core pathways), we also utilized matched IS whenever commercially available to maximize overall quantitative precision. Ultimately, 55 out of 83 (66%) targeted metabolites were quantified using their matched isotope-labelled IS.

To improve rigor and transparency, we have provided all IS names and calibration performance metrics in **Supplementary Table 10** (screenshot shown as below). This extensive use of IS facilitates the accuracy and reliability of our absolute quantification.

Supplementary Table 10. Assignment of matched isotope-labelled internal standards (IS) for 56 metabolites.				
**Metabolite ID**	**Metabolite Name**	**Class**	**CAS No.**	**IS Name**
GC_P_B29_4	Piperine	Alkaloids and derivatives	94-62-2	[2H10]-Piperine
GC_P_B112	Glutamine	Amino acids and derivatives	56-85-9	[2H5]-Glutamine
GC_P_B101	L-Valine	Amino acids and derivatives	72-18-4	[2H8]-Valine
GC_P_B55_1	L-Arginine	Amino acids and derivatives	74-79-3	[2H7]-Arginine
GC_P_B103_1	Isoleucine	Amino acids and derivatives	73-32-5	[15N]-Isoleucine
GC_N_B05	L-Tryptophan	Amino acids and derivatives	73-22-3	[2H5]-Tryptophan
GC_P_B130	Ornithine	Amino acids and derivatives	70-26-8	[2H7]-Ornithine
GC_P_B15	Methionine	Amino acids and derivatives	63-68-3	[2H3]-Methionine
GC_N_B01	Taurine	Amino acids and derivatives	107-35-7	[13C2]-Taurine
GC_N_B23	Phenylacetylglutamine	Amino acids and derivatives	28047-15-6	[2H5]-Phenylacetylglutamine
GC_N_B38	p-Cresol sulfate	Benzenoids	3233-58-7	[2H4]-p-Tolyl Sulfate
GC_N_B06	Hippuric acid	Benzenoids	495-69-2	[2H5]-Hippuric Acid
GC_N_B36	Phenyllactic acid	Benzenoids	828-01-3	[2H3]-Phenyllactic acid
GC_P_B51	1,3-Diphenylguanidine	Benzenoids	102-06-7	[2H103]-1,3-Diphenylguanidine
GC_P_B24	Liothyronine	Benzenoids	6893-2-3	[13C6]-Liothyronine
GC_N_B30	Phenoxyacetic acid	Benzenoids	122-59-8	[2H5]-Phenoxyacetic acid
GC_N_B46	Salicylic acid	Benzenoids	69-72-7	[2H4]-Salicylic Acid
GC_N_B66_1	Monoethylhexyl phthalic acid	Benzenoids	4376-20-9	[2H4]-Monoethylhexyl phthalic acid
GC_N_B42	Glycochenodeoxycholic acid	Bile acids, alcohols and derivatives	640-79-9	[2H4]-Glycochenodeoxycholic Acid
GC_N_B11	Chenodeoxycholic acid	Bile acids, alcohols and derivatives	474-25-9	[2H4]-Chenodeoxycholic Acid
GC_N_B12	Deoxycholic acid	Bile acids, alcohols and derivatives	83-44-3	[2H4]-Deoxycholic Acid
GC_N_B09_4	Cholic acid	Bile acids, alcohols and derivatives	81-25-4	[2H4]-Cholic Acid
GC_N_B10	Glycoursodeoxycholic acid	Bile acids, alcohols and derivatives	64480-66-6	[2H4]-Glycoursodeoxycholic Acid
GC_P_B41	Isohyodeoxycholic acid	Bile acids, alcohols and derivatives	570-84-3	[2H4]-Isohyodeoxycholic acid
GC_N_B14	Hyodeoxycholic acid	Bile acids, alcohols and derivatives	83-49-8	[2H5]-Hyodeoxycholic Acid
GC_P_B13	Ursodeoxycholic acid	Bile acids, alcohols and derivatives	128-13-2	[2H4]-Ursodeoxycholic acid
GC_N_B21	Glycolithocholic acid	Bile acids, alcohols and derivatives	474-74-8	[2H4]-Glycolithocholic acid
GC_N_B48_3	Hyocholic acid	Bile acids, alcohols and derivatives	547-75-1	[2H4]-Hyocholic acid
GC_N_B02	Azelaic acid	Fatty Acyls	123-99-9	[2H14]-Azelaic Acid
GC_N_B40	Docosahexaenoic acid	Fatty Acyls	6217-54-5	[2H5]-Docosahexaenoic acid
GC_P_B58	Decanoylcarnitine	Fatty Acyls	1492-27-9	[2H3]-Decanoylcarnitine
GC_P_B27	Octanoylcarnitine	Fatty Acyls	25243-95-2	[2H3]-Octanoylcarnitine
GC_N_B63	12-HETE	Fatty Acyls	71030-37-0	[2H8]-12-HETE
GC_N_B82_1	Coriolic acid	Fatty Acyls	10219-69-9	[2H43]-Coriolic acid
GC_P_B26	Dodecanoylcarnitine	Fatty Acyls	25518-54-1	[2H3]-Dodecanoylcarnitine
GC_P_B08	Indole-3-propionic acid	Indoles and derivatives	830-96-6	[2H2]-Indole-3-propionic Acid
GC_N_B37_1	Indoxyl sulfate	Indoles and derivatives	487-94-5	[2H5]-Indoxyl Sulfate
GC_P_B18	Indole-3-carboxaldehyde	Indoles and derivatives	487-89-8	[13C]-Indole-3-Carboxaldehyde
GC_N_B108	Succinic acid	Organic acids and derivatives	110-15-6	[2H4]-Succinic Acid
GC_N_B60	Menthol-glucoronide	Prenol lipids	79466-08-3	[2H4]-Menthol-glucoronide
GC_P_B04_1	Hypoxanthine	Purines and derivatives	68-94-0	[2H4]-Hypoxanthine
GC_P_B45_1	Caffeine	Purines and derivatives	58-08-2	[2H3]-Caffeine
GC_N_B35	Inosine	Purines and derivatives	58-63-9	[13C5]-Inosine
GC_P_B47	Theophylline	Purines and derivatives	58-55-9	[2H6]-Theophylline
GC_P_B19	Paraxanthine	Purines and derivatives	611-59-6	[2H3]-Paraxanthine
GC_P_B34	5'-Methylthioadenosine	Purines and derivatives	2457-80-9	[2H3]-5'-Methylthioadenosine
GC_N_B20	1-Methylxanthine	Purines and derivatives	6136-37-4	[2H3]-1-Methylxanthine
GC_P_B44	Niacinamide	Pyridines and derivatives	98-92-0	[2H4]-Nicotinamide
GC_P_B114	1-Methylnicotinamide	Pyridines and derivatives	3106-60-3	[2H3]-1-Methylnicotinamide
GC_N_B03	Uridine	Pyrimidines and derivatives	58-96-8	[13C]-Uridine
GC_P_B22	Sphingosine	Sphingolipids	123-78-4	[13C2,2H2]-Sphingosine
GC_P_B28	Sphinganine	Sphingolipids	764-22-7	[2H7]-Sphinganine
GC_N_B68_1	Androsterone glucuronide	Steroids and steroid derivatives	1852-43-3	[2H4]-Androsterone glucuronide
GC_N_B83_1	Pregnanediol 3-O-glucuronide	Steroids and steroid derivatives	1852-49-9	[13C5]-Pregnanediol 3-O-glucuronide
GC_P_B16	Corticosterone	Steroids and steroid derivatives	50-22-6	[2H4]-Corticosterone

3. Justification for External Standard (ES) Quantification:

For the remaining subset of metabolites (28/83), we employed the external standard method due to the lack of commercially available stable isotope-labelled standards and the prohibitive complexity of *de novo* synthesis. To ensure quantitative accuracy without internal standards, we implemented a rigorous "Chromatography-Preparation Synergy" strategy to minimize matrix effects:

(1) **Proactive Matrix Removal:** As detailed in Response 4, we utilized phospholipid removal plates to physically deplete interfering lipids. Literature confirms that

phospholipid depletion significantly reduces background complexity and ion suppression, providing a stable ionization environment for subsequent quantitation[5].

(2) **Optimized Chromatographic Separation:** We utilized a Waters ACQUITY UPLC HSS T3 column, engineered for superior polar retention. Compared to conventional columns (e.g., BEH C18 or HILIC), the HSS T3 column offers superior retention for polar analytes, effectively separating them from early-eluting salts and phospholipids. This separation significantly mitigates ion suppression for polar classes such as amino acids and organic acids[6,7].

(3) **Analyte Stability:** Many of the ES-quantified metabolites, such as creatinine and Carnitine C7:0, are high-abundance endogenous compounds with well-characterized ionization behaviors. Their chemical stability, combined with our optimized separation (HSS T3) and clean-up (phospholipid removal) protocols, ensures that matrix effects are minimal even without isotopic correction.

In summary, we believe the potential limitations of external calibration are well-controlled within our workflow. By combining extensive IS coverage with proactive matrix removal and optimized chromatography, our integrated approach represents a leading standard for high-throughput absolute quantitative metabolomics in the field.

To enhance rigor and transparency, we have expanded the Methods section (Page 17-18, Line 549-564) to explicitly detail this hybrid quantification strategy. Furthermore, we have added **Supplementary Table 11**, which lists the regression equations, coefficients of determination, linear dynamic ranges, limits of detection (LOD), and limits of quantification (LOQ) for all 83 quantified metabolites.

References:

1. Sung, H., *et al.* Global Cancer Statistics 2020: GLOBOCAN Estimates of Incidence and Mortality Worldwide for 36 Cancers in 185 Countries. *CA Cancer J Clin* **71**, 209-249 (2021).
2. Hamamoto, R., *et al.* Application of non-negative matrix factorization in oncology: one approach for establishing precision medicine. *Brief Bioinform* **23**(2022).
3. Alseekh, S., *et al.* Mass spectrometry-based metabolomics: a guide for annotation, quantification and best reporting practices. *Nat Methods* **18**, 747-

756 (2021).
4. Chen, W., *et al.* A novel integrated method for large-scale detection, identification, and quantification of widely targeted metabolites: application in the study of rice metabolomics. *Mol Plant* **6**, 1769-1780 (2013).
5. Carmical, J. & Brown, S. The impact of phospholipids and phospholipid removal on bioanalytical method performance. *Biomed Chromatogr* **30**, 710-720 (2016).
6. Chen, W.-L., Hsu, T.-F. & Chen, C.-Y. Performance Liquid Chromatography/Tandem Mass Spectrometry. *Journal of AOAC INTERNATIONAL* **94**, 872-877 (2019).
7. New, L.-S. & Chan, E.C.Y. Evaluation of BEH C18, BEH HILIC, and HSS T3 (C18) Column Chemistries for the UPLC-MS-MS Analysis of Glutathione, Glutathione Disulfide, and Ophthalmic Acid in Mouse Liver and Human Plasma. *Journal of Chromatographic Science* **46**, 209-214 (2008).

Point-by-Point Response to Reviewer's Comments

Reviewer #1 (Remarks to the Author):

The authors have clearly made meaningful improvements to the manuscript based on earlier reviewer feedback. Specifically, the expanded methodological descriptions, additional explanations around the hybrid metabolomics workflow, and improved figure organization all contribute to better clarity and readability.

Response: We sincerely thank the reviewer for recognizing the improvements made to our manuscript. We deeply appreciate the constructive feedback, which has been instrumental in strengthening the clarity, rigor, and readability of our study. We have carefully addressed your remaining insightful comments as detailed below.

1. BIO-FIRE Algorithm Still Needs Clearer Consolidation

The manuscript explains the components of BIO-FIRE (NMF, Boruta, recursive optimization), but the description remains scattered across multiple sections. A short, unified paragraph that outlines:

- the sequential steps,
- inputs and outputs of each stage, and
- the stopping criteria for the recursive step

would make the workflow much easier to follow for readers attempting to understand or replicate the method.

Response: We completely agree with the reviewer that a unified summary detailing the inputs, outputs, and stopping criteria is essential for readability and reproducibility. To address this precisely without introducing redundancy across the manuscript, we have added a concise, consolidated overview paragraph directly into the Methods section (under "BIO-FIRE for signature generation"), immediately preceding the detailed operational descriptions.

The added paragraph explicitly addresses the three bullet points requested (Page 20, Line 640-649): *To facilitate reproducibility, the operational workflow of BIO-FIRE operates through three sequential stages with strictly defined inputs, outputs, and a designated stopping criterion (see flowchart in Supplementary Fig. 14). Briefly, Stage 1 takes row-sum normalized absolute*

quantitative data as input and outputs biologically orthogonal NMF functional modules. Stage 2 inputs the absolute quantitative data alongside clinical labels to output a narrowed list of statistically significant candidate metabolites. Stage 3 takes these Boruta-ranked candidates and their assigned NMF modules as input to recursively optimize the biomarker panel. This recursive selection terminates upon a strict stopping criterion: when the addition of subsequent ranked metabolites yields no further increase in unique NMF module coverage.

This unified paragraph, combined with the BIO-FIRE flowchart (Supplementary Fig. 14), now provides a clear and highly accessible blueprint of the entire algorithm for readers attempting to replicate our method.

2. Add Key Statistics for the 12 Biomarkers to Support Fig. 4b–m

While the visual progression patterns are informative, adding a small summary table (or statistics in the supplement) for each of the 12 final metabolites would make Fig. 4 more self-explanatory. Also, given that the authors have multiple different classes, multiple testing inflation might help account for the data.

Response: We thank the reviewer for this highly constructive suggestion. We agree that providing a quantitative summary alongside the visual trajectories in Figure 4 is necessary, and that rigorously accounting for multiple testing inflation is critical given the four clinical classes evaluated in our study.

To address this, we have generated a new supplementary table (Supplementary Data 5), which provides a comprehensive statistical summary for each of the 12 selected biomarkers. Specifically, we first applied the Kruskal-Wallis test to assess overall variation across all four groups. To account for multiple testing inflation, we calculated P-values using the Wilcoxon rank-sum test for the overall diagnostic groups (GC vs. NGC) as well as for all pairwise comparisons among the four specific classes (e.g., HC vs. BGD, BGD vs. EGC). We then applied the Benjamini-Hochberg (BH) procedure globally across all these pairwise tests for the 12 biomarkers to compute the False Discovery Rate (FDR) adjusted P-values. Median relative abundances and fold changes (FC) for each comparison are also included.

To guide reader interpretation, we have updated the main text to reference this new table, mathematically supporting the visual trajectories presented in Fig. 4. The revised text reads as

follows (Page 8, Line 244-247): *Detailed descriptive statistics, fold changes, and significance levels for the 12 biomarkers across all pairwise clinical comparisons, including P-values adjusted for multiple testing via the Benjamini-Hochberg method, are summarized in Supplementary Data 5.*

Minor Changes:

1. Improving Readability:

There are multiple sentences which are difficult to read through and understand. Here are some examples:

“Conceptually, BIO-FIRE is an interpretability-driven framework designed to overcome the limitations of traditional statistics-driven methods (e.g., LASSO), which often select redundant features from dominant pathways.” - what does interpretability-driven mean?

Response: We apologize if this concept was not immediately clear. By "interpretability-driven", we refer to an approach that prioritizes biological coherence alongside statistical importance, in contrast to black-box methods that select features purely based on numerical thresholds.

The revised text reads as follows (Page 20, Line 627-633): *Conceptually, BIO-FIRE is an interpretability-driven framework—an approach that prioritizes biological coherence (via NMF modules) alongside statistical importance, in contrast to black-box methods that select features purely based on numerical thresholds. This design overcomes the limitations of traditional statistics-driven methods (e.g., LASSO), which often select redundant features from dominant pathways. Instead, BIO-FIRE explicitly enforces the selection of biomarkers spanning multiple orthogonal metabolic modules, ensuring comprehensive biological coverage.*

“To validate the methodological superiority of BIO-FIRE, we benchmarked it against established baselines: LASSO and standard RF.” – was RF used as classifier or regression? How exactly?

Response: We apologize for the imprecise phrasing. In this specific benchmarking context, the standard Random Forest (RF) was utilized strictly as a feature selection tool (analogous to LASSO), rather than as the final predictive classifier or regression model. We utilized the RF algorithm to compute feature importance scores to rank and screen the top candidate

metabolites, and then compared the biological interpretability and statistical power of the biomarkers selected by RF against those selected by LASSO and our BIO-FIRE algorithm.

We have corrected the text to explicitly state its role as a feature selection tool (Page 11, Line 328-330): *To validate the methodological superiority of BIO-FIRE, we benchmarked it against established feature selection baselines: LASSO and standard Random Forest (RF)-based feature importance ranking.*
